# Genome-wide DNA methylation changes after 24 hours at high altitude

**DOI:** 10.1093/eep/dvag004

**Published:** 2026-02-09

**Authors:** Shyleen Frost, Kathy Pham, Erica C Heinrich

**Affiliations:** Institute for Systems Biology, Seattle, WA 98109, United States; Division of Biomedical Sciences, University of California, Riverside School of Medicine, Riverside, CA 92521, United States; Division of Biomedical Sciences, University of California, Riverside School of Medicine, Riverside, CA 92521, United States

**Keywords:** hypoxia, high altitude, epigenetics, DNA methylation

## Abstract

High altitude presents a significant environmental stressor in the form of hypobaric hypoxia. The body responds to this condition with various acclimatization mechanisms, yet the role of epigenetic modifications, particularly DNA methylation, remains unclear. To address this gap, we investigated DNA methylation patterns in response to acute high-altitude exposure. Twelve healthy sea-level residents, aged 19–32 years, traveled to 3800 m, and DNA from peripheral blood mononuclear cells was collected both at sea level and after 24 h at high altitude. DNA methylation was assessed using the Illumina MethylationEPIC array. We identified 58,046 differentially methylated positions at high altitude compared to sea level, with a large majority of these sites showing increased methylation levels at high altitude, supporting the hypothesis that acute exposure to hypoxia may result in global hypermethylation. Notably, differentially methylated sites were located in genes enriched for pathways related to the hypoxia-inducible factor (HIF) pathway, such as “Notch signaling” and “AKT1 signaling in cancer.” Moreover, several pathways associated with calcium regulation and DNA damage repair were implicated, suggesting an association between DNA methylation and calcium processes affected by hypoxia. In addition to single positions, we explored differentially methylated regions, resulting in top differentially methylated regions being associated with calcium processes, zinc finger proteins, glucose processes, and erythropoiesis. These findings provide insight into how short-term environmental hypoxia may influence the human epigenome, highlighting DNA methylation as a dynamic marker of environmental exposure.

## Introduction

High altitude presents a significant environmental stressor in the form of hypobaric hypoxia. At high elevations, arterial oxygen partial pressure and hemoglobin oxygen saturation can begin to decline substantially, leading to decreased oxygen availability affecting multiple biological systems. In response, the body undergoes a series of plastic physiological changes that increase the rate of oxygen delivery to tissues. These changes include increased minute ventilation and ventilatory chemoreflex sensitivity [1–3], cardiovascular changes including increased red blood cell production [4–6], and modifications in metabolic energy production pathways [7, 8]. While the physiological changes occurring during high-altitude acclimatization are well characterized, including their molecular and cellular mechanisms, less is known about how such exposures impact epigenetic regulation—an important mechanism by which cells respond to environmental conditions.

Epigenetic modifications are chemical alterations to DNA, or its associated proteins such as histones, which can impact gene expression without altering the underlying DNA sequence. Such modifications can be stable and heritable, and they play a crucial role in regulating gene expression during development and in response to environmental stimuli. DNA methylation is an epigenetic modification that involves the addition of a methyl group to cytosine residues in CpG dinucleotides and is most commonly found in CpG islands (CGIs), which are densely packed regions of CpG sites. CGIs are found in the promoter region of nearly 70% of all genes [9]. Changes in DNA methylation can influence transcription factor binding, alternative splicing, and other mechanisms, which can change gene expression patterns [10–12].

In the context of high-altitude hypoxia, several reviews suggest that environmental oxygen availability may shape the epigenome [13–16]. Notably, Childebayeva *et al*. [17] examined climbers ascending Mt Everest and found there were changes in targeted hypoxia-inducible factor (HIF) pathway genes including increased methylation levels at high altitude in *EPAS1* (encoding HIF-2α) and *PPARa*. They also found decreased methylation levels at high altitude as compared to baseline values in *LINE-1, EPO*, and *RXRa* [17]. This study was followed by an additional epigenome-wide analysis during ascent including day 0 at 1400 m and day 7 at 4240 m. The results of this second study supported previous findings, showing significant DNA methylation changes in regions associated with the HIF and renin–angiotensin system (RAS) pathways [18]. The implication of the HIF pathway in these studies is not surprising as it contains many genes essential to regulating cellular oxygen delivery and therefore acclimatization at high altitude. Similarly, recent studies suggest the RAS pathway is modulated in response to hypoxia, upregulating angiotensin signaling, which in turn reinforces HIF pathway activation and oxidative stress, highlighting an interlinked response network between RAS and hypoxia-responsive genes [19, 20]. In this same study, pathway analysis results included terms related to glycolytic processes, hematopoiesis, and angiogenesis. Additionally, the study found a global trend of hypermethylation in individuals at high altitude. These findings raise important questions about how acute environmental exposures like hypoxia may modulate epigenetic marks in accessible tissues such as blood.

We expand on this work with an unbiased, high-throughput investigation of global DNA methylation levels before and during an acute but stable high-altitude exposure in a cohort of healthy sea-level residents. Importantly, our participant group reflects a representative cross-section of the general population, including individuals of varying fitness levels, BMIs, genders, and ethnicity. In addition, because our participants were transported to a single elevation and did not engage in strenuous physical activity prior to sampling, our study design reduces confounding effects associated with exercise-induced stress during ascent. Considering previous studies, we hypothesized that acute high-altitude exposure would result in global hypermethylation. To our knowledge, this is the first study to explore epigenome-wide DNA methylation changes in a general population cohort undergoing controlled high-altitude exposure without additional physical exertion.

## Results

### Genome-wide analysis and top differentially methylated positions

**Figure 1:**
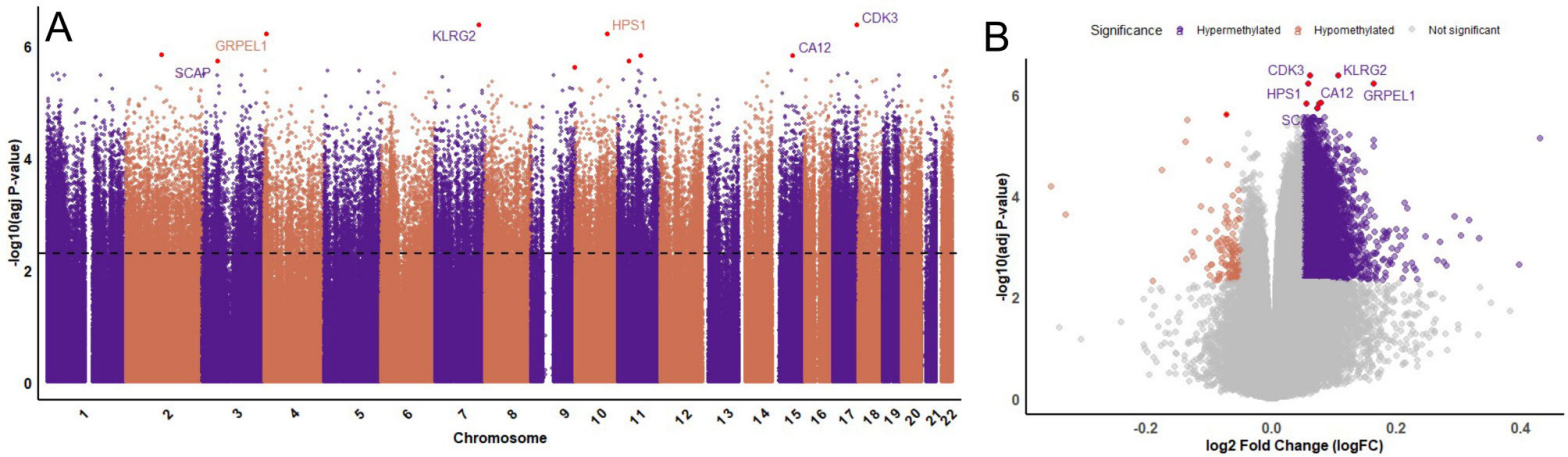
Genome-wide CpG sites. (A) Manhattan plot showing the significance and location of each CpG site across the genome, with the dashed line indicating a corrected *P*-value of .05 and highlighting the top 10 sites in red. Unlabeled red points do not have an associated gene. (B) Volcano plot with top genes also labeled. Sites on this plot were considered significant if they had an adjusted *P*-value < .05 and a log fold change over 0.05.

**Figure 2:**
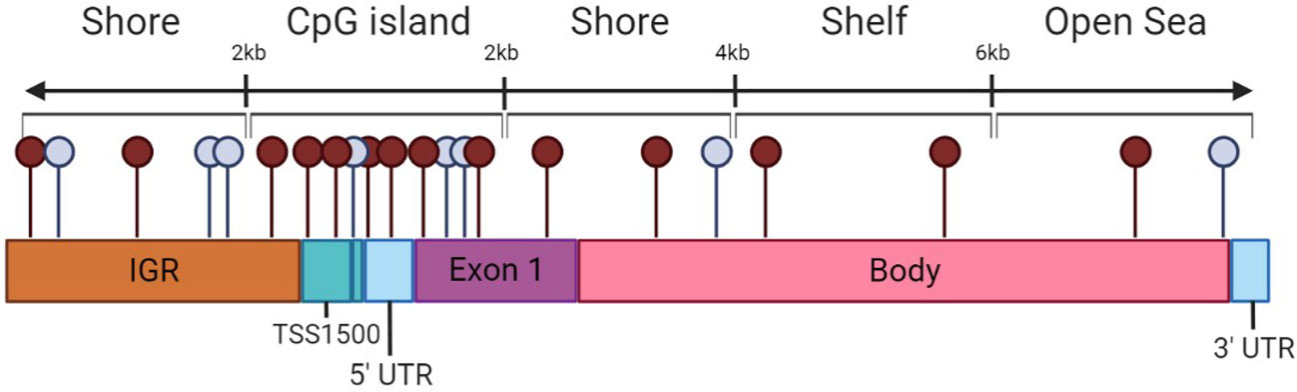
CpG region types and genomic features. This figure shows the types of locations a single CpG can occur in. The topmost text references the regions based on the density of the CpG sites; the densest area of CpGs being islands, moving further away from islands in either direction are shores, then shelves, and lastly open seas. These regions can be found on any genomic features such as the intergenomic region (IGR), TSS1500, TSS200, untranslated regions, exons, or the body of a gene.

We identified 58046 differentially methylated positions (DMPs), with 56084 positions showing increased methylation levels at high altitude and 1962 showing decreased methylation levels at high altitude. Figure 1 shows the locations and significance of each CpG site tested across the genome. The entire list of significant positions can be found in Supplemental Table 1. The locations of the DMPs were examined to determine their location within the genome. A CpG site is considered a “shore” if it is >2Kb from a CGI and a “shelf” if it is >2Kb but <4Kb from the island. Further still are sites that are part of an “open sea” [21]. CpG sites can further be described using genomic regions such as exons, 3′ or 5′ untranslated region (UTR), body, exon band, intergenic region (IGR), or transcriptional start sites (TSS1500 or TSS200). These locations can be seen visualized in Fig. 2. Interestingly, despite CGIs commonly being in promoter regions, it is the patterns of DNA methylation in CpG shores that are most associated with gene expression [22]. The distribution of significant DMPs can be seen categorized by CpG location in Table 1, and categorized by genomic feature in Table 2 along with the distribution of hyper- and hypomethylated sites across the genome. These tables list all DMPs with an adjusted *P*-value ≤ .005.

**Table 1:** DMP locations in relation to CpG regions.

Location	All sites *n* (% of total)	Increased at high altitude *n* (% of type)	Decreased at high altitude *n* (% of type)
Open seas	34 765 (59.9)	34 010 (97.8)	755 (2.2)
Shelf	4930 (8.5)	4841 (98.2)	89 (1.8)
Shore	12 553 (21.6)	12 330 (98.2)	223 (1.8)
Island	5798 (10.0)	4903 (84.6)	895 (15.4)
*Total*	*58 046*	*56 084 (96.6)*	*1962 (3.4)*

**Table 2:** DMP locations in relation to genomic features.

Feature	All sites *n* (% of total)	Increased at high altitude *n* (% of type)	Decreased at high altitude *n* (% of type)
1st Exon	1159 (2.0)	1036 (89.4)	123 (10.6)
3′ UTR	1554 (2.7)	1514 (97.4)	40 (2.6)
5′ UTR	4602 (7.9)	4392 (95.4)	210 (4.6)
Body	22 535 (38.8)	21 970 (97.5)	565 (2.5)
Exon band	400 (0.7)	392 (98.0)	8 (2.0)
IGR	17 633 (30.4)	17 251 (97.8)	382 (2.2)
TSS1500	7300 (12.6)	7043 (96.5)	257 (3.5)
TSS200	2863 (4.9)	2486 (86.8)	377 (13.2)
*Total*	*58 046*	*56 084 (96.6)*	*1962 (3.4)*

**Figure 3:**
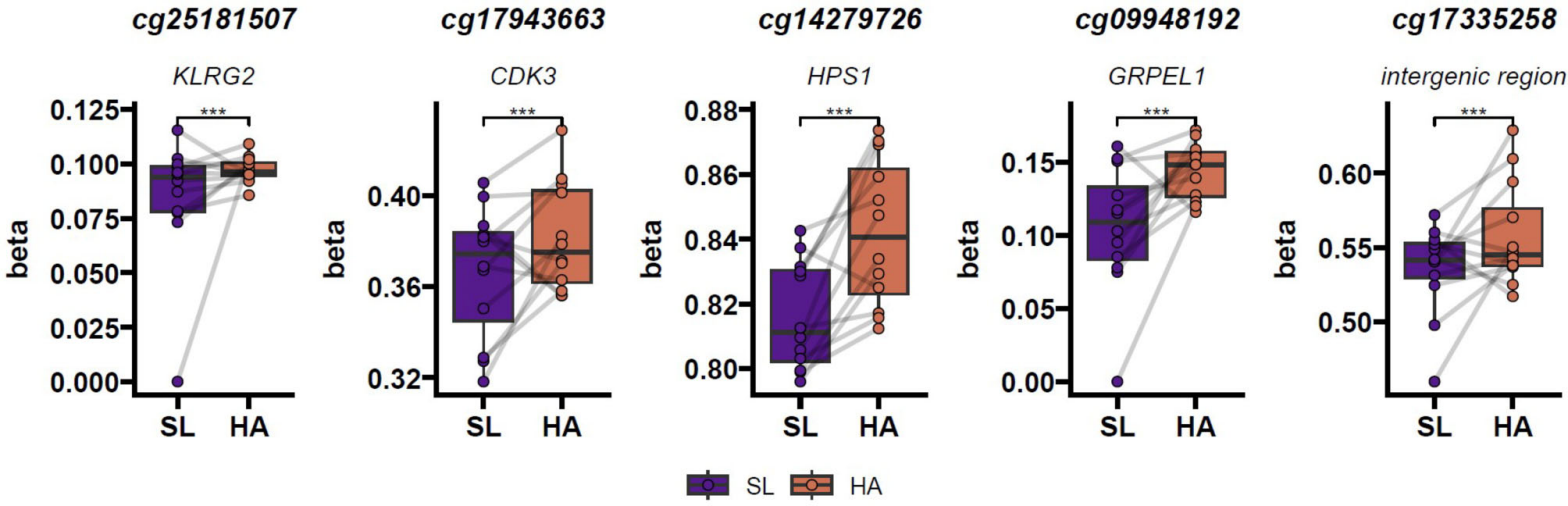
Top five most differentially methylated positions. These figures show the methylation at each of the top five most significant DMPs, using B values, at sea level and high altitude.

The top five DMPs, regardless of which region they are located in, are presented in Table 3. DMPs found in the IGR are not generally associated with specific genes. Changes at the top five DMPs between individuals at sea level and high altitude are shown in Fig. 3.

**Table 3:** Top five most differentially methylated CpG positions.

CG	Gene	Feature	Region	Sea level avg	High altitude avg	Log FC	Adj. *P* value
cg25181507	*KLRG2*	TSS1500	Island	0.043	0.149	0.107	4.13E–07
cg17943663	*CDK3*	3′ UTR	Shore	0.343	0.405	0.062	4.13E–07
cg14279726	*HPS1*	TSS1500	Open sea	0.799	0.858	0.058	6.12E–07
cg09948192	*GRPEL1*	TSS200	Island	0.052	0.216	0.164	6.12E–07
cg17335258		IGR	Open sea	0.508	0.586	0.079	1.42E–06

### Pathway analysis

All significant DMPs were analyzed in an overrepresentation analysis to reveal pathways associated with significant sites using Reactome. The top 10 most significantly enriched pathways are presented in Table 4. Top pathways are related to DNA damage/telomere stress-induced senescence, hypoxia-related cancer (AKT1 E17K and WNT pathway signaling), and calcium homeostasis. The full list of pathways can be found in Supplemental Table 2.

**Table 4:** Reactome pathways related to significantly differentially methylated CpG sites.

Reactome ID	Reactome pathway	Adj. *P* value
2559586	DNA damage/telomere stress-induced senescence	4.93633E–15
5674400	Constitutive signaling by AKT1 E17K in cancer	3.60462E–14
5693571	Nonhomologous end-joining (NHEJ)	4.49164E–14
2122948	Activated NOTCH1 transmits signal to the nucleus	4.49164E–14
114508	Effects of PIP2 hydrolysis	9.59328E–14
4791275	Signaling by WNT in cancer	1.48672E–13
212676	Dopamine neurotransmitter release cycle	1.48672E–13
418360	Platelet calcium homeostasis	1.71614E–13
8941326	RUNX2 regulates bone development	3.68985E–13
380972	Energy dependent regulation of mTOR by LKB1-AMPK	3.68985E–13

### Differentially methylated regions

**Figure 4:**
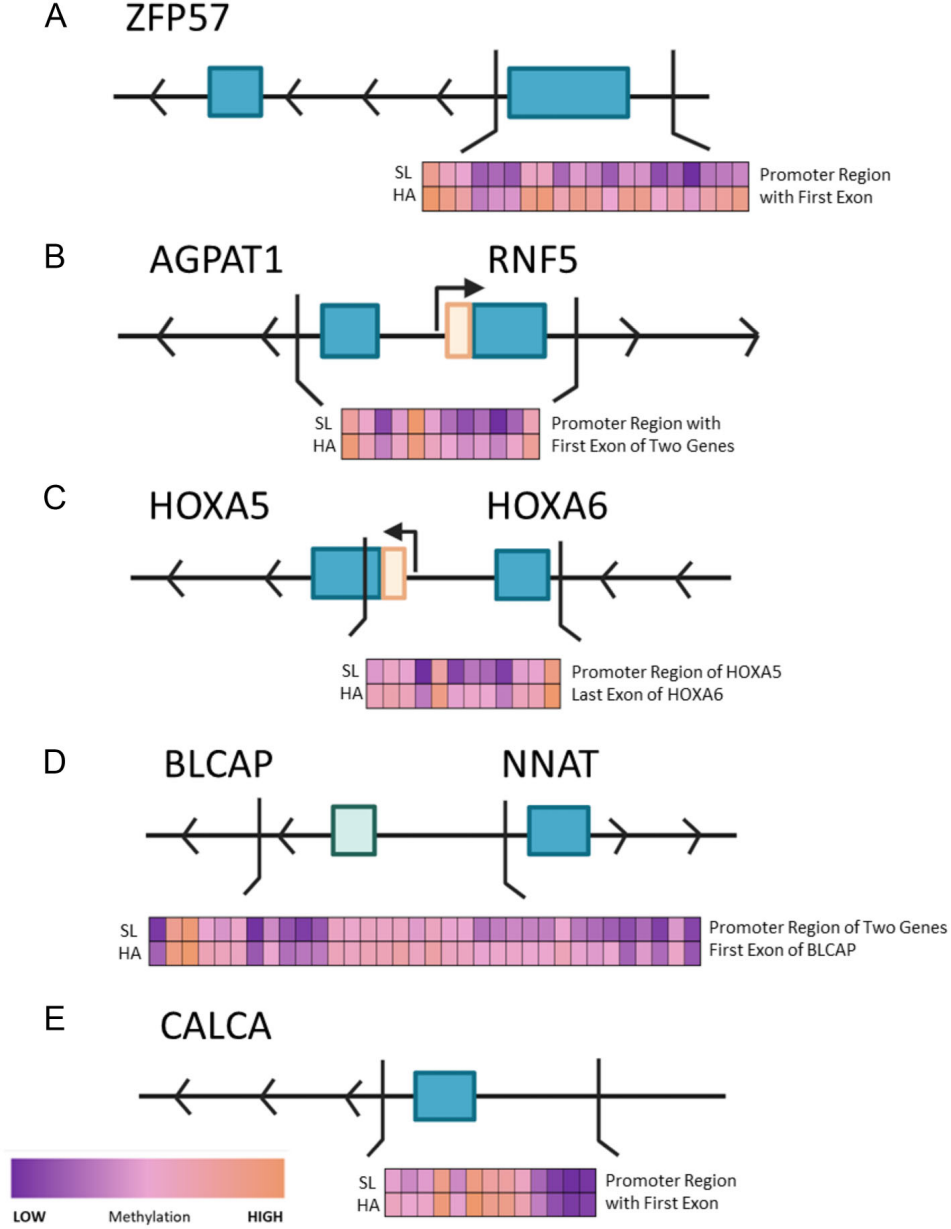
Top five most differentially methylated regions. These figures show the methylation at each of the top five most significant DMRs, using B values, at sea level and high altitude. These figures also show where the DMRs are located in relation to the nearest gene: (A) a DMR in the promoter region of *ZFP57*, (B) a DMR which includes the promoter regions of both *AGPAT1I* and *RNF5*, (C) a DMR in the promoter of *HOXA5*, (D) a DMR in the promoter regions on *BLCAP* and *NNAT*, and finally (E) a DMR in the promoter region of *CALCA*.

In addition to individual sites, we analyzed differentially methylated regions (DMRs). These DMRs contain multiple significant DMPs close together and thus indicate a pattern of change over a large region, which can be more indicative of a resulting change in gene expression. There were 19 DMRs with a corrected *P* value < .005. The top five regions are listed in Table 5 and all significant DMPs within the top five regions are visualized in Fig. 4. All significant DMRs can be found in Supplemental Table 3. Many of these sites had links with calcium processes, the others with zinc finger proteins, glucose processes, and erythropoiesis. The top DMRs show increased levels of methylation on average at high altitude as compared to sea level.

**Table 5:** Top five most differentially methylation regions.

Gene	CHR	Start	End	Width	Adj. *P* value	Feature	Region
*ZFP57*	6	29 648 161	29 649 092	931	2.79E–06	TSS1500	Open sea
*AGPAT1, RNF5*	6	32 144 978	32 146 779	1801	7.07E–06	TSS1500	Open sea
*HOXA5, HOXA6*	7	27 183 133	27 185 732	2599	1.41E–05	TSS1500	Island
*BLCAP, NNAT*	20	36 148 133	36 149 656	1523	1.41E–05	5'UTR	Island
*CALCA*	11	14 993 378	14 994 989	1611	4.24E–05	TSS1500	Shore

The first DMR shown in Fig. 4A is a region with 931 base pairs containing 24 significant DMPs in an open sea/intergenomic region. This region codes for *ZFP57* and is known to be a transcriptional repressor due to its role in facilitating DNA methylation [23–25]. The second DMR (Fig. 4B) is 1801 base pairs containing 35 DMPs, which overlap the transcription start sites of both *AGPAT1*, a phospholipid-synthesizing enzyme, and the ring finger ubiquitin ligase or *RNF5* gene. The third DMR (Fig. 4C) is a 2599-base pair region containing 41 DMPs found in the promoter region of *HOXA5*, a gene connected to lung development and the respiratory system.

The fourth and fifth DMRs (Fig. 4D and E) are found within genes relating to calcium or calcium processes. DMR 4, which is comprised of 1523 base pairs and contains 39 DMPs, is located in a region which overlaps with the 5′ UTR of *BLCAP*, a tumor suppressor, and neuronatin or *NNAT. NNAT* is involved in the regulation of ion channels in the brain during development and guides pluripotent stem cells into differentiation by facilitating an increase in calcium. DMR 5 is found in the promoter region of *CALCA* or calcitonin, a hormone that works to decrease calcium levels in the blood and inhibits bone reabsorption. It is a 1611-base pair region with 28 DMPs.

### Comparative analysis

To identify commonalities between our data and other similar studies, we compared our findings to that of Childebayeva *et al*. (2019) [17], who found significant changes in the DNA methylation levels within genes related to the HIF pathway during a 10-day trek to 5160 m elevation. By the seventh day, they found significant increases in DNA methylation levels in select regions of the genes *PPARA* and *EPAS1*. We also found significant increases in methylation at several sites within these genes. We identified 1 DMP within the *PPARA* gene. The *EPAS1* region included 4 significant DMPs, all located in the gene body. In contrast, Childebayeva reported decreases in RXRA methylation, while we found 14 significant DMPs in our dataset showing increased methylation levels, with 9 in the gene body. Childebayeva also reported decreases in *EPO* methylation; however, we found no significant DMPs. Notably, the methods utilized in these two studies differ (bisulfite sequencing versus a chip-based scan) and thus must be interpreted cautiously.

**Figure 5:**
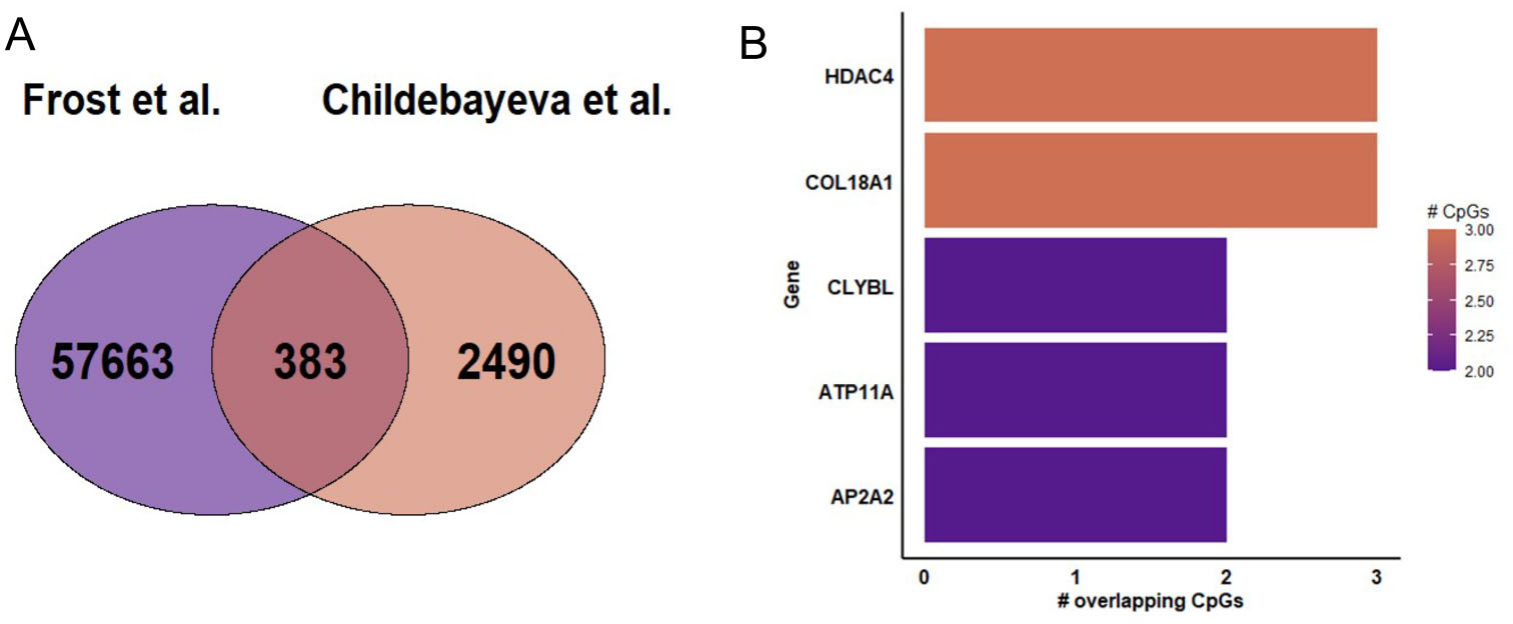
Comparing to previous findings. (A) A Venn diagram of significant DMPs from our study compared to Childebayeva *et al*. [18], with an overlap of 383 DMPs. (B) The genes listed by the number of DMPs per gene that were found in the compared overlapping DMPs.

However, in a subsequent study, Childebayeva *et al*. [18] utilized the same MethylationEPIC technology as we report here, albeit with a different tissue type, saliva, and using a different pipeline. Comparing these results resulted in an overlap of 383 CpG sites across studies (Fig. 5A). The genes with the most overlapping DMPs were *HDAC4* and *COL18A1*, which contained three DMPs found significant in both datasets. These genes were followed by *CLYBL, ATP11A*, and *AP2A2*, which each contained two DMPs, which can be seen in Fig. 5B. Additionally, all overlapped genes and DMPs can be found in Supplemental  Table 4.

## Discussion

This study aimed to analyze the difference in methylation levels throughout the genome before and during an acute but stable high-altitude exposure in a cohort of healthy sea-level residents. Our results showed that 96.6% of significantly DMPs resulted in increased DNA methylation after one night at high altitude, which supports prior findings that hypoxia results in a state of genome-wide hypermethylation [26–28]. Several mechanisms may explain the widespread hypermethylation observed following high-altitude exposure. One key factor may be the oxygen dependency of ten-eleven translocation (TET) enzymes, which mediate active DNA demethylation. Reduced oxygen availability impairs TET activity, resulting in passive accumulation of methylation marks [26, 29]. HIFs have also been shown to transcriptionally upregulate DNMTs, further enhancing methylation [30]. These changes may represent an adaptive epigenetic response, selectively silencing genes involved in apoptosis, inflammation, and metabolic regulation to preserve cellular function under stress. Oxidative stress, a hallmark of hypoxia, may further promote methylation through chromatin remodeling and DNA damage pathways [31]. Collectively, these processes suggest that DNA hypermethylation under hypoxic conditions may be driven by both direct biochemical limitations and active cellular adaptation.

Interestingly, positions found in CGI sites did not on average have many changes and those that did change became less methylated after acute high-altitude exposure. This supports previous research showing that CpG sites within CGIs tend to be hypomethylated regardless of gene expression levels. It is thought that CGIs may be regulated by other means such as histone modifications or polycomb repression [9, 32, 33].

While the majority of the significant DMPs were found in the body of genes (38.8%) and in intergenomic regions unassociated with specific genes (30.4%), these sites are less correlated with gene expression. However, they remain highly regulated as they contain regulatory elements that affect other target genes and likely play a role in protecting DNA from mutagenic damage [9, 34, 35]. On the other hand, many of the DMRs were found in promoter regions and it is likely these changed regions have a larger impact on subsequent expression than a changed position.

### Wnt and Notch signaling

The topmost DMP is associated with Killer Cell Lectin Like Receptor G2 (*KLRG2*), which is expressed mainly in the kidney as an integral part of the membrane enabling carbohydrate binding and is also used as a marker for lung cancer [36]. This gene also showed significantly reduced gene expression (adj. *P* value = .0035) in the same peripheral blood cells in a previous study by our group [37]. *KLRG2* has also been shown to have crosstalk with the Notch [38] and Wnt pathways, which were significant pathways overrepresented in our analysis [39].

In fact, our data demonstrate many significantly enriched pathways related to Notch, a pathway that is highly conserved in metazoans and plays a major role in cell fate determination, proliferation, and apoptosis. In hypoxia, HIF-1α aids in stabilizing the Notch intracellular domain, resulting in increased expression of downstream Notch targets, increasing cell proliferation and protecting against apoptosis [40, 41]. In addition, Notch signaling pathway genes are under genetic selection in high-altitude native populations of humans and other animals such as chickens, yaks, and pigs living on the Tibetan plateau [42]. While the exact mechanism conferring hypoxia resistance is unknown, there are indications that it could be tied to erythropoiesis, angiogenesis, vascular tone, and cellular metabolism pathways [42].

Further, both the Wnt and Notch pathways, through *hairy* activation, have been linked to increased hypoxia tolerance in *Drosophila* exposed to hypoxia over many generations allowing them to survive in otherwise lethally low oxygen levels [43, 44]. Wnt also has considerable crosstalk with the HIF pathway, with WNT being affected by or influencing all of HIFα’s three subunits [45–47]. The WNT pathway has an effect on a variety of systems including neuronal differentiation of glioblastoma stem cells [45], epithelial–mesenchymal transitions [48], and TRPC5 channels, which are activated by elevated levels of calcium [49].

### Calcium handling

Calcium homeostasis was revealed as a significantly impacted process. At high altitude there are many reported changes to calcium metabolism such as increases in calcium sequestration, decreased bone mineral density, and permeability changes of the cell membrane, affecting both K^+^ and Ca^2+^ ion channels and resulting in increases in intracellular calcium [50]. Thus, DNA methylation may assist in regulating expression of calcium handling-related genes to help modulate or counteract these changes to maintain cellular calcium homeostasis. Along with pathways we also saw DMPs and DMRs associated with calcium processes. For example, a DMP connected with *PIP2*, which is activated via the HIF-1α pathway, is important for calcium sequestration [51, 52]. Another DMP associated with *RUNX2* is a transcription factor, which acts as a “master switch” of the development and maintenance of bone, teeth, and cartilage [53].

DMRs 4 and 5 are located in the promoter regions of *NNAT* and *CALCA*, respectively. Each of these genes also plays important roles in calcium homeostasis. *NNAT* expression is mediated by oxidative stress and is shown to lead to neuronal differentiation through the use of calcium-mediated channels, regulating Ca^2+^ influx [54]. Furthermore the decreased expression of *NNAT* leads to an increase in cytoplasmic Ca^2+^ levels, which may be contributing to the increases we see at high altitude [55]. Prior work by Childebayeva *et al*. also shows increases in DNA methylation in the same DMRs associated with *NNAT* in Andean individuals born at high altitude as compared to Andean individuals with similar ancestry born and living at low altitude, indicating that these sites are an important part of adaptation to high altitude both in acute and long-term exposures [56].

### Carbonic anhydrase and metabolism

Interestingly, one of the top DMPs is associated with *CA12* (carbonic anhydrase 12), which is responsible for encoding for an isoform of carbonic anhydrase. These are a family of enzymes, which convert carbon dioxide and water into carbonic acid, and bicarbonate. This process is especially highlighted at high altitude as the increase in minute ventilation drives a shift toward respiratory alkalosis, which is mitigated by carbonic anhydrase activity. These enzymes can also play a role in respiration, calcification and bone reabsorption, and the formation of cerebrospinal fluid [57–59].

### DNA damage

Some of the topmost significant Reactome pathway results fall into categories of DNA damage and repair pathways including “DNA damage/telomere stress-induced senescence” and “nonhomologous end-joining (NHEJ).” The first term refers to pathways which activate in response to reactive oxygen species (ROS) or environmental stress; both cause double-strand breaks in the DNA [60]. ROS have been shown to increase in hypoxic conditions, as insufficient oxygen causes the electron transport chain in mitochondria to misfunction and cause an accumulation of ROS [61]. NHEJ is then activated in turn in response to the double-strand breaks, which activates multiple checkpoints and repair proteins. Unfortunately, studies have shown that many genes in this pathway are downregulated in hypoxia leading to altered DNA repair patterns [62, 63]. Similarly, hypoxia can lead to alternative splicing, which leads to dysfunctional histone deacetylases (HDACs), another significant term in the pathway analysis, which also leads to impaired double-strand break repair [64].

As a final note, *ZFP57* and *RNF5*, significant DMRs, come up together in gene sets associated with rheumatoid arthritis, musculoskeletal system disease, and bone disease, found in DISEASES Experimental Gene-Disease Association Evidence Scores [65]. They have also been shown to have DMRs in disease states such as Parkinson’s and Alzheimer’s, which could play a role in the cognitive changes at high altitude [66, 67].

### Comparing to previous findings

The identification of a shared set of genes across two independent studies points toward a non-random, biologically meaningful convergence that may reflect stable epigenetic responses to cellular stress or environmental challenge. Functionally, the list is enriched for epigenetic regulators, signaling scaffolds, metabolic enzymes, and structural/neuronal genes, a mixture that maps well onto canonical hypoxia responses. *HDAC4* with three overlapping DMPs is a class IIa histone deacetylase known to regulate chromatin accessibility and transcription. It is also a known regulator of HIF-1α, serving as a co-regulator that can promote or suppress HIF-1α activity depending on context. Through this, *HDAC4* influences the expression of genes involved in glycolysis, angiogenesis, and cell survival [68–71]. The appearance of *HDAC4* with genes such as *SND1*, a regulator of mRNA stability and processing, and *CAMTA1*, a transcription factor linked to calcium-responsive pathways suggests a broader epigenetic landscape responsive to changes in oxygen availability, calcium signaling, and oxidative stress, which links further to the pathways and genes found in the analysis of our dataset alone. Notably, other epigenetic regulators such as *DNMT3L, EZH1, MBD2/MBD3, SMARCE1, SETD4*, and *SMYD3* also emerge in this dataset. Their altered methylation patterns further implicate epigenetic mechanisms such as DNA methylation, histone modifications, and chromatin restructuring in the adaptation to hypoxic environments. Taken together, the recurrence of these genes across studies, particularly those involved in transcriptional regulation, chromatin remodeling, intracellular signaling, and neuronal structure, suggests a concerted epigenetic response potentially tied to hypoxia adaptation, neuroplasticity, or stress resilience.

## Conclusion

In conclusion, these findings provide insight into how short-term environmental hypoxia may influence the human epigenome, highlighting DNA methylation as a dynamic marker of environmental exposure. Exposure to high-altitude hypoxia resulted in genome-wide hypermethylation, which is supported by much of the current literature. Additionally, many of the genes and pathways related to significant DMPs show connections to processes already established to be affected by hypoxia. Though some of these top DMPs were associated with genes, promotors, or CGIs, many of these CpG sites are not in the regions directly associated with gene expression levels, demonstrating a need to better understand the role CpG sites located in open sea and body regions play in gene expression. In addition, the interplay between DNA methylation and other epigenetic mechanisms such as ncRNAs and histone modifications needs to be better understood to understand the changes happening on a larger scale, and the role the combination of these changes has on our phenotypic plasticity at high altitudes.

## Materials and Methods

### Ethical approval

This study was approved by the University of California, Riverside Clinical Institutional Review Board (HS 19-076). All participants were informed of the study’s purpose and risks. Participants provided written informed consent in their native language (English). The work was conducted in accordance with the Declaration of Helsinki, except for registration in a database.

### Participants and study design

Twelve participants (9 men, 3 women) currently residing at sea level were recruited for this study. Participants were between 19 and 32 years of age (25 ± 4.5 years) with no known history of major cardiovascular or pulmonary disease. The study excluded individuals with a history of smoking (cigarettes, e-cigarettes, marijuana), current pregnancy, or travel to elevations above 2500 m within 1 month of the initial measurements to avoid impacts of previous high-altitude acclimatization on outcomes. In accordance with these criteria, all study participants reported no high altitude travel within the previous 3 months, exceeding the lifespan of circulating neutrophils, monocytes, and most lymphocytes [72, 73, 74].

During the study, participants abstained from taking acetazolamide or anti-inflammatory medications, such as ibuprofen, which may interfere with ventilatory acclimatization to high altitude [75]. Participants were transported from UC Riverside (340 m) to Barcroft Station (3800 m) (White Mountain Research Center) in vans and underwent a gradual ascent from 340 to 1216 m over a period of 4 h, followed by an ascent from 1216 m to the final elevation of 3800 m in 2 h where they stayed for 3 days. Sea-level measures were conducted during fasting in the early morning at UC Riverside, while high-altitude measures were taken during fasting on the first morning after sleeping one night at Barcroft Station.

### Sample collection and preparation

Fasting veinous blood samples were obtained from participants both at sea level and high altitude in the early morning. Blood was collected by a licensed phlebotomist or physician using standard venipuncture procedures; 10 ml of blood was collected in EDTA treated Vacutainer (BD, Franklin Laked, NJ, USA) tubes and kept at room temperature until processing within 1 h of collection.

DNA was isolated from buffy coat of fresh whole blood samples using the Gentra Puregene Blood Kit (Qiagen, Germantown, MD, USA) according to the manufacturer’s protocol for whole blood. DNA samples were immediately stored at −80°C for samples collected at sea level, or temporarily in liquid nitrogen at high altitude for transport to UC Riverside and subsequent storage at −80°C. DNA concentration and purity was verified via Nanodrop 2000 (Thermo Scientific, Waltham, MA, USA).

According to unmodified manufacturer’s protocol, 400 ng of genomic DNA underwent a bisulfite conversion treatment using EZ DNA Methylation Kit). Samples were then processed for methylation analysis on the Illumina Infinium MethylationEPIC BeadChip following the manufacturer’s protocol (Infinium HD Methylation Assay manual workflow) (Illumina, San Diego, CA, USA). Briefly, bisulfite-converted DNA was amplified, fragmented, and hybridized to the BeadChip. Following a wash step to remove unhybridized DNA, primer extension and staining was performed. BeadChips were imaged with an Illumina iScan.

### Data analysis

We used the well-defined *ChAMP* [76] pipeline, in which raw idat files were first read and assessed for quality. Samples that failed any quality control tests were removed, including probes with detection *P*-value > .01 (2245) and probes with less than three beads in at least 5% of samples per probe (2260). In addition, all probes that were non-CpG probes (2984), all SNP-related probes (97 578), all multi-hit probes (11), and all probes located in X and Y chromosomes were also removed (16 773). After quality control filtering and probe exclusion, 744 067 high-quality sites remained. Data were then normalized using BMIQ (Beta-Mixture Quantile Normalization) [77]. This workflow then used *ComBat* [78] to correct any batch corrections encountered by using multiple BeadChip arrays using an empirical Bayes method.

Resulting DNA methylation levels are shown as β-values, which represent the percent methylated using the following equation: $\beta = \frac{{( M ){\mathrm{\,\,}}\textit{Methylated}{\mathrm{\,\,}}\textit{signal}{\mathrm{\,\,}}\textit{instensity}}}{{(U){\mathrm{\,\,}}\textit{Unmethylated}{\mathrm{\,\,}}\textit{signal}{\mathrm{\,\,}}\textit{intensity}{\mathrm{\,\,}} + {\mathrm{\,\,}}M}}$. These β-values were used to find DMPs using R package Limma [79] and DMRs using BumpHunter [80], both as part of the ChAMP package in R. After these processes, a total of 192 651 DMPs were found to be significant with a Benjamini-Hochberg (BH) adjusted *P*-value < .05. To further reduce false positives, we used a more strict cutoff of adjusted *P*-value < .005, leaving us with 58 046 significant DMPs and 19 significant DMRs. Finally, a Reactome pathway Gene Set Enrichment Analysis was conducted through MethylR [81] to return significant pathways with an adjusted *P* value of <.05 (BH).

Data are presented throughout the manuscript as mean (standard deviation). Asterisks indicate significant differences at *P* < .05 (*), *P* < .01 (**), *P* < .001 (***), or *P* < .0001(****).

## Supplementary Material

dvag004_Supplemental_Files

## Data Availability

The datasets used and/or analyzed during the current study are available from the corresponding author on reasonable request.
