# Supplementary material for: Genome-wide DNA methylation changes after 24 hours at high altitude
Source: Environ Epigenet. 2026 Feb 9;12(1):dvag004. doi: 10.1093/eep/dvag004 (PMC12951794; doi:10.1093/eep/dvag004)
Supplement: dvag004_Supplemental_Files [file dvag004_supplemental_files.zip › Figures_for_EPIC_Resubmit.pptx]

## Slide 1
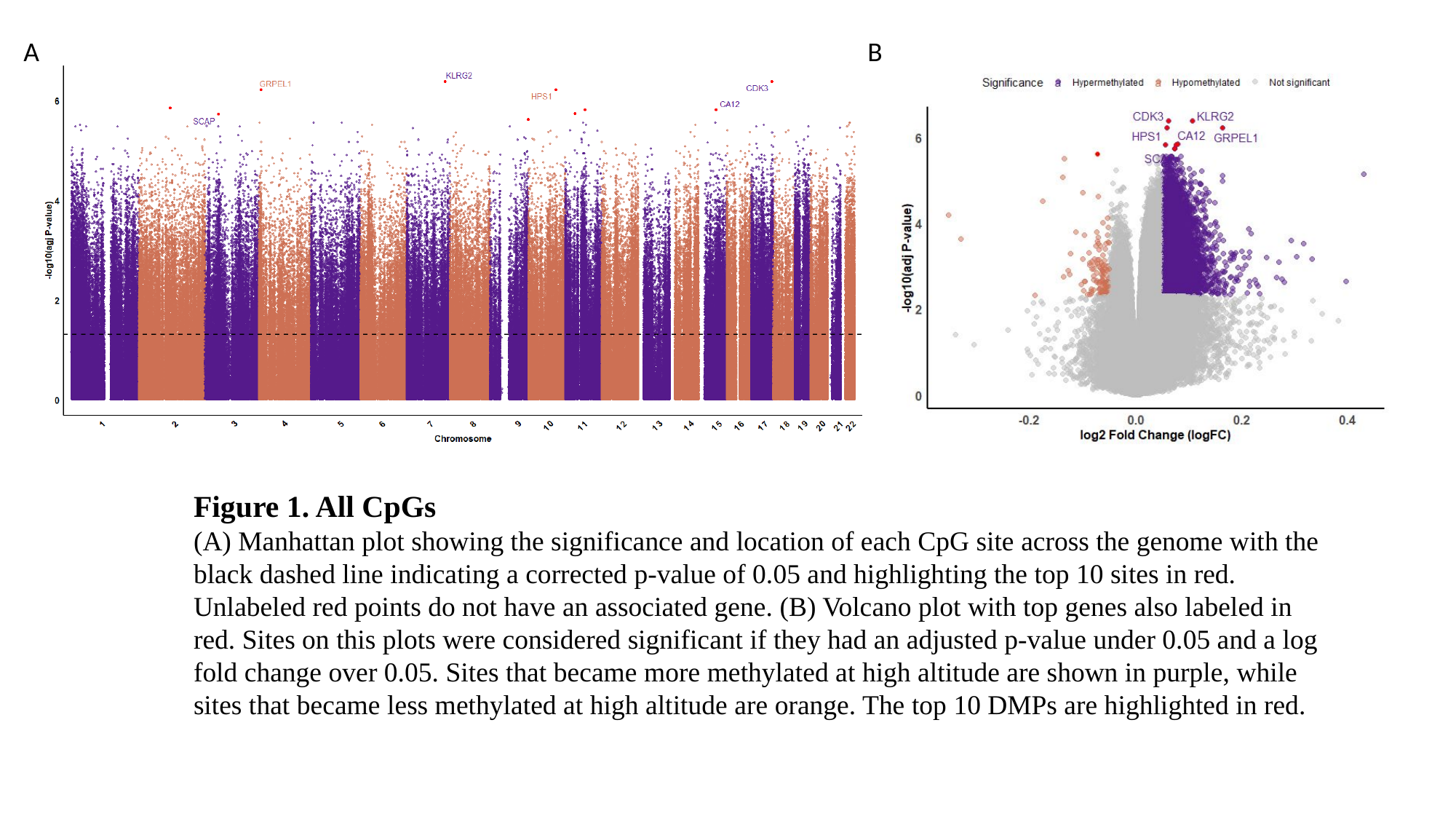

A							 B
Figure 1. All CpGs
(A) Manhattan plot showing the significance and location of each CpG site across the genome with the black dashed line indicating a corrected p-value of 0.05 and highlighting the top 10 sites in red. Unlabeled red points do not have an associated gene. (B) Volcano plot with top genes also labeled in red. Sites on this plots were considered significant if they had an adjusted p-value under 0.05 and a log fold change over 0.05. Sites that became more methylated at high altitude are shown in purple, while sites that became less methylated at high altitude are orange. The top 10 DMPs are highlighted in red.

## Slide 2
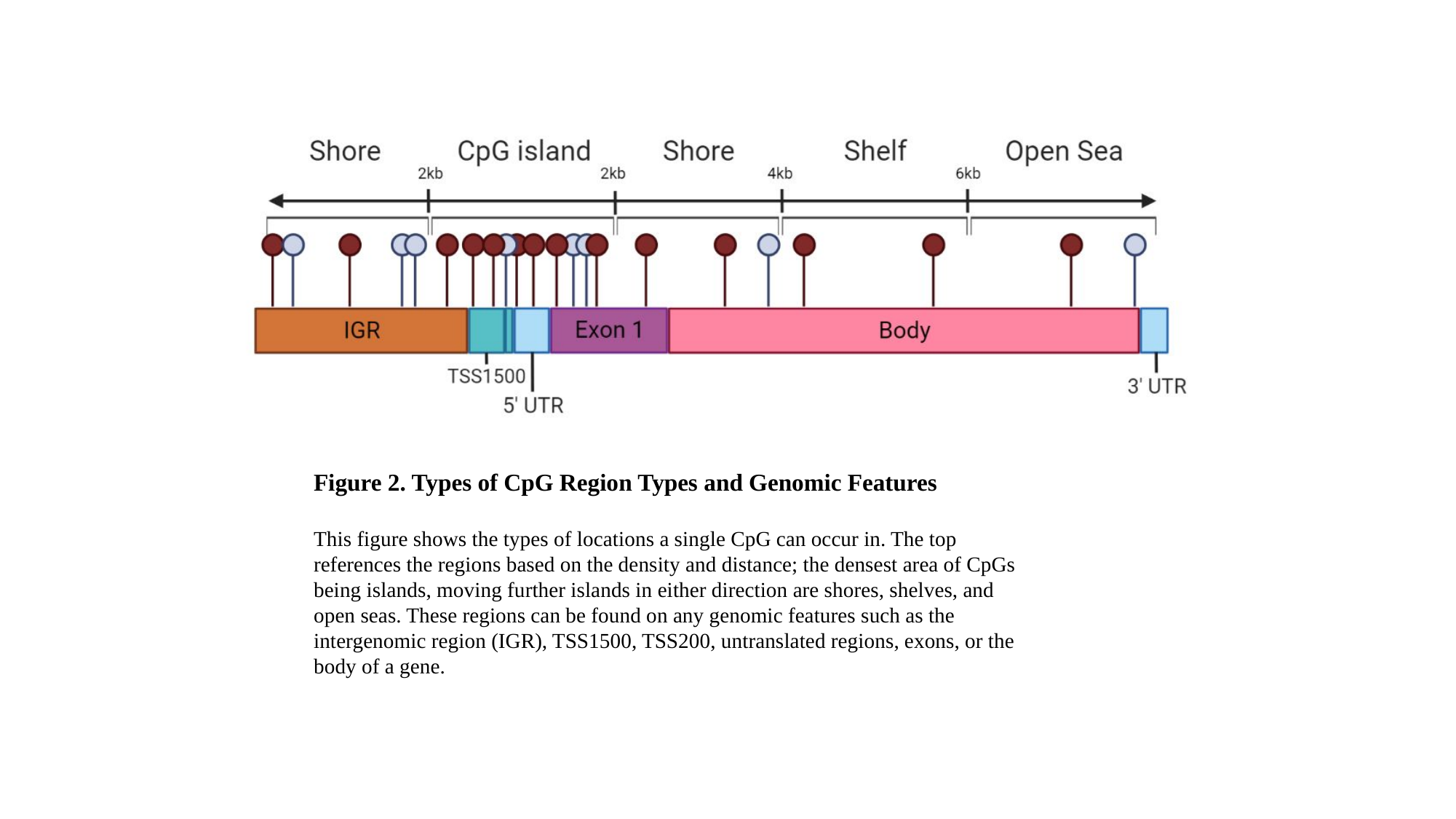

Figure 2. Types of CpG Region Types and Genomic Features
This figure shows the types of locations a single CpG can occur in. The top references the regions based on the density and distance; the densest area of CpGs being islands, moving further islands in either direction are shores, shelves, and open seas. These regions can be found on any genomic features such as the intergenomic region (IGR), TSS1500, TSS200, untranslated regions, exons, or the body of a gene.

## Slide 3
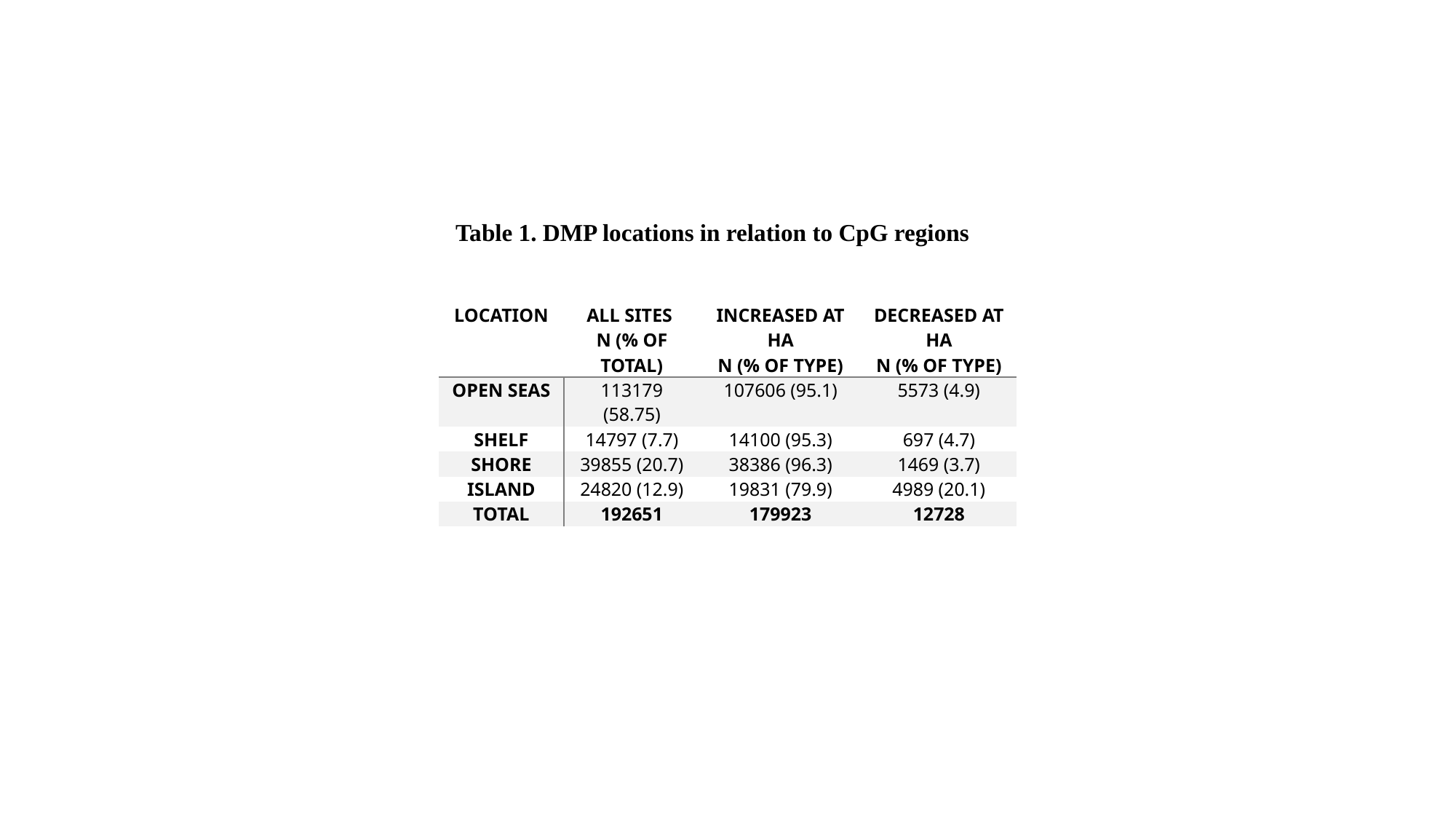

Table 1. DMP locations in relation to CpG regions
| LOCATION | All sites n (% of total) | increased at hA n (% of Type) | decreased AT HA n (% of Type) |
| --- | --- | --- | --- |
| Open Seas | 113179 (58.75) | 107606 (95.1) | 5573 (4.9) |
| Shelf | 14797 (7.7) | 14100 (95.3) | 697 (4.7) |
| Shore | 39855 (20.7) | 38386 (96.3) | 1469 (3.7) |
| Island | 24820 (12.9) | 19831 (79.9) | 4989 (20.1) |
| Total | 192651 | 179923 | 12728 |

## Slide 4
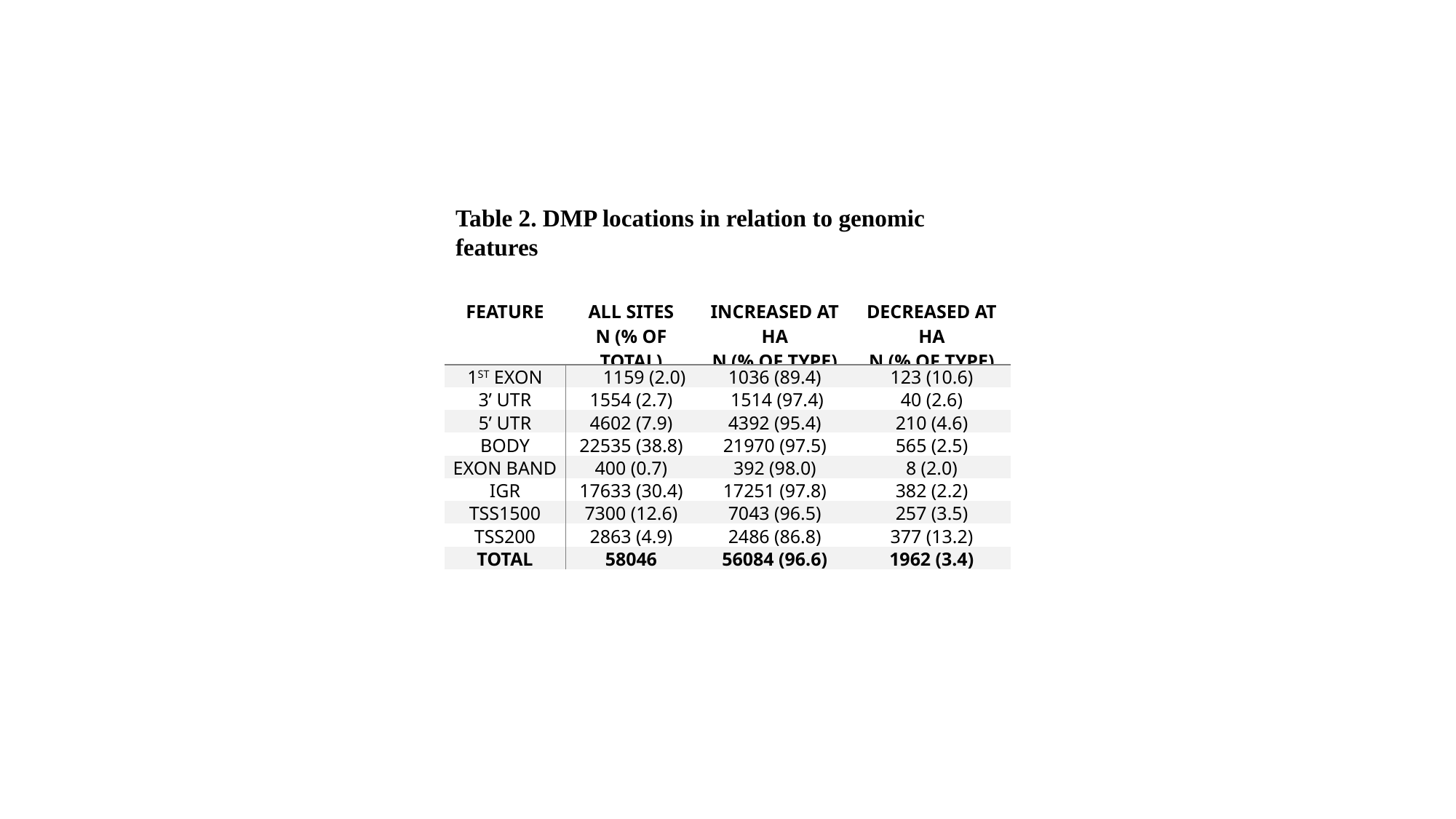

Table 2. DMP locations in relation to genomic features
| FEATURE | All sites n (% of total) | increased at hA n (% of Type) | decreased AT HA n (% of Type) |
| --- | --- | --- | --- |
| 1st EXON | 1159 (2.0) | 1036 (89.4) | 123 (10.6) |
| 3’ utr | 1554 (2.7) | 1514 (97.4) | 40 (2.6) |
| 5’ UTR | 4602 (7.9) | 4392 (95.4) | 210 (4.6) |
| BODY | 22535 (38.8) | 21970 (97.5) | 565 (2.5) |
| EXON baND | 400 (0.7) | 392 (98.0) | 8 (2.0) |
| IGR | 17633 (30.4) | 17251 (97.8) | 382 (2.2) |
| TSS1500 | 7300 (12.6) | 7043 (96.5) | 257 (3.5) |
| TSS200 | 2863 (4.9) | 2486 (86.8) | 377 (13.2) |
| Total | 58046 | 56084 (96.6) | 1962 (3.4) |

## Slide 5
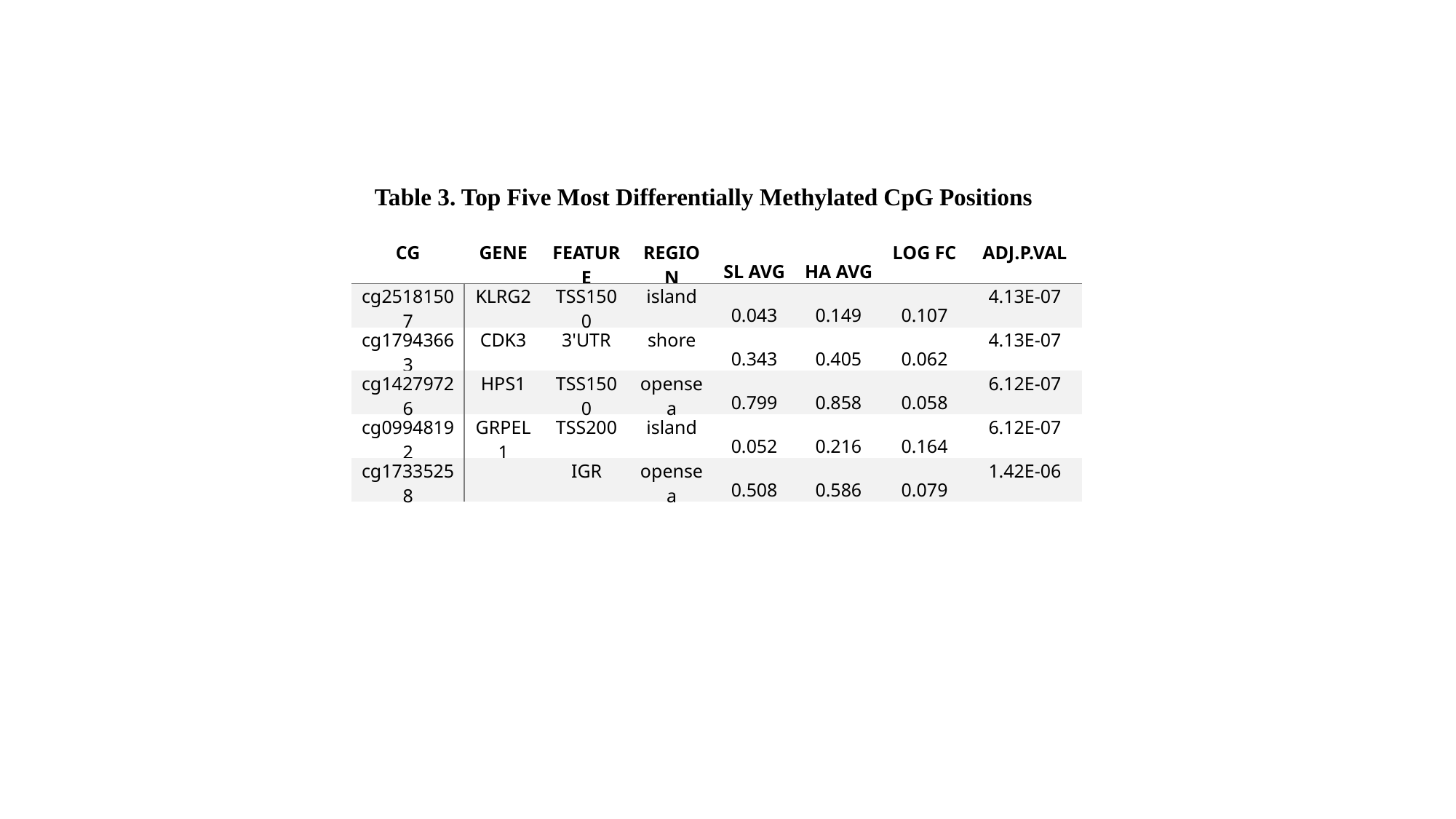

Table 3. Top Five Most Differentially Methylated CpG Positions
| cg | gene | feature | region | SL AVG | HA AVG | log fc | adj.P.Val |
| --- | --- | --- | --- | --- | --- | --- | --- |
| cg25181507 | KLRG2 | TSS1500 | island | 0.043 | 0.149 | 0.107 | 4.13E-07 |
| cg17943663 | CDK3 | 3'UTR | shore | 0.343 | 0.405 | 0.062 | 4.13E-07 |
| cg14279726 | HPS1 | TSS1500 | opensea | 0.799 | 0.858 | 0.058 | 6.12E-07 |
| cg09948192 | GRPEL1 | TSS200 | island | 0.052 | 0.216 | 0.164 | 6.12E-07 |
| cg17335258 | | IGR | opensea | 0.508 | 0.586 | 0.079 | 1.42E-06 |

## Slide 6
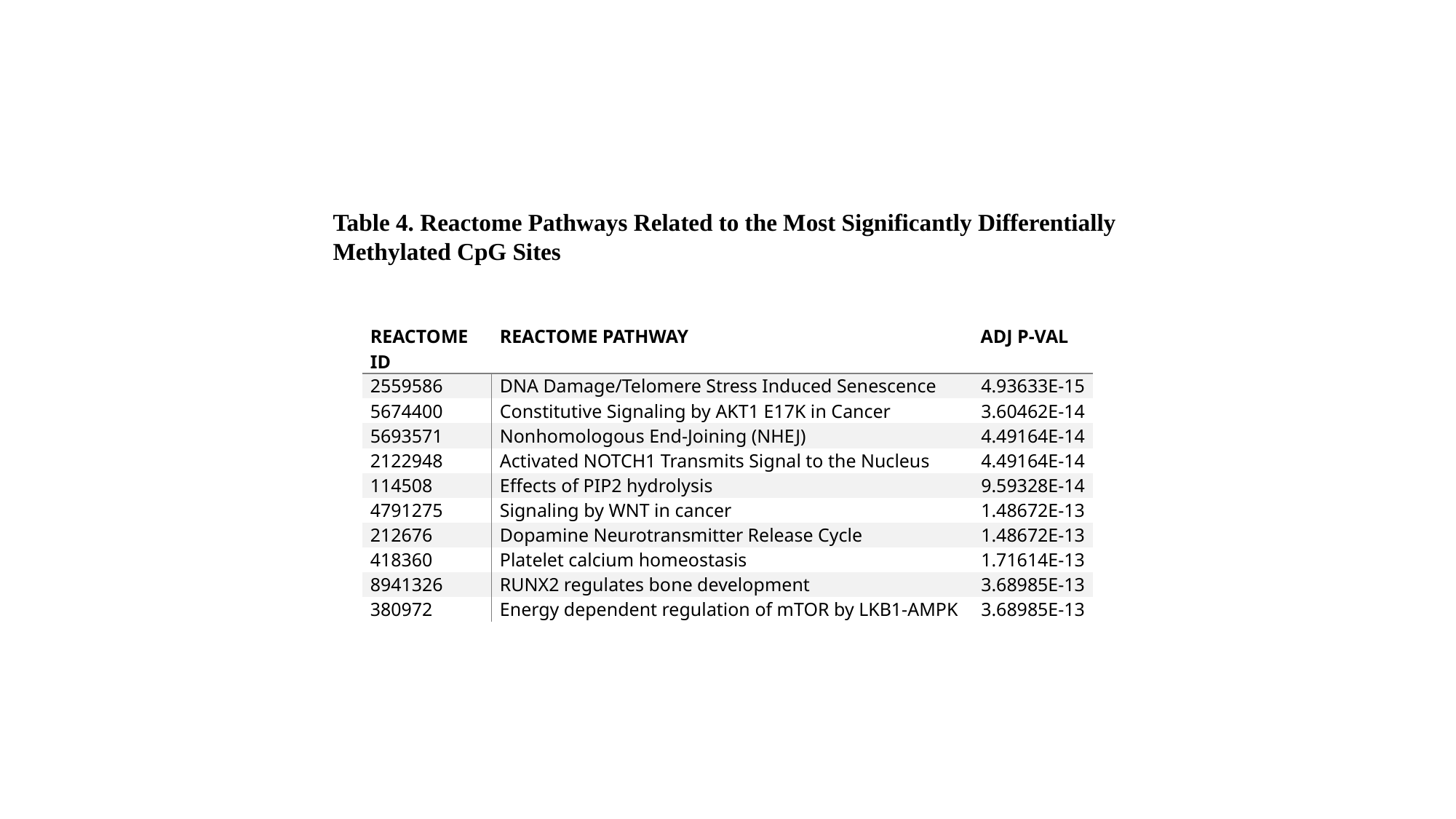

Table 4. Reactome Pathways Related to the Most Significantly Differentially Methylated CpG Sites
| REACTOME ID | REACTOME PATHWAY | ADJ P-VAL |
| --- | --- | --- |
| 2559586 | DNA Damage/Telomere Stress Induced Senescence | 4.93633E-15 |
| 5674400 | Constitutive Signaling by AKT1 E17K in Cancer | 3.60462E-14 |
| 5693571 | Nonhomologous End-Joining (NHEJ) | 4.49164E-14 |
| 2122948 | Activated NOTCH1 Transmits Signal to the Nucleus | 4.49164E-14 |
| 114508 | Effects of PIP2 hydrolysis | 9.59328E-14 |
| 4791275 | Signaling by WNT in cancer | 1.48672E-13 |
| 212676 | Dopamine Neurotransmitter Release Cycle | 1.48672E-13 |
| 418360 | Platelet calcium homeostasis | 1.71614E-13 |
| 8941326 | RUNX2 regulates bone development | 3.68985E-13 |
| 380972 | Energy dependent regulation of mTOR by LKB1-AMPK | 3.68985E-13 |

## Slide 7
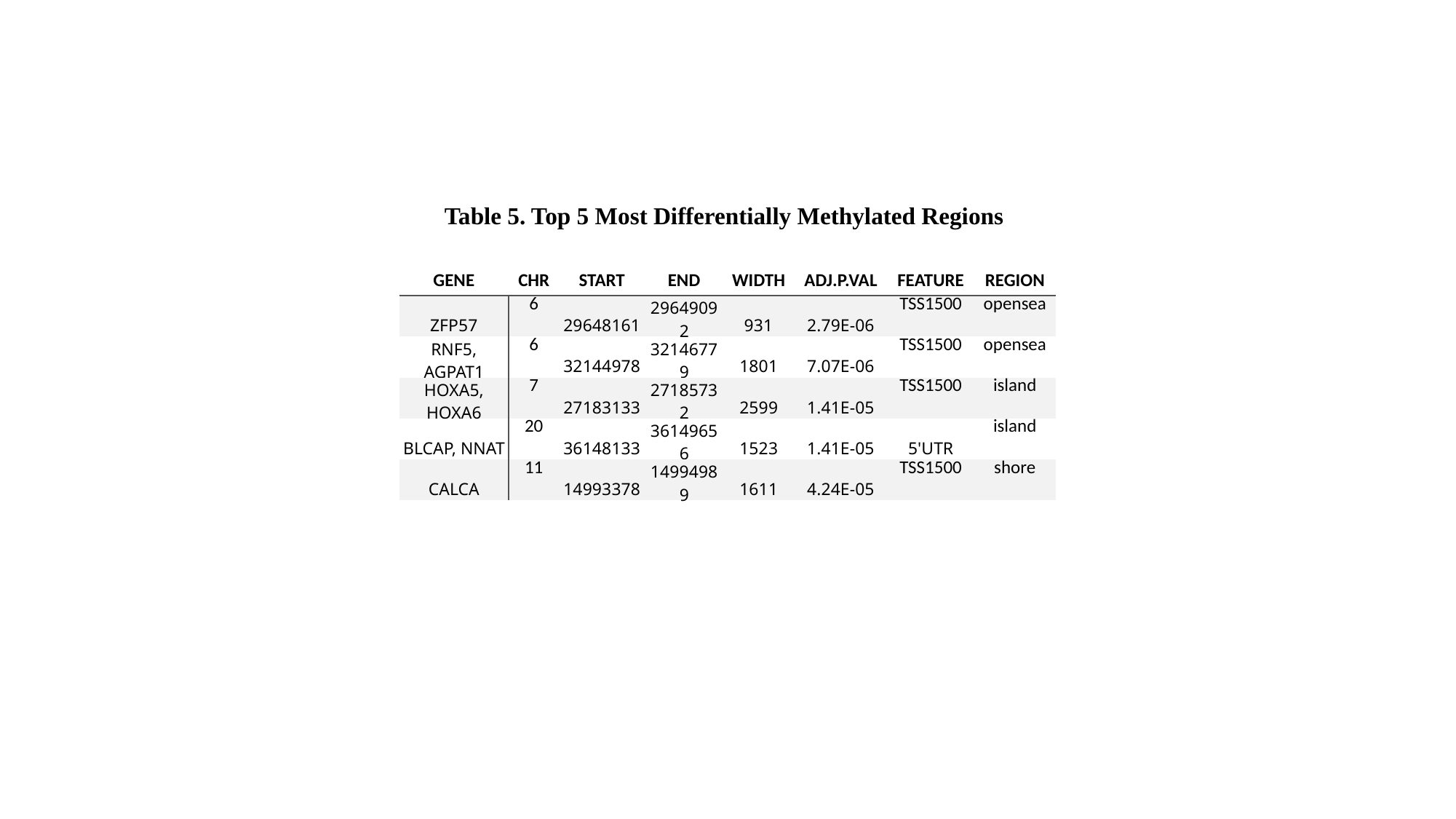

Table 5. Top 5 Most Differentially Methylated Regions
| gene | cHR | START | END | WIDTH | adj.P.Val | feature | region |
| --- | --- | --- | --- | --- | --- | --- | --- |
| ZFP57 | 6 | 29648161 | 29649092 | 931 | 2.79E-06 | TSS1500 | opensea |
| RNF5, AGPAT1 | 6 | 32144978 | 32146779 | 1801 | 7.07E-06 | TSS1500 | opensea |
| HOXA5, HOXA6 | 7 | 27183133 | 27185732 | 2599 | 1.41E-05 | TSS1500 | island |
| BLCAP, NNAT | 20 | 36148133 | 36149656 | 1523 | 1.41E-05 | 5'UTR | island |
| CALCA | 11 | 14993378 | 14994989 | 1611 | 4.24E-05 | TSS1500 | shore |

## Slide 8
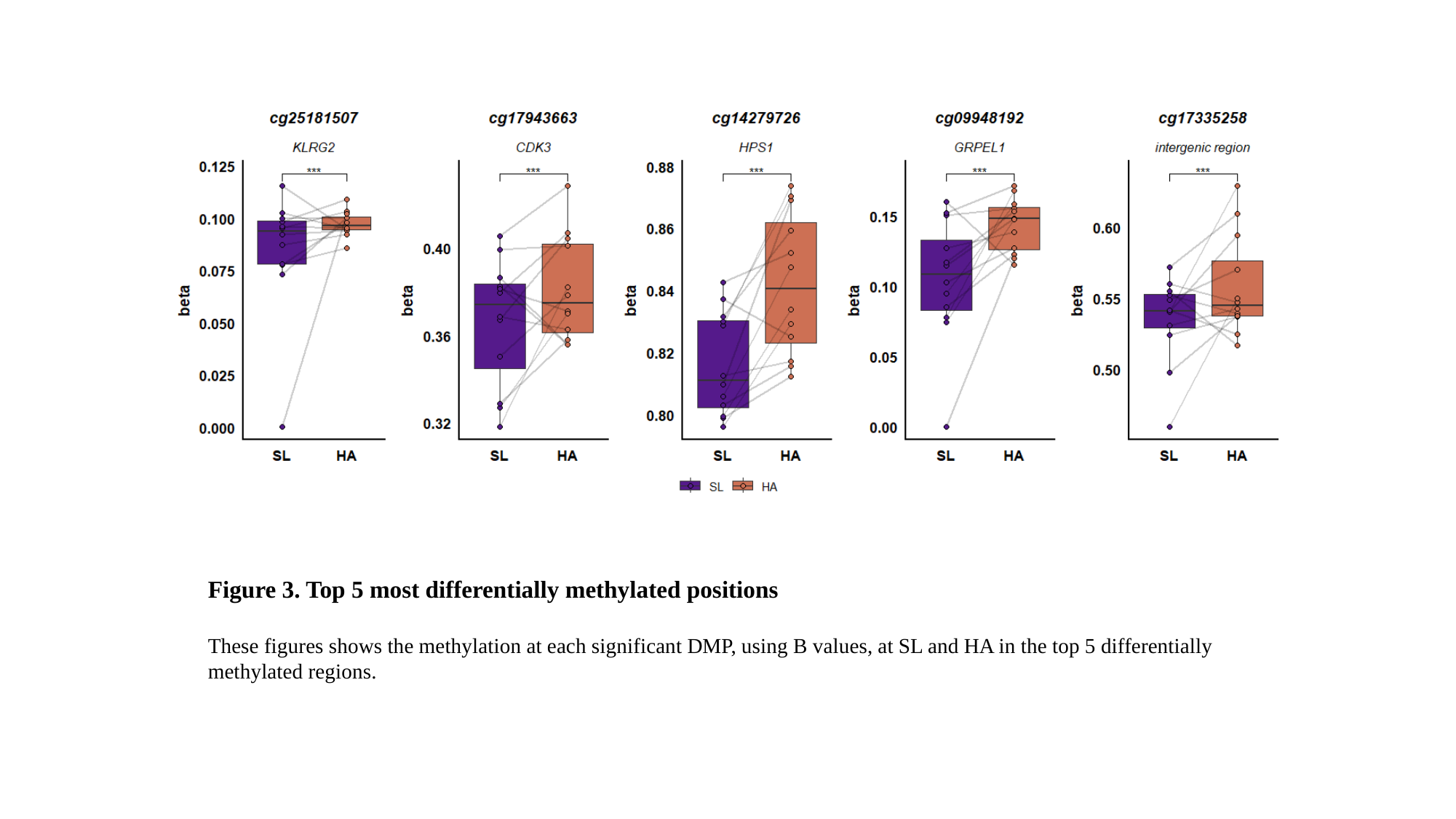

Figure 3. Top 5 most differentially methylated positions
These figures shows the methylation at each significant DMP, using B values, at SL and HA in the top 5 differentially methylated regions.

## Slide 9
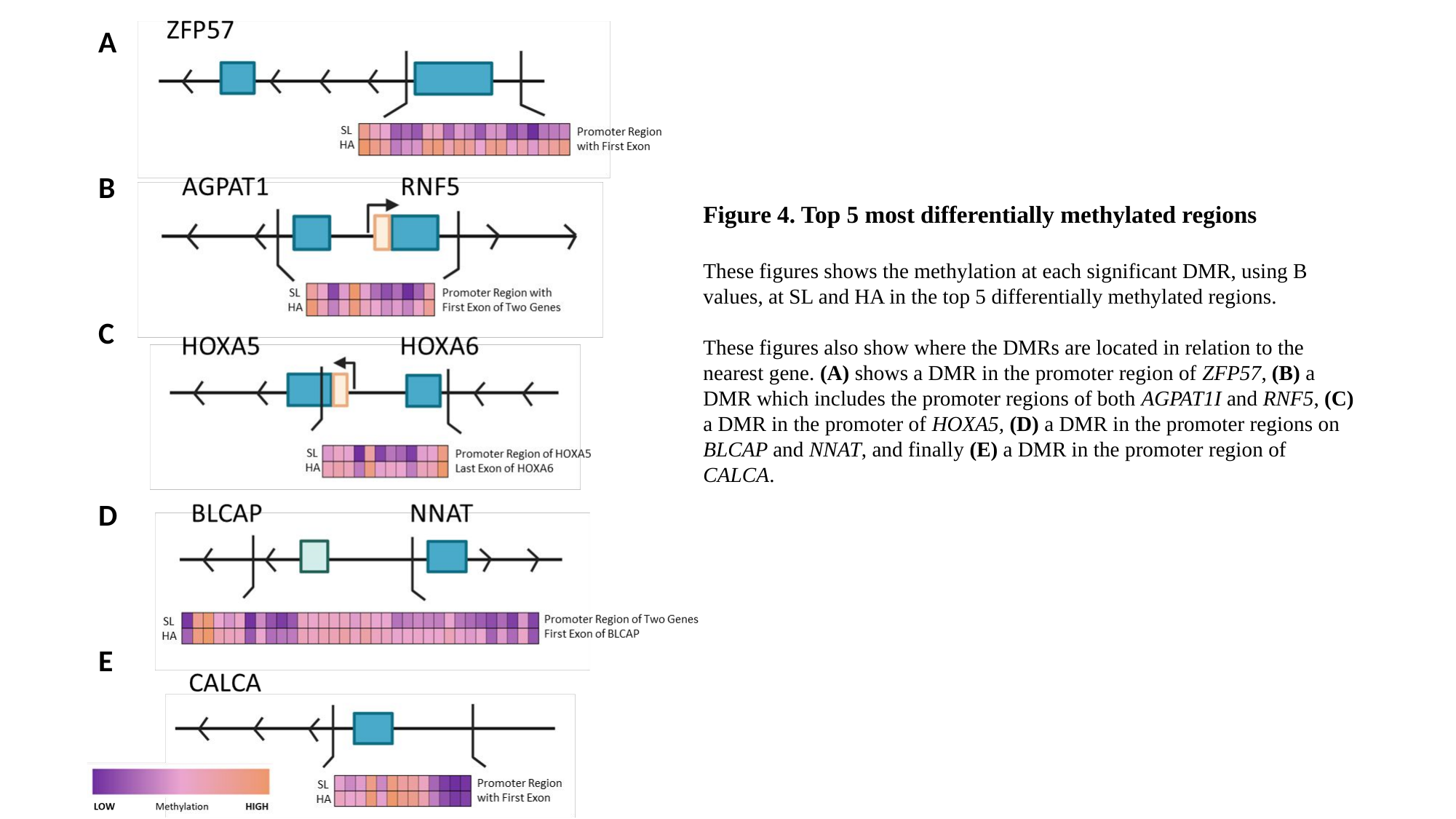

A
B
C
D
E
Figure 4. Top 5 most differentially methylated regions
These figures shows the methylation at each significant DMR, using B values, at SL and HA in the top 5 differentially methylated regions.
These figures also show where the DMRs are located in relation to the nearest gene. (A) shows a DMR in the promoter region of ZFP57, (B) a DMR which includes the promoter regions of both AGPAT1I and RNF5, (C) a DMR in the promoter of HOXA5, (D) a DMR in the promoter regions on BLCAP and NNAT, and finally (E) a DMR in the promoter region of CALCA.

## Slide 10
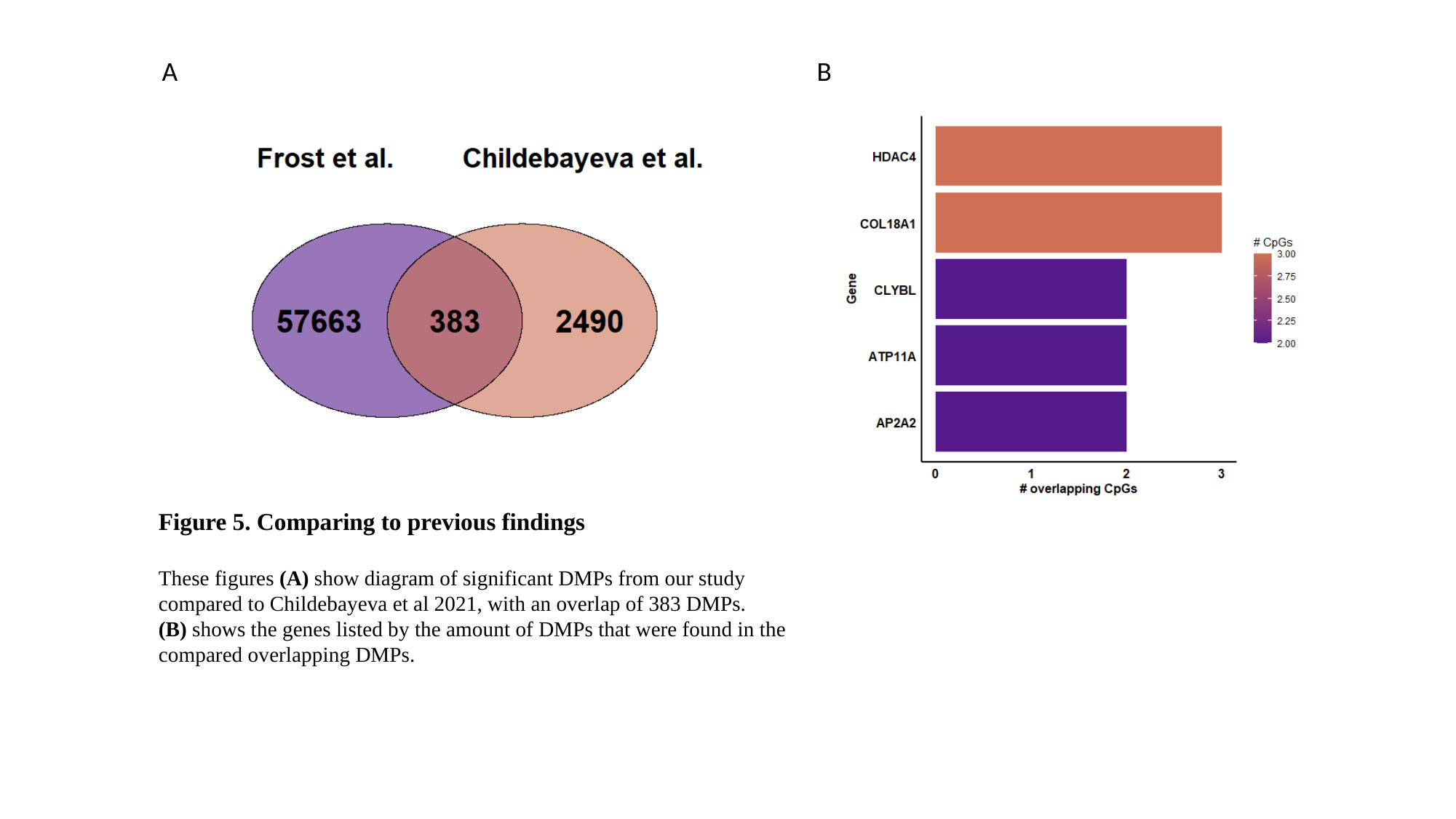

A						B
Figure 5. Comparing to previous findings
These figures (A) show diagram of significant DMPs from our study compared to Childebayeva et al 2021, with an overlap of 383 DMPs.
(B) shows the genes listed by the amount of DMPs that were found in the compared overlapping DMPs.
