# Supplementary material for: Genome-wide DNA methylation changes after 24 hours at high altitude
Source: Environ Epigenet. 2026 Feb 9;12(1):dvag004. doi: 10.1093/eep/dvag004 (PMC12951794; doi:10.1093/eep/dvag004)
Supplement: dvag004_Supplemental_Files [file dvag004_supplemental_files.zip › Supplemental Tables_Resubmit.pdf]

Supplemental 1 - Significant DMPs

cgID	logFC	AveExpr	t	P.Value	adj.P.Val	B	SL_AVG	HA_AVG	deltaBeta	CHR	MAPINFO	Strand	Type	gene	feature	cgi	feat.cgi	UCSC_Islar	SNP_ID	SNP_DISTANCE
cg2518150	-0.10657	0.096269	-14.00596	5.59E-13	4.14E-07	19.6906	0.042984	0.149553	0.10657	7	1.39E+08	R	I	KLRG2	TSS1500	island	TSS1500-is	chr7:13916	rs5668983	48;46;45
cg1794366	-0.061772	0.373834	-13.55999	1.11E-12	4.14E-07	18.99261	0.342948	0.40472	0.061772	17	74001963	F	II	CDK3	3'UTR	shore	3'UTR-shor	chr17:7400	rs5283369	0;36
cg1427972	-0.05802	0.828923	-12.92987	3.03E-12	6.13E-07	17.97427	0.799913	0.857932	0.05802	10	1E+08	F	II	HPS1	TSS1500	opensea	TSS1500-opensea			
cg0994819	-0.164015	0.134276	-12.87741	3.29E-12	6.13E-07	17.88775	0.052268	0.216283	0.164015	4	7069943	R	I	GRPEL1	TSS200	island	TSS200-isla	chr4:70690	rs3822269;	44;35;31;27
cg1733525	-0.078883	0.547129	-12.23012	9.61E-12	1.43E-06	16.79692	0.507687	0.58657	0.078883	2	1.13E+08	F	II		IGR	opensea	IGR-opensea		rs4849116;	11;13;43
cg0064717	-0.055835	0.565167	-12.06468	1.27E-11	1.51E-06	16.51109	0.537249	0.593084	0.055835	11	72396048	R	II		IGR	opensea	IGR-opensea			
cg2393173	-0.076182	0.703055	-12.00061	1.42E-11	1.51E-06	16.39961	0.664964	0.741146	0.076182	15	63673013	R	II	CA12	Body	shore	Body-shore	chr15:6367	rs1179286	17
cg0970821	-0.07345	0.459057	-11.80841	1.98E-11	1.84E-06	16.06251	0.422332	0.495782	0.07345	11	35607098	R	II		IGR	opensea	IGR-opensea		rs1119808	41
cg2738652	-0.072639	0.379195	-11.72806	2.27E-11	1.88E-06	15.92039	0.342876	0.415515	0.072639	3	47517807	R	II	SCAP	TSS1500	shore	TSS1500-sh	chr3:47516	rs1171666	36;35;27;2
cg0567490	0.072697	0.057802	11.51902	3.28E-11	2.44E-06	15.54727	0.09415	0.021453	-0.072697	9	1.4E+08	R	II		IGR	island	IGR-island	chr9:13959	rs3764154	48
cg0173163	-0.067968	0.717372	-11.3369	4.53E-11	2.80E-06	15.21821	0.683388	0.751356	0.067968	5	9947503	R	II		IGR	opensea	IGR-opensea		rs1688387	45;13
cg2569058	-0.040942	0.775231	-11.31642	4.69E-11	2.80E-06	15.18095	0.75476	0.795702	0.040942	15	60285478	R	I		IGR	shore	IGR-shore	chr15:6028	rs1244049	35;34;22
cg0627802	-0.085444	0.425529	-11.18974	5.89E-11	2.80E-06	14.94953	0.382807	0.468251	0.085444	22	31523757	R	II	INPP5J	Body	opensea	Body-opensea		rs5401052	39;34;1
cg2161863	-0.057153	0.862739	-11.16972	6.11E-11	2.80E-06	14.91279	0.834163	0.891316	0.057153	4	1984950	R	II	WHSC2	3'UTR	shelf	3'UTR-shelf	chr4:19874	rs3773873	15;9
cg1931553	-0.066121	0.4609	-11.16896	6.11E-11	2.80E-06	14.9114	0.42784	0.493961	0.066121	5	1.12E+08	R	II		IGR	opensea	IGR-opensea		rs1407384	45
cg1095690	-0.050859	0.827615	-11.16508	6.16E-11	2.80E-06	14.90428	0.802186	0.853045	0.050859	11	66335374	R	II	CTSF	Body	shore	Body-shore	chr11:6633	rs5533979	30;22;3
cg1381827	-0.06784	0.562699	-11.1436	6.40E-11	2.80E-06	14.86479	0.528779	0.596619	0.06784	22	32437865	R	II	SLC5A1	TSS1500	shore	TSS1500-sh	chr22:3243	rs5583595	11;8;6;2
cg1425293	-0.06651	0.855954	-10.99951	8.31E-11	3.14E-06	14.59853	0.822699	0.889209	0.06651	11	75153827	F	II	GDPD5	Body	opensea	Body-opensea		rs5454740	8
cg1349579	-0.059975	0.729684	-10.99011	8.45E-11	3.14E-06	14.58108	0.699697	0.759672	0.059975	17	46717953	F	II		IGR	shore	IGR-shore	chr17:4671	rs5755704	1;21
cg0824384	-0.046123	0.164236	-10.98947	8.46E-11	3.14E-06	14.57989	0.141175	0.187298	0.046123	1	31539007	R	II	PUM1	TSS1500	shore	TSS1500-sh	chr1:31538	rs5303838	43;3
cg0947626	-0.042663	0.884613	-10.92543	9.51E-11	3.14E-06	14.46071	0.863281	0.905945	0.042663	22	28026705	R	II		IGR	opensea	IGR-opensea		rs5380516	35;32;31;27;20;16
cg1249808	-0.059657	0.159038	-10.9248	9.52E-11	3.14E-06	14.45953	0.12921	0.188867	0.059657	6	43253387	F	II	TTBK1	3'UTR	island	3'UTR-islan	chr6:43252	rs5326437	45
cg0559654	0.134752	0.136251	10.91369	9.72E-11	3.14E-06	14.43879	0.203627	0.068875	-0.134752	14	94640572	F	I	PPP4R4	TSS200	island	TSS200-isla	chr14:9464	rs5531431	11;12;35;47
cg0370844	-0.064991	0.474948	-10.86522	1.06E-10	3.29E-06	14.3482	0.442453	0.507444	0.064991	3	5347184	F	II		IGR	opensea	IGR-opensea		rs1423294	1
cg2182167	-0.078344	0.731442	-10.8152	1.16E-10	3.36E-06	14.2544	0.69227	0.770614	0.078344	1	16063471	F	II	SLC25A34	Body	opensea	Body-opensea		rs5577808	10
cg0535945	-0.054367	0.295934	-10.74589	1.32E-10	3.36E-06	14.12395	0.268751	0.323118	0.054367	9	73085509	R	II		IGR	opensea	IGR-opensea		rs5607015	5
cg2014110	-0.043856	0.040403	-10.74503	1.33E-10	3.36E-06	14.12233	0.018475	0.062331	0.043856	3	48343184	R	I	NME6	TSS1500	island	TSS1500-is	chr3:48342	rs5457402	22
cg2542968	-0.060871	0.407531	-10.73948	1.34E-10	3.36E-06	14.11186	0.377095	0.437966	0.060871	2	1.77E+08	R	II		IGR	opensea	IGR-opensea		rs5606684	6
cg0892772	-0.078581	0.560948	-10.73325	1.35E-10	3.36E-06	14.1001	0.521658	0.600239	0.078581	1	54059983	F	II	GLIS1	Body	opensea	Body-opensea		rs1497066	12;13;17;33
cg0883355	-0.074686	0.432344	-10.7252	1.37E-10	3.36E-06	14.08491	0.395	0.469687	0.074686	22	22734960	F	II		IGR	opensea	IGR-opensea		rs5509084	11;39
cg0874559	-0.066758	0.555412	-10.68725	1.47E-10	3.36E-06	14.01315	0.522033	0.588791	0.066758	1	1.7E+08	F	II	F5	TSS1500	opensea	TSS1500-opensea		rs9332483;	33;38;40
cg1593921	-0.063183	0.726541	-10.68454	1.48E-10	3.36E-06	14.008	0.694949	0.758133	0.063183	17	17776237	R	II	TOM1L2	Body	opensea	Body-opensea			
cg0847348	-0.076472	0.274257	-10.68132	1.49E-10	3.36E-06	14.00191	0.23602	0.312493	0.076472	19	51504516	R	II	KLK8	5'UTR	shore	5'UTR-shor	chr19:5150	rs1895187	27
cg2216385	-0.058248	0.282026	-10.66002	1.55E-10	3.39E-06	13.96153	0.252902	0.31115	0.058248	3	1.27E+08	F	II		IGR	opensea	IGR-opensea		rs1168383	0;1
cg0328874	-0.063313	0.704629	-10.61723	1.68E-10	3.57E-06	13.88027	0.672972	0.736285	0.063313	21	45231149	F	II	AATBC	Body	opensea	Body-opensea		rs5432238	12;31
cg2217695	-0.052661	0.126769	-10.59318	1.76E-10	3.63E-06	13.8345	0.100438	0.153099	0.052661	7	22589311	R	II		IGR	island	IGR-island	chr7:22589	rs5436008	15;3
cg0023847	-0.062877	0.619495	-10.51273	2.04E-10	3.99E-06	13.68088	0.588056	0.650933	0.062877	1	36616845	R	II	TRAPPC3	5'UTR	shore	5'UTR-shor	chr1:36614	rs7422494	47;14
cg1398893	-0.024457	0.959407	-10.50372	2.08E-10	3.99E-06	13.66363	0.947178	0.971635	0.024457	16	3037149	R	II		IGR	opensea	IGR-opensea			
cg2756152	-0.065678	0.591827	-10.49991	2.09E-10	3.99E-06	13.65634	0.558989	0.624666	0.065678	18	57314113	R	II	CCBE1	Body	opensea	Body-opensea		rs1379721	46;26;25
cg0981526	-0.084721	0.63682	-10.47919	2.18E-10	4.05E-06	13.6166	0.59446	0.67918	0.084721	11	17664258	R	II	OTOG	Body	opensea	Body-opensea			
cg0853292	-0.042039	0.896926	-10.45935	2.26E-10	4.09E-06	13.57853	0.875906	0.917946	0.042039	10	81939751	R	II	ANXA11	5'UTR	opensea	5'UTR-opensea		rs5372294	27;21;11;2;1
cg1158052	-0.065177	0.642234	-10.44741	2.31E-10	4.09E-06	13.55557	0.609645	0.674823	0.065177	11	1.18E+08	R	II	SCN4B	Body	shore	Body-shore	chr11:1180	rs5669081	48;5
cg2384361	-0.073157	0.373499	-10.40503	2.50E-10	4.29E-06	13.47399	0.336921	0.410078	0.073157	18	45711790	R	II		IGR	opensea	IGR-opensea		rs5591391	20
cg1168346	-0.05801	0.772696	-10.39666	2.54E-10	4.29E-06	13.45787	0.743691	0.8017	0.05801	16	85169085	F	II		IGR	shore	IGR-shore	chr16:85171019-85171323		
cg0987013	-0.066872	0.436714	-10.37904	2.63E-10	4.29E-06	13.42386	0.403278	0.47015	0.066872	22	46320026	F	II	WNT7B	Body	shore	Body-shore	chr22:4631	rs5744366	11
cg1708012	-0.039881	0.876747	-10.3741	2.65E-10	4.29E-06	13.41433	0.856806	0.896688	0.039881	5	1.72E+08	R	II		IGR	opensea	IGR-opensea			
cg1209094	-0.052562	0.552869	-10.36029	2.72E-10	4.31E-06	13.38764	0.526589	0.57915	0.052562	14	77279508	R	II	ANGEL1	TSS1500	shore	TSS1500-sh	chr14:7727	rs1893435	28;1
cg2680791	-0.074884	0.666318	-10.34154	2.82E-10	4.37E-06	13.35139	0.628876	0.70376	0.074884	10	74042258	R	II		IGR	opensea	IGR-opensea		rs5778015	21
cg1449544	-0.053722	0.797882	-10.32042	2.94E-10	4.44E-06	13.31047	0.771022	0.824743	0.053722	17	76236127	F	II	LOC28399	3'UTR	opensea	3'UTR-opensea			
cg1899404	-0.053051	0.81192	-10.31169	2.99E-10	4.44E-06	13.29356	0.785394	0.838445	0.053051	11	69813825	R	II		IGR	shore	IGR-shore	chr11:6981	rs5498547	50;24;18;2
cg2396754	-0.057994	0.055953	-10.30197	3.04E-10	4.44E-06	13.2747	0.026956	0.08495	0.057994	8	21906746	R	I		IGR	shore	IGR-shore	chr8:21905461-21905757		
cg2075313	-0.059437	0.22127	-10.22792	3.50E-10	5.01E-06	13.1307	0.191551	0.250989	0.059437	3	1.22E+08	F	II	CD86	TSS200	opensea	TSS200-opensea		rs9282643;	1;39;48
cg0165218	-0.05987	0.860044	-10.17034	3.91E-10	5.33E-06	13.01825	0.830109	0.889979	0.05987	3	53905370	R	II	ACTR8	Body	opensea	Body-opensea		rs5690410	13
cg1802206	-0.064549	0.779774	-10.1549	4.03E-10	5.33E-06	12.98802	0.7475	0.812049	0.064549	6	6657777	R	II		IGR	opensea	IGR-opensea		rs5299337	11
cg0840148	-0.070942	0.316926	-10.15199	4.05E-10	5.33E-06	12.98232	0.281455	0.352397	0.070942	14	91162832	R	II	TTC7B	Body	opensea	Body-opensea		rs5490594	25
cg1662118	-0.065251	0.626725	-10.1499	4.07E-10	5.33E-06	12.97823	0.594099	0.659351	0.065251	19	50006799	F	II		IGR	opensea	IGR-opensea		rs1823573	1;10;38
cg1654438	-0.067004	0.57662	-10.14783	4.08E-10	5.33E-06	12.97417	0.543118	0.610121	0.067004	22	22519402	F	II		IGR	opensea	IGR-opensea		rs1434748	30
cg0091901	-0.08225	0.236006	-10.13891	4.15E-10	5.33E-06	12.9567	0.19488	0.277131	0.08225	8	12613847	F	II	LONRF1	TSS1500	shore	TSS1500-sh	chr8:12611	rs4831364	37
cg2327052	-0.064692	0.651747	-10.12667	4.25E-10	5.36E-06	12.93268	0.619401	0.684093	0.064692	18	4450489	R	II		IGR	shelf	IGR-shelf	chr18:4453	rs5484616	1
cg1683052	-0.059477	0.64175	-10.0996	4.48E-10	5.49E-06	12.87954	0.612011	0.671488	0.059477	22	39957232	F	II		IGR	shelf	IGR-shelf	chr22:3995	rs1170478	1;34
cg1350524	-0.08734	0.534271	-10.09755	4.50E-10	5.49E-06	12.87552	0.490601	0.57794	0.08734	10	1.04E+08	F	II	SUFU	Body	opensea	Body-opensea		rs5600719	13;24;25
cg2066326	-0.10151	0.721873	-10.08716	4.59E-10	5.51E-06	12.85507	0.671119	0.772628	0.10151	2	98910878	R	II	VWA3B	Body	opensea	Body-opensea		rs1017706	27;23;22;15
cg1801014	-0.042017	0.876532	-10.06738	4.77E-10	5.63E-06	12.81616	0.855523	0.89754	0.042017	9	1.25E+08	F	II		IGR	opensea	IGR-opensea			
cg1329170	-0.063728	0.055834	-10.03675	5.06E-10	5.88E-06	12.75577	0.023971	0.087698	0.063728	10	15761498	R	I	ITGA8	Body	island	Body-island	chr10:1576	rs5551943	47;18;1
cg2583896	-0.079591	0.480498	-10.01511	5.27E-10	5.91E-06	12.71305	0.440703	0.520294	0.079591	1	2.08E+08	F	II	PLXNA2	Body	opensea	Body-opensea		rs5364155	43
cg1584471	-0.062679	0.524274	-10.01181	5.31E-10	5.91E-06	12.70652	0.492935	0.555613	0.062679	11	59435407	R	II	PATL1	Body	shore	Body-shore	chr11:5943	rs5592144	17;15;1
cg2667136	-0.070999	0.448418	-10.00885	5.34E-10	5.91E-06	12.70067	0.412919	0.483918	0.070999	2	1.1E+08	F	II	EDAR	1stExon	opensea	1stExon-opensea		rs1923238	26;35
cg1841425	0.03704	0.933919	9.992439	5.51E-10	5.91E-06	12.66822	0.952439	0.915399	-0.03704	4	1.14E+08	F	II	ANK2	Body	opensea	Body-opensea		rs5675702	14;17;18;36;50
cg0458885	-0.081249	0.615773	-9.989668	5.54E-10	5.91E-06	12.66274	0.575149	0.656398	0.081249	9	1.32E+08	R	II		IGR	opensea	IGR-opensea		rs7363099	35
cg1793188	-0.054312	0.303688	-9.987967	5.56E-10	5.91E-06	12.65937	0.276532	0.330844	0.054312	2	2.08E+08	F	II	LOC10192	Body	opensea	Body-opensea			
cg1165238	-0.063913	0.455633	-9.960009	5.87E-10	6.15E-06	12.60399	0.423676	0.487589	0.063913	19	34130155	F	II	CHST8	5'UTR	opensea	5'UTR-opensea		rs1119650	0
cg2619963	-0.072968	0.554375	-9.932759	6.19E-10	6.24E-06	12.54991	0.51789	0.590859	0.072968	2	2.4E+08	F	II		IGR	opensea	IGR-opensea		rs5502809	0;4
cg0245082	-0.124589	0.663111	-9.921325	6.33E-10	6.24E-06	12.52719	0.600817	0.725405	0.124589	7	1.51E+08	R	II	IQCA1L	Body	opensea	Body-opensea		rs1878664	15
cg1400257	-0.057247	0.805071	-9.920643	6.34E-10	6.24E-06	12.52584	0.776448	0.833695	0.057247	9	1.02E+08	F	II	GALNT12	TSS1500	shore	TSS1500-sh	chr9:10156	rs1907686	1;33
cg0876243	-0.085639	0.506022	-9.920171	6.34E-10	6.24E-06	12.5249	0.463203	0.548842	0.085639	10	21358991	F	II	NEBL	Body	opensea	Body-opensea		rs7616853	27;35
cg0444238	-0.068148	0.304035	-9.917395	6.38E-10	6.24E-06	12.51938	0.269961	0.338109	0.068148	7	43151168	F	II	HECW1	TSS1500	shore	TSS1500-sh	chr7:43152	rs1856479	5;37
cg0951245	-0.054856	0.654429	-9.910645	6.46E-10	6.24E-06	12.50596	0.627	0.681857	0.054856	11	62120239	R	II	ASRGL1	Body	opensea	Body-opensea			
cg2323145	-0.059478	0.528883	-9.880083	6.86E-10	6.54E-06	12.44511	0.499144	0.558622	0.059478	3	1.36E+08	F	II		IGR	opensea	IGR-opensea		rs3729203	7;15
cg1494480	-0.110095	0.72807	-9.838208	7.44E-10	6.91E-06	12.36155	0.673022	0.783117	0.110095	19	7736718	F	II		IGR	shore	IGR-shore	chr19:7735	rs1485291	1;15;23;32;39;40
cg2099023	-0.041103	0.204425	-9.828935	7.58E-10	6.91E-06	12.34302	0.183874	0.224977	0.041103	12	322708	R	II	SLC6A12	5'UTR	opensea	5'UTR-opensea		rs5439334	34;20
cg2228701	-0.046436	0.200956	-9.821977	7.68E-10	6.91E-06	12.3291	0.177738	0.224174	0.046436	6	1.5E+08	R	II		IGR	island	IGR-island	chr6:15035	rs1419947	50;35;13
cg0258443	-0.055562	0.254366	-9.81965	7.72E-10	6.91E-06	12.32445	0.226585	0.282147	0.055562	5	1.39E+08	R	II	SPATA24	TSS1500	shore	TSS1500-sh	chr5:13873	rs1136184	45
cg0664294	-0.083103	0.798453	-9.814142	7.80E-10	6.91E-06	12.31342	0.756901	0.840005	0.083103	6	1.64E+08	F	II	PACRG	Body	shore	Body-shore	chr6:16357	rs1168811	27;38
cg2093861	-0.07007	0.570875	-9.808927	7.88E-10	6.91E-06	12.30299	0.53584	0.60591	0.07007	5	1.72E+08	F	II		IGR	opensea	IGR-opensea		rs1919579	20
cg1627625	-0.088821	0.576786	-9.807859	7.90E-10	6.91E-06	12.30085	0.532375	0.621196	0.088821	4	8205451	R	II	SH3TC1	5'UTR	opensea	5'UTR-opensea		rs5701019	38;23;2
cg1096547	-0.067625	0.239336	-9.793898	8.12E-10	7.02E-06	12.27289	0.205523	0.273148	0.067625	15	31617633	R	II	KLF13	TSS1500	shore	TSS1500-sh	chr15:3161	rs5564521	2
cg0735421	-0.07024	0.60151	-9.783928	8.28E-10	7.05E-06	12.2529	0.56639	0.63663	0.07024	3	55475778	F	II		IGR	opensea	IGR-opensea		rs5367251	27;34
cg0057135	-0.051524	0.681868	-9.778266	8.37E-10	7.05E-06	12.24155	0.656107	0.70763	0.051524	7	1.51E+08	R	II	KCNH2	Body	opensea	Body-opensea		rs5487246	25;17;10
cg0995942	-0.056582	0.301152	-9.774704	8.43E-10	7.05E-06	12.2344	0.272861	0.329443	0.056582	11	61322107	R	II	SYT7	Body	shore	Body-shore	chr11:6132	rs1813612	45;20
cg0458539	-0.430759	0.530967	-9.748647	8.87E-10	7.34E-06	12.18208	0.315588	0.746347	0.430759	17	18918311	R	I	SLC5A10	Body	island	Body-island	chr17:1891	rs5274958	44;43
cg0217281	-0.061068	0.578999	-9.7315	9.18E-10	7.50E-06	12.14761	0.548464	0.609533	0.061068	10	79261503	R	II	KCNMA1	Body	opensea	Body-opensea		rs5446710	43;1
cg2586123	-0.104309	0.662638	-9.712613	9.53E-10	7.70E-06	12.10959	0.610484	0.714793	0.104309	8	21778963	F	II	XPO7	Body	shore	Body-shore	chr8:21776842-21777942		
cg1509112	-0.074369	0.687365	-9.702649	9.71E-10	7.77E-06	12.08952	0.65018	0.724549	0.074369	12	1.23E+08	F	II	LRRC43	Body	shelf	Body-shelf	chr12:122687800-122689106		
cg0348820	-0.076678	0.72475	-9.690786	9.95E-10	7.87E-06	12.0656	0.686411	0.763089	0.076678	16	84209273	R	II	DNAAF1	Body	opensea	Body-opensea		rs1862350	46;40;15;2;1
cg0049491	-0.164287	0.178884	-9.679151	1.02E-09	7.93E-06	12.04212	0.09674	0.261027	0.164287	8	50821935	F	II	SNTG1	TSS1500	shore	TSS1500-sh	chr8:50822	rs1882506	10;15;19
cg0481402	-0.077495	0.557995	-9.676626	1.02E-09	7.93E-06	12.03702	0.519247	0.596742	0.077495	17	75693889	F	II		IGR	opensea	IGR-opensea		rs5749060	24;25;29
cg0488347	-0.063525	0.216519	-9.671013	1.03E-09	7.93E-06	12.02569	0.184756	0.248282	0.063525	11	87080622	R	II		IGR	opensea	IGR-opensea		rs5328991	51;25;2
cg1855335	-0.049053	0.390501	-9.666149	1.04E-09	7.93E-06	12.01587	0.365975	0.415028	0.049053	5	5307308	R	II	ADAMTS16	Body	shelf	Body-shelf	chr5:53032	rs3774652	45;43;40;29;1
cg2367310	-0.067106	0.332444	-9.639844	1.10E-09	8.25E-06	11.96269	0.298892	0.365997	0.067106	19	11908712	F	II	ZNF491	TSS1500	shore	TSS1500-sh	chr19:1190	rs5590224	38

cg0112705	-0.04678	0.613908	-9.635793	1.11E-09	8.25E-06	11.95449	0.590518	0.637298	0.04678	16	3022847	F	II	PKMYT1	3'UTR	shelf	3'UTR-shelf	chr16:3016	rs3720842	0;10;19;31
cg1374261	-0.041287	0.593663	-9.621273	1.14E-09	8.36E-06	11.92509	0.573019	0.614306	0.041287	2	2.32E+08	R	II		IGR	opensea	IGR-opensea		rs5341428	45;41;29;8
cg1302362	0.138759	0.153246	9.619017	1.15E-09	8.36E-06	11.92052	0.222626	0.083867	-0.138759	14	57276257	R	I	OTX2	5'UTR	island	5'UTR-islan	chr14:5727	rs5761424	6
cg2058804	-0.060326	0.250946	-9.594571	1.20E-09	8.58E-06	11.87095	0.220783	0.281109	0.060326	10	56561096	R	II	PCDH15	TSS200	opensea	TSS200-opensea			
cg0470080	-0.058581	0.658405	-9.592754	1.21E-09	8.58E-06	11.86726	0.629115	0.687696	0.058581	17	80339233	F	II		IGR	opensea	IGR-opensea		rs1165302	16;36
cg0336287	-0.067428	0.622313	-9.591591	1.21E-09	8.58E-06	11.8649	0.588599	0.656027	0.067428	22	50352912	F	II	PIM3	TSS1500	shore	TSS1500-sh	chr22:50353596-50357215		
cg2535041	-0.064671	0.39899	-9.581681	1.23E-09	8.67E-06	11.84478	0.366654	0.431325	0.064671	6	1.44E+08	R	II	HYMAI	Body	island	Body-island	chr6:144328916-144329847		
cg1969232	-0.062649	0.443192	-9.563009	1.28E-09	8.84E-06	11.80684	0.411867	0.474516	0.062649	1	3621048	F	II	TP73	Body	shelf	Body-shelf	chr1:36233	rs1489886	1;49
cg1927597	-0.065714	0.40102	-9.562441	1.28E-09	8.84E-06	11.80568	0.368163	0.433877	0.065714	2	60983087	F	II	PAPOLG	TSS1500	shore	TSS1500-sh	chr2:60983	rs5599668	27;47
cg2679667	-0.069631	0.19901	-9.538936	1.34E-09	9.18E-06	11.75785	0.164195	0.233826	0.069631	2	2.32E+08	F	II		IGR	shore	IGR-shore	chr2:232469147-232469379		
cg2665507	-0.074929	0.567544	-9.53441	1.36E-09	9.18E-06	11.74864	0.530079	0.605008	0.074929	17	21227099	R	II		IGR	opensea	IGR-opensea		rs2014232	24;23
cg0880409	-0.056193	0.596969	-9.529263	1.37E-09	9.19E-06	11.73815	0.568872	0.625065	0.056193	2	11239565	R	II		IGR	opensea	IGR-opensea		rs5641471	14;2;1
cg2589005	-0.06695	0.814265	-9.519735	1.40E-09	9.28E-06	11.71873	0.780789	0.84774	0.06695	8	41255897	R	II		IGR	opensea	IGR-opensea		rs1870767	51;1
cg1580015	-0.062399	0.41612	-9.514205	1.41E-09	9.30E-06	11.70745	0.38492	0.447319	0.062399	12	53346243	R	II	KRT18	Body	shelf	Body-shelf	chr12:5334	rs1485046	10;1
cg0220827	-0.029673	0.91089	-9.5078	1.43E-09	9.34E-06	11.69439	0.896053	0.925726	0.029673	13	46287777	R	II	SPERT	Body	island	Body-island	chr13:4628	rs1391900	1
cg2256674	-0.069733	0.589992	-9.494497	1.47E-09	9.36E-06	11.66723	0.555125	0.624859	0.069733	12	52915646	R	II	KRT5	TSS1500	opensea	TSS1500-opensea		rs7774830	50;39;34;31;20;14;2;1
cg0651114	-0.06267	0.278826	-9.488782	1.49E-09	9.36E-06	11.65556	0.241491	0.304161	0.06267	19	30469222	F	II	URI1	Body	opensea	Body-opensea		rs5299939	22
cg0426951	-0.090352	0.740388	-9.487424	1.49E-09	9.36E-06	11.65278	0.695212	0.785564	0.090352	6	1.68E+08	F	II	CCR6	5'UTR	opensea	5'UTR-opensea		rs3766932	0;1;20;35;51
cg2496429	-0.079037	0.374685	-9.485692	1.50E-09	9.36E-06	11.64925	0.335166	0.414203	0.079037	1	1.56E+08	R	II		IGR	opensea	IGR-opensea		rs5512399	47;23;17;1
cg2459669	-0.051534	0.735347	-9.485152	1.50E-09	9.36E-06	11.64814	0.70958	0.761114	0.051534	2	1.31E+08	R	II	CCDC115	Body	shore	Body-shore	chr2:13109	rs5604130	51
cg1597640	-0.038058	0.851028	-9.464369	1.56E-09	9.68E-06	11.60565	0.831999	0.870057	0.038058	1	42619118	R	I	GUCA2B	1stExon	opensea	1stExon-opensea		rs2004145	46;33;32;23;2;1
cg0686713	-0.052322	0.821086	-9.458787	1.58E-09	9.71E-06	11.59422	0.794925	0.847247	0.052322	20	62583181	F	II	UCKL1	TSS1500	shelf	TSS1500-sh	chr20:6258	rs5691919	13;32;41
cg0826762	-0.06447	0.659379	-9.432211	1.66E-09	9.99E-06	11.53979	0.627144	0.691614	0.06447	14	1.05E+08	R	II	ASPG	Body	shore	Body-shore	chr14:1045	rs5308545	31;24;19;18;12
cg1196470	-0.062544	0.46008	-9.428924	1.68E-09	9.99E-06	11.53305	0.428808	0.491352	0.062544	20	1974591	R	II	PDYN	TSS1500	opensea	TSS1500-opensea		rs1143206	22
cg0552202	-0.048257	0.793291	-9.426447	1.68E-09	9.99E-06	11.52797	0.769163	0.817419	0.048257	2	10882910	R	II	ATP6V1C2	Body	opensea	Body-opensea		rs5482488	50
cg0054624	-0.070074	0.761641	-9.425313	1.69E-09	9.99E-06	11.52564	0.726603	0.796678	0.070074	1	2412743	R	II	PLCH2	Body	shore	Body-shore	chr1:24111	rs5319917	5;2
cg1173510	-0.03188	0.882081	-9.424451	1.69E-09	9.99E-06	11.52387	0.866141	0.898021	0.03188	2	2.35E+08	F	II	MROH2A	1stExon	opensea	1stExon-opensea			
cg1633000	-0.054522	0.398284	-9.419907	1.71E-09	1.00E-05	11.51455	0.371023	0.425546	0.054522	11	36384553	F	II	PRR5L	5'UTR	opensea	5'UTR-opensea		rs1904686	1;8;18;19;36
cg2265498	-0.075598	0.601785	-9.398207	1.78E-09	1.03E-05	11.47	0.563986	0.639584	0.075598	8	11721826	R	II	CTSB	5'UTR	shelf	5'UTR-shelf	chr8:11725	rs3104254	5
cg1624980	-0.087728	0.37224	-9.396354	1.79E-09	1.03E-05	11.46619	0.328377	0.416104	0.087728	1	1.97E+08	R	II	ZBTB41	Body	opensea	Body-opensea		rs5598303	40;1
cg1273650	-0.08807	0.32014	-9.388549	1.82E-09	1.04E-05	11.45015	0.276106	0.364175	0.08807	2	1.21E+08	F	II		IGR	opensea	IGR-opensea		rs5703561	35;51
cg0316755	-0.044797	0.161312	-9.376936	1.86E-09	1.06E-05	11.42627	0.138914	0.183711	0.044797	16	81347858	R	II	GAN	TSS1500	shore	TSS1500-sh	chr16:8134	rs7556844	15
cg1990703	-0.104595	0.15546	-9.369816	1.89E-09	1.06E-05	11.41161	0.103162	0.207757	0.104595	16	50727262	R	II		IGR	opensea	IGR-opensea		rs1158700	25;21
cg1420837	-0.03947	0.744765	-9.357291	1.94E-09	1.06E-05	11.38582	0.72503	0.764499	0.03947	6	3176347	F	II		IGR	opensea	IGR-opensea		rs5422700	1;25;26;42;48
cg0150118	-0.047195	0.207982	-9.354942	1.95E-09	1.06E-05	11.38099	0.184385	0.23158	0.047195	14	24881456	R	II	NYNRIN	Body	opensea	Body-opensea		rs1443623	1
cg1879762	-0.055844	0.701622	-9.349546	1.97E-09	1.06E-05	11.36987	0.6737	0.729544	0.055844	8	86083994	F	II		IGR	opensea	IGR-opensea		rs5665317	0
cg1303362	-0.040629	0.042365	-9.347585	1.97E-09	1.06E-05	11.36583	0.022051	0.06268	0.040629	12	7342228	F	I	PEX5	TSS1500	island	TSS1500-is	chr12:7342074-7342417		
cg0054755	-0.04216	0.273367	-9.3473	1.98E-09	1.06E-05	11.36524	0.252287	0.294446	0.04216	3	1.72E+08	R	II		IGR	opensea	IGR-opensea		rs5328794	13;9
cg0265634	-0.063818	0.731824	-9.345386	1.98E-09	1.06E-05	11.36129	0.699915	0.763733	0.063818	6	13872434	R	II		IGR	shore	IGR-shore	chr6:13873	rs5763347	51;24;14;2
cg0280158	-0.16418	0.171535	-9.344595	1.99E-09	1.06E-05	11.35966	0.089444	0.253625	0.16418	22	45809602	R	I	RIBC2	5'UTR	island	5'UTR-islan	chr22:4580	rs5737387	21;15
cg2091959	-0.04049	0.913603	-9.342714	1.99E-09	1.06E-05	11.35578	0.893358	0.933849	0.04049	7	2727028	R	II	AMZ1	TSS1500	shore	TSS1500-sh	chr7:27276	rs5665438	35;22
cg1465554	-0.06122	0.74136	-9.334789	2.03E-09	1.06E-05	11.33944	0.71075	0.77197	0.06122	9	1.37E+08	R	II		IGR	opensea	IGR-opensea			
cg1312291	-0.061323	0.597686	-9.334687	2.03E-09	1.06E-05	11.33923	0.567024	0.628348	0.061323	19	18359093	F	II	PDE4C	TSS200	opensea	TSS200-opensea		rs5343128	1;5;15
cg0217463	-0.065267	0.248759	-9.329568	2.07E-09	1.07E-05	11.31965	0.216126	0.281392	0.065267	8	37824306	F	II	ADRB3	TSS200	shore	TSS200-sho	chr8:37822	rs1384723	4;16;24
cg1092196	-0.075859	0.724192	-9.323198	2.07E-09	1.07E-05	11.31552	0.686262	0.762121	0.075859	4	3647785	F	II		IGR	shelf	IGR-shelf	chr4:36437	rs1913074	1;27;42;43
cg0045336	-0.042195	0.8526	-9.319651	2.09E-09	1.07E-05	11.3082	0.831503	0.873698	0.042195	10	52121178	R	II	SGMS1	5'UTR	opensea	5'UTR-opensea		rs5751995	41
cg2574495	-0.069256	0.553873	-9.31681	2.10E-09	1.07E-05	11.30233	0.519245	0.588501	0.069256	4	7106195	R	II	FLJ36777	TSS1500	opensea	TSS1500-opensea		rs1449988	5;2;1
cg1881071	-0.112878	0.511393	-9.303608	2.16E-09	1.09E-05	11.27506	0.454954	0.567832	0.112878	6	26020251	F	II	HIST1H3A	TSS1500	shore	TSS1500-sh	chr6:26021	rs1179552	7;21
cg2526842	-0.089877	0.820413	-9.295827	2.19E-09	1.09E-05	11.25897	0.775474	0.865351	0.089877	9	1.37E+08	F	II	VAV2	Body	island	Body-island	chr9:13665	rs5307764	0;14
cg1092307	-0.063381	0.527428	-9.293905	2.20E-09	1.09E-05	11.255	0.495738	0.559119	0.063381	14	70651043	R	II	SLC8A3	Body	opensea	Body-opensea		rs5304100	17
cg0886954	-0.063479	0.633311	-9.293045	2.20E-09	1.09E-05	11.25322	0.601571	0.665051	0.063479	8	1.44E+08	F	II	ADGRB1	TSS1500	shore	TSS1500-sh	chr8:143545445-143546178		
cg0759122	-0.042949	0.814292	-9.277483	2.28E-09	1.12E-05	11.22102	0.792818	0.835767	0.042949	2	8774045	R	II		IGR	opensea	IGR-opensea		rs5626957	50;14
cg1563432	-0.072541	0.291818	-9.275023	2.29E-09	1.12E-05	11.21592	0.255547	0.328088	0.072541	2	42081166	R	II		IGR	opensea	IGR-opensea		rs751490;r	46;31;12
cg0213866	-0.06882	0.710145	-9.268298	2.32E-09	1.13E-05	11.202	0.675735	0.744554	0.06882	1	41880822	R	II		IGR	opensea	IGR-opensea		rs1430050	32
cg1795858	-0.089949	0.679466	-9.256178	2.38E-09	1.15E-05	11.17688	0.634492	0.724441	0.089949	7	92055458	R	II		IGR	opensea	IGR-opensea			
cg1547688	-0.091929	0.51087	-9.242008	2.45E-09	1.16E-05	11.14749	0.464905	0.556835	0.091929	12	14968227	F	II	C12orf69	TSS1500	opensea	TSS1500-opensea		rs1459651	1;14
cg0149422	-0.059075	0.698871	-9.23856	2.46E-09	1.16E-05	11.14034	0.669334	0.728409	0.059075	12	76754454	F	II		IGR	opensea	IGR-opensea		rs1877175	0;7
cg0083492	-0.047183	0.408294	-9.238334	2.46E-09	1.16E-05	11.13987	0.384702	0.431886	0.047183	5	1.79E+08	R	II	ADAMTS2	Body	shore	Body-shore	chr5:17854	rs7604337	20;7
cg2180811	-0.042236	0.963732	-9.23687	2.47E-09	1.16E-05	11.13683	0.942615	0.98485	0.042236	9	1.28E+08	F	II	OLFML2A	TSS1500	shore	TSS1500-sh	chr9:12753	rs5653633	1;8;15;45
cg1607603	-0.042678	0.215448	-9.230846	2.50E-09	1.17E-05	11.12432	0.194109	0.236786	0.042678	22	19744078	F	II	TBX1	TSS200	island	TSS200-isla	chr22:1974	rs5757466	51
cg1650997	-0.049429	0.317298	-9.225695	2.53E-09	1.17E-05	11.11363	0.292583	0.342012	0.049429	11	67121607	R	II	LOC10013(	Body	shore	Body-shore	chr11:6712	rs5496198	47
cg1810496	-0.085479	0.50436	-9.223265	2.54E-09	1.17E-05	11.10858	0.46162	0.547099	0.085479	11	65319518	R	II	LTBP3	Body	shore	Body-shore	chr11:6532	rs1488835	2;1
cg0853857	-0.076939	0.444586	-9.221728	2.55E-09	1.17E-05	11.10539	0.406116	0.483056	0.076939	2	44236474	F	II		IGR	opensea	IGR-opensea			
cg2125147	-0.042064	0.160193	-9.213126	2.59E-09	1.17E-05	11.08751	0.139161	0.181226	0.042064	12	1.17E+08	F	II	NCRNA001	TSS1500	opensea	TSS1500-opensea		rs5282720	1;7;22
cg0782873	-0.054776	0.178273	-9.212354	2.60E-09	1.17E-05	11.08591	0.150885	0.205661	0.054776	15	62408259	R	II		IGR	opensea	IGR-opensea		rs1107165	19
cg2608028	-0.078345	0.483449	-9.210463	2.61E-09	1.17E-05	11.08198	0.444276	0.522621	0.078345	14	39459310	F	II		IGR	opensea	IGR-opensea		rs3731162	14
cg1237682	-0.078209	0.828417	-9.205829	2.63E-09	1.17E-05	11.07234	0.789312	0.867521	0.078209	16	3020845	F	II	PAQR4	5'UTR	shore	5'UTR-shore	chr16:3016	rs5560364	0;15;23;45;51
cg0216206	-0.071222	0.444242	-9.203108	2.65E-09	1.17E-05	11.06668	0.408631	0.479853	0.071222	19	57352134	F	II	ZIM2	TSS200	island	TSS200-isla	chr19:5735	rs1120895	51
cg0778139	-0.034164	0.103219	-9.200938	2.66E-09	1.17E-05	11.06217	0.086137	0.120301	0.034164	11	69455287	R	II	CCND1	TSS1500	island	TSS1500-is	chr11:6945	rs5680198	43
cg2146841	-0.045719	0.312562	-9.199904	2.66E-09	1.17E-05	11.06002	0.289702	0.335421	0.045719	9	1.27E+08	F	II	NEK6	1stExon	shore	1stExon-sh	chr9:12702	rs5755302	41;42;48
cg1103218	-0.069978	0.716939	-9.19679	2.68E-09	1.17E-05	11.05354	0.68195	0.751928	0.069978	3	42702502	F	II	ZBTB47	Body	shelf	Body-shelf	chr3:42700	rs5712603	24;48
cg0986541	-0.038636	0.085142	-9.191893	2.71E-09	1.18E-05	11.04335	0.065823	0.10446	0.038636	18	24019173	R	II		IGR	opensea	IGR-opensea		rs5359330	44;1
cg0555087	0.043675	0.158007	9.184036	2.75E-09	1.18E-05	11.02699	0.179845	0.136169	-0.043675	20	21106597	F	I	KIZ	TSS200	shore	TSS200-sho	chr20:2110	rs5501896	1;5;20
cg0110840	-0.057191	0.771817	-9.179282	2.78E-09	1.18E-05	11.01709	0.743221	0.800412	0.057191	16	67914622	F	II	EDC4	Body	opensea	Body-opensea		rs1999927	20
cg1424484	-0.060315	0.418365	-9.178761	2.78E-09	1.18E-05	11.016	0.388208	0.448523	0.060315	4	86935922	F	II		IGR	opensea	IGR-opensea		rs5306383	7;17
cg0847907	-0.073572	0.518541	-9.17845	2.78E-09	1.18E-05	11.01536	0.481755	0.555328	0.073572	1	2.07E+08	R	II	IL20	TSS1500	opensea	TSS1500-opensea		rs5724044	25
cg2430657	-0.052531	0.733288	-9.171468	2.82E-09	1.18E-05	11.00081	0.707022	0.759553	0.052531	20	643716	F	II	SCRT2	3'UTR	shore	3'UTR-shore	chr20:6439	rs5428869	0;16;30
cg1570937	-0.085829	0.612236	-9.171093	2.83E-09	1.18E-05	11.00003	0.569321	0.655151	0.085829	8	1.02E+08	R	II	PABPC1	3'UTR	opensea	3'UTR-opensea		rs5523967	47;40
cg0807433	-0.054324	0.464922	-9.166583	2.85E-09	1.18E-05	10.99062	0.43776	0.492084	0.054324	1	38189569	F	II	EPHA10	Body	opensea	Body-opensea		rs5410410	40;49
cg1322986	-0.120956	0.329135	-9.164051	2.87E-09	1.18E-05	10.98535	0.268657	0.389613	0.120956	3	1.51E+08	F	II	MED12L	Body	opensea	Body-opensea		rs1402284	25
cg1429409	-0.055181	0.430059	-9.162609	2.88E-09	1.18E-05	10.98234	0.402469	0.45765	0.055181	11	43581118	R	II	MIR670	TSS200	opensea	TSS200-opensea		rs1497872	11;1
cg2369802	-0.06161	0.489474	-9.162426	2.88E-09	1.18E-05	10.98196	0.458669	0.520279	0.06161	8	27181813	R	II	PTK2B	TSS1500	shore	TSS1500-sh	chr8:27183	rs1849780	49;25;1
cg1664677	-0.061688	0.698146	-9.156797	2.91E-09	1.19E-05	10.97022	0.667302	0.72899	0.061688	22	48695574	R	II		IGR	opensea	IGR-opensea		rs1825339	1
cg1186658	-0.10441	0.546487	-9.154286	2.92E-09	1.19E-05	10.96498	0.494282	0.598692	0.10441	1	5804995	F	II		IGR	opensea	IGR-opensea		rs2000704	1;28;37
cg1341194	-0.057266	0.269731	-9.14894	2.96E-09	1.19E-05	10.95382	0.241098	0.298364	0.057266	2	40575296	F	II	SLC8A1	Body	opensea	Body-opensea		rs5600717	0;20
cg1752977	-0.065486	0.640874	-9.147532	2.97E-09	1.19E-05	10.95088	0.608131	0.673617	0.065486	19	56723457	F	II		IGR	opensea	IGR-opensea		rs1389437	0;24;29;35
cg0467479	-0.027628	0.042597	-9.141054	3.00E-09	1.20E-05	10.93736	0.028783	0.056411	0.027628	15	44116616	R	II	MFAP1	Body	shore	Body-shore	chr15:4411	rs1825045	26
cg2620979	-0.092767	0.266278	-9.133664	3.05E-09	1.21E-05	10.92193	0.219894	0.312662	0.092767	19	14888015	F	II	ADGRE2	5'UTR	opensea	5'UTR-opensea		rs1394557	37;38;39
cg2530419	-0.055486	0.248637	-9.12642	3.10E-09	1.22E-05	10.90679	0.220895	0.27638	0.055486	13	51168711	R	II		IGR	opensea	IGR-opensea			
cg1334760	-0.060271	0.684327	-9.124485	3.11E-09	1.22E-05	10.90275	0.654191	0.714462	0.060271	9	37957725	R	II	SHB	Body	opensea	Body-opensea		rs5701753	44;27
cg1357554	-0.061272	0.278077	-9.115522	3.17E-09	1.24E-05	10.884	0.247441	0.308713	0.061272	3	1.28E+08	F	II		IGR	shore	IGR-shore	chr3:128326937-128327507		
cg0807447	-0.045735	0.591874	-9.113134	3.18E-09	1.24E-05	10.87901	0.569006	0.614741	0.045735	17	38657949	F	II	TNS4	TSS200	opensea	TSS200-opensea		rs5701464	0;3;19;20
cg0824379	-0.067563	0.672543	-9.107254	3.22E-09	1.24E-05	10.86671	0.638762	0.706325	0.067563	8	1.27E+08	F	II		IGR	opensea	IGR-opensea		rs5627618	35;27;26
cg1627154	-0.056636	0.164746	-9.104927	3.24E-09	1.24E-05	10.86184	0.136428	0.193063	0.056636	7	1.3E+08	F	II		IGR	opensea	IGR-opensea		rs5663531	12
cg1094174	-0.057482	0.526191	-9.102641	3.25E-09	1.24E-05	10.85706	0.49745	0.554932	0.057482	15	52030519	R	II	LYSMD2	TSS200	shore	TSS200-sho	chr15:5202	rs1474768	51;16;2
cg1461643	-0.069238	0.626552	-9.09893	3.28E-09	1.24E-05	10.84929	0.591933	0.661171	0.069238	6	44116268	R	II	TMEM63B	ExonBnd	shelf	ExonBnd-sh	chr6:44119526-44119781		
cg1906385	-0.044996	0.470334	-9.090703	3.33E-09	1.24E-05	10.83206	0.447836	0.492832	0.044996	17	38350695	F	II	RAPGEFL1	3'UTR	shelf	3'UTR-shelf	chr17:3834	rs5357101	7;21;35
cg1686210	-0.041084	0.831967	-9.090139	3.34E-09	1.24E-05	10.83088	0.811425	0.852509	0.041084	2	2.42E+08	R	II		IGR	opensea	IGR-opensea		rs1395835	29;1
cg1622315	-0.047758	0.713634	-9.088827	3.34E-09	1.24E-05	10.82813	0.689755	0.737514	0.047758	7	44307836	R	II	CAMK2B	Body	opensea	Body-opensea		rs5707497	12;9;2;1
cg0494195	-0.050666	0.62358	-9.085116	3.37E-09	1.24E-05	10.82055	0.598247	0.648913	0.050666	17	4636414	F	II	MED11	Body	shore	Body-shore	chr17:4634	rs5687573	25;38;40
cg0275207	-0.05366	0.445591	-9.083144	3.38E-09	1.24E-05	10.81622	0.418761	0.472421	0.05366	3	1.55E+08	R	II		IGR	opensea	IGR-opensea			

cg2321262	-0.051135	0.678838	-9.082707	3.39E-09	1.24E-05	10.81531	0.653271	0.704405	0.051135	15	75320546	R	II	PPCDC	5'UTR	opensea	5'UTR-opensea			
cg0742437	-0.048132	0.345573	-9.079233	3.41E-09	1.24E-05	10.80802	0.321507	0.36964	0.048132	12	86658745	R	II	MGAT4C	5'UTR	opensea	5'UTR-opensea		rs1160884	44;19;13
cg0312284	-0.100522	0.299845	-9.078224	3.42E-09	1.24E-05	10.80591	0.249584	0.350106	0.100522	7	84154512	F	II		IGR	opensea	IGR-opensea		rs5621590	2;12;24;49
cg1182507	-0.03808	0.899135	-9.07809	3.42E-09	1.24E-05	10.80563	0.880096	0.918175	0.03808	7	947660	F	II	ADAP1	Body	island	Body-islanc	chr7:94736	rs5303747	1;25;26
cg1291491	-0.058794	0.523727	-9.075715	3.44E-09	1.24E-05	10.80065	0.49433	0.553124	0.058794	9	1.31E+08	R	II	PIP5KL1	Body	shore	Body-shore	chr9:13069	rs1163976	22;10
cg1880562	-0.073559	0.241624	-9.075233	3.44E-09	1.24E-05	10.79964	0.204844	0.278403	0.073559	4	10583796	F	II	CLNK	Body	opensea	Body-opensea		rs5692046	1;46;49
cg0375213	-0.059565	0.67186	-9.073329	3.45E-09	1.24E-05	10.79565	0.642078	0.701643	0.059565	17	76357232	F	II	SOCS3	TSS1500	shore	TSS1500-sł	chr17:7635	rs7399804	8
cg2073330	-0.063346	0.399005	-9.071864	3.46E-09	1.24E-05	10.79257	0.367331	0.430678	0.063346	8	1.31E+08	F	II	ASAP1	5'UTR	opensea	5'UTR-opensea		rs5740984	9;32
cg1697798	-0.048192	0.413368	-9.069595	3.48E-09	1.24E-05	10.78781	0.389272	0.437464	0.048192	19	45848082	R	II	KLC3	5'UTR	shore	5'UTR-shor	chr19:4584	rs1924480	26
cg0841513	-0.044766	0.148261	-9.066873	3.50E-09	1.24E-05	10.78211	0.125878	0.170644	0.044766	5	86176688	F	II		IGR	opensea	IGR-opensea		rs5385856	1;2
cg0953753	-0.070423	0.499727	-9.066196	3.50E-09	1.24E-05	10.78069	0.464516	0.534939	0.070423	20	592957	F	II		IGR	shore	IGR-shore	chr20:5902	rs5769345	1
cg0030579	-0.121604	0.432689	-9.060572	3.54E-09	1.24E-05	10.76888	0.371887	0.493491	0.121604	4	1.87E+08	F	II	TLR3	TSS1500	opensea	TSS1500-opensea		rs5747482	0
cg0953043	-0.122874	0.499741	-9.055442	3.58E-09	1.25E-05	10.75812	0.438304	0.561178	0.122874	2	1.43E+08	F	II	LRP1B	Body	opensea	Body-opensea		rs5460109	1;7;27
cg0060715	-0.083309	0.681415	-9.053299	3.60E-09	1.25E-05	10.75362	0.63976	0.723069	0.083309	1	37131289	F	II		IGR	opensea	IGR-opensea		rs1847038	20;30
cg0240053	-0.054344	0.338946	-9.040814	3.69E-09	1.27E-05	10.7274	0.311774	0.366118	0.054344	1	1710880	F	II	NADK	TSS1500	shore	TSS1500-sł	chr1:1709394-1710582		
cg2545380	-0.060745	0.714558	-9.039213	3.70E-09	1.27E-05	10.72403	0.684185	0.74493	0.060745	5	1.31E+08	F	II	ACSL6	Body	shore	Body-shore	chr5:13134	rs5692599	7;48;51
cg1277484	-0.040604	0.124526	-9.036683	3.72E-09	1.27E-05	10.71872	0.104224	0.144827	0.040604	14	74486312	R	II	C14orf45	Body	island	Body-islanc	chr14:74485325-74486356		
cg0759456	-0.052004	0.730192	-9.036359	3.73E-09	1.27E-05	10.71803	0.70419	0.756194	0.052004	22	21949277	F	II	UBE2L3	Body	opensea	Body-opensea		rs1135130	49;50
cg0524224	-0.061772	0.401371	-9.031843	3.76E-09	1.28E-05	10.70854	0.370485	0.432257	0.061772	12	53553086	R	II	CSAD	Body	opensea	Body-opensea		rs5564804	23;22
cg0652000	-0.043566	0.128892	-9.020744	3.85E-09	1.30E-05	10.6852	0.107109	0.150675	0.043566	17	48504157	R	II	ACSF2	Body	shore	Body-shore	chr17:4850	rs1155351	47;32;29;27
cg0660382	-0.063886	0.742663	-9.020368	3.85E-09	1.30E-05	10.68441	0.71072	0.774606	0.063886	7	985538	R	II	ADAP1	TSS200	shore	TSS200-sh(	chr7:98561	rs1174859	33
cg1676508	-0.069159	0.488077	-9.017594	3.87E-09	1.30E-05	10.67857	0.453498	0.522656	0.069159	15	99640471	R	II		IGR	shore	IGR-shore	chr15:99640592-99641026		
cg1831163	-0.045137	0.232179	-9.010492	3.93E-09	1.31E-05	10.66362	0.20961	0.254747	0.045137	2	1.55E+08	R	II	GALNT13	3'UTR	opensea	3'UTR-opensea		rs1488791	6
cg1182349	-0.053707	0.159108	-8.993903	4.07E-09	1.35E-05	10.62868	0.132255	0.185962	0.053707	8	23553798	F	II		IGR	opensea	IGR-opensea		rs1885654	0
cg2447040	-0.081339	0.432395	-8.985795	4.13E-09	1.36E-05	10.61159	0.391726	0.473065	0.081339	14	52733119	R	II	PTGDR	TSS1500	shore	TSS1500-sł	chr14:52734207-52735486		
cg1503019	-0.064324	0.762698	-8.980739	4.18E-09	1.36E-05	10.60093	0.730536	0.794859	0.064324	1	2.1E+08	R	II		IGR	opensea	IGR-opensea		rs5716622	2
cg1987040	-0.070901	0.778241	-8.978583	4.20E-09	1.36E-05	10.59638	0.742791	0.813692	0.070901	12	1.26E+08	R	II	TMEM132B	Body	opensea	Body-opensea		rs1231909	30;9;1
cg1700160	-0.069257	0.377433	-8.978246	4.20E-09	1.36E-05	10.59567	0.342805	0.412062	0.069257	3	1.42E+08	R	II	TFDP2	Body	opensea	Body-opensea		rs1156926	41
cg2143654	-0.058811	0.43131	-8.976754	4.21E-09	1.36E-05	10.59252	0.401904	0.460715	0.058811	1	95249289	R	II		IGR	opensea	IGR-opensea			
cg0888853	-0.059475	0.772365	-8.975929	4.22E-09	1.36E-05	10.59078	0.742628	0.802103	0.059475	14	75421063	F	II	PGF	Body	shore	Body-shore	chr14:7542	rs5555830	0;32
cg0193071	-0.065724	0.259539	-8.975823	4.22E-09	1.36E-05	10.59056	0.226677	0.292401	0.065724	16	67575562	F	II	FAM65A	Body	shelf	Body-shelf	chr16:6757	rs2017791	28
cg1302075	-0.080698	0.574641	-8.969572	4.28E-09	1.37E-05	10.57737	0.534292	0.61499	0.080698	13	34214374	R	II	STARD13	5'UTR	opensea	5'UTR-opensea		rs5274574	2
cg0515147	-0.079213	0.725211	-8.966656	4.30E-09	1.37E-05	10.57121	0.685605	0.764818	0.079213	9	1.4E+08	R	II	LCN12	Body	shore	Body-shore	chr9:13984	rs5416007	16
cg0529660	-0.049236	0.683896	-8.960102	4.36E-09	1.39E-05	10.55737	0.659278	0.708514	0.049236	10	1.35E+08	F	I	INPP5A	Body	shore	Body-shore	chr10:1345	rs5580397	13;15;27;48
cg2034019	-0.055668	0.461326	-8.946142	4.49E-09	1.42E-05	10.52788	0.433492	0.48916	0.055668	17	27950413	F	II		IGR	shore	IGR-shore	chr17:27949429-27950277		
cg0336862	-0.067518	0.503302	-8.943084	4.52E-09	1.42E-05	10.52142	0.469543	0.537061	0.067518	7	1.22E+08	R	II	CADPS2	Body	opensea	Body-opensea		rs5656980	27;25
cg2668535	0.024242	0.101612	8.934332	4.60E-09	1.44E-05	10.50291	0.113733	0.089491	-0.024242	4	48485328	R	I	SLC10A4	TSS200	shore	TSS200-sh(	chr4:48485	rs3723385	7
cg1318828	-0.084858	0.650727	-8.934058	4.60E-09	1.44E-05	10.50233	0.608298	0.693156	0.084858	18	12727080	F	II		IGR	opensea	IGR-opensea		rs5722218	10;46;51
cg1943488	-0.075024	0.61324	-8.926315	4.68E-09	1.46E-05	10.48595	0.575728	0.650752	0.075024	15	69026273	F	II		IGR	opensea	IGR-opensea		rs5768736	0;18;21;41;44
cg1038581	-0.090036	0.713116	-8.912502	4.81E-09	1.49E-05	10.4567	0.668098	0.758134	0.090036	2	2734895	F	II		IGR	opensea	IGR-opensea		rs1929198	42
cg1514372	-0.053356	0.229675	-8.912043	4.82E-09	1.49E-05	10.45573	0.202997	0.256354	0.053356	4	1.52E+08	F	II	SH3D19	Body	opensea	Body-opensea		rs5622255	8
cg0404871	-0.046348	0.822222	-8.910584	4.83E-09	1.49E-05	10.45264	0.799048	0.845396	0.046348	17	37873266	F	II	ERBB2	Body	opensea	Body-opensea		rs5653201	46
cg1861939	-0.063434	0.419189	-8.907859	4.86E-09	1.49E-05	10.44687	0.387472	0.450907	0.063434	20	57416506	F	II	GNASAS	Body	island	Body-islanc	chr20:5741	rs5287630	0;3;35
cg1649581	-0.07709	0.661441	-8.904261	4.90E-09	1.49E-05	10.43924	0.622897	0.699986	0.07709	11	73306367	R	II	FAM168A	5'UTR	shelf	5'UTR-shelf	chr11:7330	rs5773024	8
cg2533087	-0.030363	0.914389	-8.892144	5.02E-09	1.52E-05	10.41356	0.899207	0.929571	0.030363	17	17379109	R	II	MED9	TSS1500	shore	TSS1500-sł	chr17:1738	rs5528202	23;2
cg2280243	-0.104965	0.834422	-8.890999	5.03E-09	1.52E-05	10.41113	0.781939	0.886904	0.104965	19	36157825	R	II	UPK1A	Body	opensea	Body-opensea		rs5536370	22;19;13;1
cg1400715	-0.050739	0.855463	-8.886869	5.08E-09	1.53E-05	10.40237	0.830093	0.880832	0.050739	9	1.38E+08	R	II		IGR	shelf	IGR-shelf	chr9:13806	rs5491868	30;27;22
cg2689998	-0.105004	0.712386	-8.885531	5.09E-09	1.53E-05	10.39953	0.659884	0.764888	0.105004	11	1.28E+08	R	II		IGR	shore	IGR-shore	chr11:1283	rs5369394	32
cg1685967	0.022505	0.048119	8.883446	5.11E-09	1.53E-05	10.39511	0.059372	0.036866	-0.022505	9	1.31E+08	R	I	CIZ1	TSS200	island	TSS200-isla	chr9:13095	rs1098791	33
cg2232328	-0.041071	0.553898	-8.878364	5.17E-09	1.53E-05	10.38432	0.533362	0.574433	0.041071	17	48775390	F	II	ANKRD40	Body	opensea	Body-opensea		rs8081077;	0;10
cg0397803	-0.054668	0.819733	-8.87707	5.18E-09	1.53E-05	10.38157	0.792399	0.847067	0.054668	16	57789046	F	II	KATNB1	ExonBnd	opensea	ExonBnd-opensea		rs1505166	7;12;29;31;42;45;46
cg2621025	-0.073152	0.403732	-8.875926	5.19E-09	1.53E-05	10.37914	0.367156	0.440308	0.073152	2	26863503	F	II	CIB4	Body	opensea	Body-opensea		rs1140106	0
cg1805042	-0.06336	0.108518	-8.875232	5.20E-09	1.53E-05	10.37767	0.076838	0.140198	0.06336	18	5455865	R	II	EPB41L3	Body	opensea	Body-opensea			
cg2392569	-0.054554	0.716725	-8.87289	5.23E-09	1.53E-05	10.3727	0.689448	0.744002	0.054554	17	32854559	R	II		IGR	opensea	IGR-opensea		rs1165533	33
cg2049647	-0.071107	0.834172	-8.871963	5.24E-09	1.53E-05	10.37073	0.798618	0.869726	0.071107	7	1.42E+08	R	II	MOXD2P	Body	opensea	Body-opensea		rs5400914	40;39;4;2
cg2014690	-0.059091	0.66938	-8.861628	5.35E-09	1.53E-05	10.34878	0.639834	0.698925	0.059091	1	90289611	F	II	LRRC8D	5'UTR	shelf	5'UTR-shelf	chr1:90286	rs1420097	24;46
cg2429259	-0.075229	0.221499	-8.857273	5.40E-09	1.53E-05	10.33952	0.183885	0.259114	0.075229	18	34268862	R	II	FHOD3	Body	opensea	Body-opensea		rs7715068	46;36;19
cg0571056	-0.027773	0.141155	-8.857207	5.40E-09	1.53E-05	10.33938	0.127268	0.155041	0.027773	3	13323633	F	II		IGR	island	IGR-island	chr3:13323	rs5362842	7
cg2090913	-0.082388	0.413562	-8.853638	5.44E-09	1.53E-05	10.3318	0.372368	0.454756	0.082388	18	57829591	F	II		IGR	opensea	IGR-opensea			
cg0366643	-0.051531	0.797731	-8.848125	5.50E-09	1.53E-05	10.32007	0.771965	0.823496	0.051531	14	1E+08	R	II		IGR	opensea	IGR-opensea		rs7408443	40;9;2
cg2434980	-0.027195	0.896261	-8.847522	5.51E-09	1.53E-05	10.31879	0.882664	0.909858	0.027195	17	79400831	R	II	BAHCC1	Body	opensea	Body-opensea		rs5679315	50;26;25;18
cg2394101	-0.068231	0.699459	-8.846464	5.52E-09	1.53E-05	10.31654	0.665343	0.733575	0.068231	15	78315944	R	II	TBC1D2B	Body	opensea	Body-opensea			
cg1290362	-0.08544	0.532366	-8.846274	5.52E-09	1.53E-05	10.31614	0.489646	0.575086	0.08544	4	80274268	F	II		IGR	opensea	IGR-opensea		rs1112050	1;44;51
cg2717371	-0.07186	0.28171	-8.846076	5.53E-09	1.53E-05	10.31572	0.245779	0.31764	0.07186	1	40420503	R	II	MFSD2A	TSS1500	island	TSS1500-is	chr1:40420	rs5538195	42
cg1536018	-0.082291	0.334276	-8.845781	5.53E-09	1.53E-05	10.31509	0.29313	0.375421	0.082291	3	1.44E+08	R	II	SLC9A9	TSS200	opensea	TSS200-opensea			
cg1854702	-0.081184	0.466564	-8.845744	5.53E-09	1.53E-05	10.31501	0.425972	0.507156	0.081184	9	84517907	R	II		IGR	opensea	IGR-opensea		rs5586933	45;37;2
cg1628458	-0.054307	0.343795	-8.845097	5.54E-09	1.53E-05	10.31363	0.316642	0.370949	0.054307	12	1.22E+08	R	II	KDM2B	Body	opensea	Body-opensea		rs1808937	1;21
cg1180958	-0.054159	0.135542	-8.844572	5.54E-09	1.53E-05	10.31252	0.108463	0.162622	0.054159	2	85698187	R	II		IGR	opensea	IGR-opensea		rs5410731	43;42;2
cg0235691	-0.065126	0.7783	-8.844092	5.55E-09	1.53E-05	10.3115	0.745737	0.810863	0.065126	3	1.94E+08	R	II		IGR	opensea	IGR-opensea		rs5333721	33;18;4;1
cg2130303	-0.063972	0.50422	-8.842468	5.57E-09	1.53E-05	10.30804	0.472234	0.536206	0.063972	2	1.5E+08	R	II	KIF5C	Body	opensea	Body-opensea		rs5767683	1
cg0795264	-0.058793	0.439947	-8.840079	5.60E-09	1.54E-05	10.30296	0.410551	0.469343	0.058793	22	38812201	R	II		IGR	shelf	IGR-shelf	chr22:3880	rs5607606	31;21;5;2;1
cg0744259	-0.06732	0.233636	-8.836243	5.64E-09	1.54E-05	10.2948	0.199976	0.267296	0.06732	12	1.02E+08	R	II		IGR	opensea	IGR-opensea		rs1442692	41;6
cg2162273	-0.05434	0.40566	-8.832586	5.68E-09	1.55E-05	10.28701	0.37849	0.43283	0.05434	19	35899453	F	II	LINC01531	Body	opensea	Body-opensea		rs5542474	0;14;26;32;33
cg1047899	-0.042661	0.47737	-8.830792	5.71E-09	1.55E-05	10.28319	0.45604	0.498701	0.042661	20	49121625	F	II		IGR	opensea	IGR-opensea		rs1898144	1
cg0368904	-0.03839	0.171718	-8.829016	5.73E-09	1.55E-05	10.27941	0.152524	0.190913	0.03839	6	31619544	R	II	BAT3	5'UTR	shore	5'UTR-shor	chr6:31619	rs1463223	33;11;5;4
cg1984831	-0.04573	0.842846	-8.828325	5.73E-09	1.55E-05	10.27794	0.819981	0.865711	0.04573	17	76397583	F	II	PGS1	Body	shore	Body-shore	chr17:76395406-76395617		
cg2756713	-0.057729	0.630554	-8.818081	5.86E-09	1.57E-05	10.25612	0.60169	0.659419	0.057729	17	53095518	F	II	STXBP4	Body	opensea	Body-opensea		rs1164199	1;9;50
cg2514810	-0.055738	0.644572	-8.810127	5.96E-09	1.59E-05	10.23917	0.616703	0.672441	0.055738	10	17291940	F	II		IGR	opensea	IGR-opensea		rs5431845	0
cg0890163	-0.13166	0.519247	-8.806846	6.00E-09	1.60E-05	10.23218	0.453417	0.585077	0.13166	16	34786998	R	II		IGR	shore	IGR-shore	chr16:3478	rs5457155	31;25
cg0709047	-0.087472	0.761053	-8.803562	6.04E-09	1.60E-05	10.22518	0.717318	0.804789	0.087472	3	43437351	F	II	ANO10	Body	opensea	Body-opensea		rs1926868	0
cg0890385	-0.062148	0.192486	-8.799738	6.09E-09	1.61E-05	10.21702	0.161412	0.22356	0.062148	1	37056769	R	II		IGR	opensea	IGR-opensea		rs1875641	26;5;2
cg1409029	-0.076662	0.701194	-8.798824	6.10E-09	1.61E-05	10.21507	0.662863	0.739525	0.076662	12	1.11E+08	F	II	MYL2	TSS1500	opensea	TSS1500-opensea		rs5598706	17;32;39
cg0504682	-0.020052	0.956871	-8.797204	6.12E-09	1.61E-05	10.21162	0.946845	0.966897	0.020052	7	1.28E+08	R	II		IGR	opensea	IGR-opensea		rs1424955	11
cg1148165	-0.056889	0.386478	-8.79427	6.16E-09	1.61E-05	10.20536	0.358033	0.414922	0.056889	3	72356439	F	II		IGR	opensea	IGR-opensea		rs1497643	25;43
cg1424661	-0.079136	0.631579	-8.792261	6.18E-09	1.61E-05	10.20107	0.592012	0.671147	0.079136	1	20250242	R	II	PLA2G2E	TSS200	opensea	TSS200-opensea		rs1837306	37;1
cg2394061	-0.06685	0.522954	-8.791155	6.20E-09	1.61E-05	10.19871	0.489528	0.556379	0.06685	15	84115479	F	II	SH3GL3	TSS1500	shore	TSS1500-sł	chr15:8411	rs5373480	2;41
cg0536441	-0.077986	0.704231	-8.788233	6.24E-09	1.62E-05	10.19247	0.665238	0.743224	0.077986	5	1.48E+08	F	II	SH3TC2	Body	opensea	Body-opensea		rs5423238	1;7;9;36;40
cg1264756	-0.061719	0.308957	-8.783357	6.30E-09	1.63E-05	10.18206	0.278097	0.339816	0.061719	9	1.13E+08	R	II	SVEP1	TSS1500	shore	TSS1500-sł	chr9:11334	rs7658611	8
cg2143693	-0.044242	0.829316	-8.780866	6.33E-09	1.63E-05	10.17674	0.807195	0.851437	0.044242	9	1.16E+08	F	II	RNF183	TSS200	opensea	TSS200-opensea		rs1463364	0;1;22
cg1084693	-0.103937	0.514323	-8.777557	6.38E-09	1.63E-05	10.16968	0.462354	0.566291	0.103937	17	78151814	R	II	CARD14	Body	shelf	Body-shelf	chr17:7815	rs1294797	9;6;1
cg0625472	0.02806	0.944372	8.777445	6.38E-09	1.63E-05	10.16944	0.958402	0.930342	-0.02806	2	28550422	R	II	BRE	3'UTR	opensea	3'UTR-opensea		rs1480472	49;37
cg0625229	-0.06243	0.486012	-8.773202	6.44E-09	1.64E-05	10.16037	0.454797	0.517228	0.06243	4	85558346	F	II	CDS1	Body	opensea	Body-opensea			
cg1648864	-0.077577	0.670921	-8.763921	6.56E-09	1.67E-05	10.14054	0.632132	0.709709	0.077577	16	11498611	F	II		IGR	opensea	IGR-opensea		rs5704926	0;44;50
cg2280253	-0.039402	0.084317	-8.759467	6.62E-09	1.68E-05	10.13102	0.064616	0.104018	0.039402	8	21777002	R	II	XPO7	TSS200	island	TSS200-isla	chr8:21776842-21777942		
cg0048723	-0.070426	0.116477	-8.753637	6.70E-09	1.69E-05	10.11855	0.081264	0.15169	0.070426	18	32847642	F	II	ZNF397OS	5'UTR	island	5'UTR-islan	chr18:3284	rs5384783	17;31
cg1750020	-0.059773	0.78184	-8.75232	6.72E-09	1.69E-05	10.11573	0.751953	0.811726	0.059773	2	2.42E+08	F	II	KIF1A	Body	island	Body-islanc	chr2:24172	rs5768675	9;17;45
cg1744093	-0.060706	0.776885	-8.749196	6.77E-09	1.69E-05	10.10905	0.746531	0.807238	0.060706	5	1.13E+08	F	II	TSSK1B	3'UTR	opensea	3'UTR-opensea		rs5281032	0;29;35
cg1696866	-0.048009	0.804408	-8.748046	6.78E-09	1.69E-05	10.10659	0.780403	0.828413	0.048009	1	897308	R	II	KLHL17	Body	island	Body-islanc	chr1:89431	rs2022223	49;24;11;2
cg2661374	-0.046204	0.692947	-8.746469	6.81E-09	1.69E-05	10.10321	0.669845	0.716049	0.046204	19	14225000	R	II	PRKACA	TSS200	shelf	TSS200-she	chr19:1422	rs2022322	39;36;32;26;9;8;1
cg1020348	-0.057215	0.466062	-8.741685	6.87E-09	1.71E-05	10.09297	0.437455	0.49467	0.057215	2	85646239	R	II		IGR	shore	IGR-shore	chr2:85645	rs5305342	12
cg1020028	-0.077524	0.652051	-8.732312	7.01E-09	1.73E-05	10.0729	0.613289	0.690814	0.077524	5	3695937	F	II		IGR	opensea	IGR-opensea		rs5510136	9;32;50

cg2346674	-0.082343	0.422773	-8.730561	7.04E-09	1.73E-05	10.06915	0.381602	0.463945	0.082343	6	1.01E+08	F	II		IGR	shelf	IGR-shelf	chr6:10091	rs5674672	0;27;35;49
cg0185178	-0.071167	0.322349	-8.724754	7.12E-09	1.75E-05	10.05671	0.286766	0.357932	0.071167	16	49442215	F	II		IGR	shore	IGR-shore	chr16:49440973-49441301		
cg2559291	-0.076688	0.454128	-8.722385	7.16E-09	1.75E-05	10.05163	0.415784	0.492472	0.076688	10	56561124	R	II	PCDH15	TSS200	opensea	TSS200-opensea			
cg0258218	0.03404	0.037791	8.715206	7.27E-09	1.77E-05	10.03624	0.054812	0.020771	-0.03404	11	695543	R	I	TMEM80	TSS200	island	TSS200-isla	chr11:6947	rs5487793	48;36;30
cg0363982	-0.046654	0.092258	-8.713322	7.30E-09	1.77E-05	10.0322	0.068931	0.115585	0.046654	18	5455802	F	II	EPB41L3	Body	opensea	Body-opensea			
cg1811824	-0.041678	0.885368	-8.711943	7.32E-09	1.77E-05	10.02925	0.864529	0.906207	0.041678	11	75873027	R	II		IGR	shelf	IGR-shelf	chr11:75875624-75876562		
cg2411220	-0.037128	0.058305	-8.707697	7.38E-09	1.78E-05	10.02014	0.039741	0.076869	0.037128	3	28390729	R	I	AZI2	TSS200	island	TSS200-isla	chr3:28389	rs5606036	30;24;23;22
cg1196181	-0.062982	0.298056	-8.706986	7.39E-09	1.78E-05	10.01861	0.266566	0.329547	0.062982	17	40995546	F	II	PSME3	3'UTR	opensea	3'UTR-opensea		rs5548612	23;35
cg0737691	-0.035232	0.086996	-8.706439	7.40E-09	1.78E-05	10.01744	0.069379	0.104612	0.035232	20	15986081	F	II	MACROD2	Body	opensea	Body-opensea		rs5293080	0
cg1184060	-0.060036	0.474043	-8.70106	7.49E-09	1.79E-05	10.0059	0.444025	0.504061	0.060036	11	95569370	F	II	MTMR2	Body	opensea	Body-opensea		rs1386265	11;18;45
cg2469888	-0.049702	0.503952	-8.700762	7.49E-09	1.79E-05	10.00526	0.479101	0.528803	0.049702	6	34110624	F	II	GRM4	5'UTR	shore	5'UTR-shor	chr6:34111	rs5432470	1;2;28;33
cg1547485	-0.034065	0.292567	-8.69824	7.53E-09	1.79E-05	9.999849	0.275534	0.3096	0.034065	2	2.08E+08	R	II	KLF7	1stExon	shore	1stExon-sh	chr2:20803	rs3708710	6
cg2347916	-0.040882	0.140929	-8.69544	7.58E-09	1.80E-05	9.993838	0.120488	0.16137	0.040882	16	74896810	R	II		IGR	opensea	IGR-opensea		rs5742289	19
cg2519168	-0.064477	0.617237	-8.682838	7.78E-09	1.82E-05	9.966775	0.584999	0.649475	0.064477	15	41206767	R	II		IGR	opensea	IGR-opensea		rs5705735	4
cg1455527	-0.068255	0.399038	-8.681171	7.81E-09	1.82E-05	9.963193	0.36491	0.433165	0.068255	18	57621101	R	II		IGR	opensea	IGR-opensea		rs7228771	28;13
cg1836903	-0.09375	0.782226	-8.679718	7.83E-09	1.82E-05	9.96007	0.735351	0.829101	0.09375	14	52733216	R	II	PTGDR	TSS1500	shore	TSS1500-sh	chr14:5273	rs5369549	38;15
cg0266168	-0.058062	0.466548	-8.679185	7.84E-09	1.82E-05	9.958926	0.437516	0.495579	0.058062	9	1.31E+08	R	II	ST6GALNA	Body	shore	Body-shore	chr9:130679120-130679612		
cg1499780	-0.069167	0.389682	-8.678463	7.85E-09	1.82E-05	9.957374	0.355098	0.424265	0.069167	19	30270939	R	II		IGR	opensea	IGR-opensea		rs3745289	50;7;1
cg1608172	-0.051125	0.633781	-8.677965	7.86E-09	1.82E-05	9.956303	0.608218	0.659343	0.051125	3	1.27E+08	F	II	PLXNA1	Body	shelf	Body-shelf	chr3:12674	rs5775205	0
cg1877210	-0.055635	0.835283	-8.677675	7.87E-09	1.82E-05	9.955681	0.807465	0.8631	0.055635	5	6348914	R	II		IGR	opensea	IGR-opensea		rs5692102	39;33;17
cg1839855	-0.040874	0.907884	-8.676911	7.88E-09	1.82E-05	9.954037	0.887447	0.928321	0.040874	1	41282523	F	II	KCNQ4	Body	shore	Body-shore	chr1:41283	rs1387903	44;46;50
cg0642524	0.028883	0.12485	8.674224	7.92E-09	1.82E-05	9.948261	0.139291	0.110408	-0.028883	10	21462932	R	I	NEBL-AS1	TSS1500	island	TSS1500-is	chr10:2146	rs5687185	13;11
cg2760026	-0.070999	0.614276	-8.673604	7.93E-09	1.82E-05	9.94693	0.578777	0.649776	0.070999	7	1.5E+08	R	II		IGR	opensea	IGR-opensea		rs7372613	35;18;3
cg0180887	-0.06603	0.133723	-8.67229	7.96E-09	1.82E-05	9.944106	0.100708	0.166738	0.06603	12	98911336	F	II	LOC10012	TSS1500	shore	TSS1500-sh	chr12:9890	rs1388719	0;6;44;46
cg2356075	-0.062675	0.643714	-8.669033	8.01E-09	1.83E-05	9.937102	0.612376	0.675051	0.062675	2	10227754	R	II		IGR	opensea	IGR-opensea		rs3755851	1
cg0306236	-0.073695	0.655034	-8.667856	8.03E-09	1.83E-05	9.934571	0.618186	0.691882	0.073695	22	37945869	R	II		IGR	opensea	IGR-opensea			
cg0228308	-0.041301	0.118492	-8.664433	8.09E-09	1.83E-05	9.92721	0.097841	0.139142	0.041301	14	57273208	F	II	OTX2	Body	shore	Body-shore	chr14:5727	rs5757065	0;7;49
cg0880865	-0.07523	0.770357	-8.660305	8.16E-09	1.83E-05	9.91833	0.732741	0.807972	0.07523	10	1.06E+08	R	II	COL17A1	5'UTR	opensea	5'UTR-opensea		rs5733362	26;7;2
cg1883649	-0.060205	0.056205	-8.659802	8.17E-09	1.83E-05	9.917248	0.026102	0.086308	0.060205	4	55524333	R	I	KIT	Body	island	Body-islanc	chr4:55523	rs999020	31
cg0080826	-0.067429	0.704175	-8.658126	8.20E-09	1.83E-05	9.913641	0.670461	0.73789	0.067429	1	5826065	R	II		IGR	opensea	IGR-opensea		rs1156067	2
cg1138057	-0.081688	0.505863	-8.657729	8.20E-09	1.83E-05	9.912788	0.465019	0.546707	0.081688	6	28271503	F	II		IGR	opensea	IGR-opensea			
cg0944030	-0.071633	0.783613	-8.657438	8.21E-09	1.83E-05	9.912161	0.747796	0.81943	0.071633	12	17224634	F	II		IGR	opensea	IGR-opensea		rs1439206	1;16;44
cg0124581	-0.07301	0.502656	-8.652336	8.30E-09	1.84E-05	9.901181	0.466151	0.539161	0.07301	16	21261658	F	II	ANKS4B	Body	opensea	Body-opensea		rs3765167	0;27
cg2093615	-0.052929	0.796494	-8.652236	8.30E-09	1.84E-05	9.900965	0.77003	0.822958	0.052929	12	6969554	F	II	USP5	Body	opensea	Body-opensea		rs1156120	20;21
cg2283732	-0.055618	0.796958	-8.649323	8.35E-09	1.85E-05	9.894696	0.769149	0.824767	0.055618	16	75283006	F	II	BCAR1	TSS1500	shore	TSS1500-sh	chr16:7528	rs5482324	0;1;44
cg1511162	-0.056934	0.709946	-8.645505	8.42E-09	1.86E-05	9.886475	0.681479	0.738413	0.056934	12	1.33E+08	R	II	FBRSL1	Body	shore	Body-shore	chr12:1331	rs1474348	35;29;13;1
cg0366439	-0.053899	0.727834	-8.644913	8.43E-09	1.86E-05	9.885199	0.700884	0.754783	0.053899	17	75590816	R	II		IGR	opensea	IGR-opensea		rs1168632	6
cg1458269	-0.065992	0.804303	-8.642073	8.48E-09	1.86E-05	9.879084	0.771307	0.837299	0.065992	7	1.58E+08	R	II	PTPRN2	Body	island	Body-islanc	chr7:15769	rs1254063	36
cg2597827	-0.048395	0.159794	-8.6351	8.60E-09	1.87E-05	9.864062	0.135597	0.183992	0.048395	11	15914689	R	II		IGR	opensea	IGR-opensea			
cg2069746	-0.03723	0.26689	-8.633555	8.63E-09	1.87E-05	9.860732	0.248276	0.285505	0.03723	2	2.19E+08	R	II	TNS1	5'UTR	opensea	5'UTR-opensea			
cg1716582	-0.047941	0.782888	-8.63324	8.64E-09	1.87E-05	9.860053	0.758917	0.806859	0.047941	3	45838670	F	II	SLC6A20	TSS1500	shore	TSS1500-sh	chr3:45837	rs9834997	4;28;36;47
cg1990050	-0.047598	0.585282	-8.633033	8.64E-09	1.87E-05	9.859607	0.561483	0.609081	0.047598	19	34744562	R	II	KIAA0355	TSS1500	shore	TSS1500-sh	chr19:3474	rs5559844	28;6
cg1200733	-0.068417	0.507162	-8.632706	8.65E-09	1.87E-05	9.858902	0.472954	0.541371	0.068417	7	97133164	R	II		IGR	opensea	IGR-opensea		rs5683104	32;1
cg1121218	-0.055545	0.181892	-8.632143	8.66E-09	1.87E-05	9.857689	0.15412	0.209665	0.055545	2	2.37E+08	R	II		IGR	opensea	IGR-opensea		rs5482296	25;22
cg0514397	-0.025959	0.909672	-8.626729	8.76E-09	1.88E-05	9.846019	0.896692	0.922651	0.025959	22	30886463	F	II	SEC14L4	Body	opensea	Body-opensea			
cg0705660	-0.053285	0.249577	-8.624835	8.79E-09	1.88E-05	9.841937	0.222934	0.27622	0.053285	19	52035287	F	II	SIGLEC6	TSS1500	opensea	TSS1500-opensea		rs5389443	29
cg0051932	-0.081027	0.502564	-8.624259	8.80E-09	1.88E-05	9.840694	0.462051	0.543078	0.081027	16	75020429	F	II	WDR59	TSS1500	shore	TSS1500-sh	chr16:7501	rs5699076	1;15
cg1896179	-0.030317	0.031524	-8.622401	8.84E-09	1.88E-05	9.836688	0.016365	0.046682	0.030317	12	1.01E+08	F	I	UHRF1BP1	Body	island	Body-islanc	chr12:1005	rs5747751	9
cg0300652	-0.0629	0.396424	-8.619781	8.89E-09	1.89E-05	9.831037	0.364974	0.427874	0.0629	1	1.55E+08	R	II		IGR	shore	IGR-shore	chr1:155043412-155043922		
cg2627159	-0.086136	0.306246	-8.616284	8.95E-09	1.90E-05	9.823495	0.263178	0.349314	0.086136	2	1.78E+08	F	II	NFE2L2	Body	shelf	Body-shelf	chr2:17812	rs7799288	37
cg1139038	-0.051084	0.404243	-8.613153	9.01E-09	1.91E-05	9.816739	0.378701	0.429785	0.051084	1	1.52E+08	R	II	RORC	Body	opensea	Body-opensea			
cg0703325	-0.054884	0.718905	-8.611671	9.04E-09	1.91E-05	9.813542	0.691462	0.746347	0.054884	7	75908694	R	II	SRRM3	Body	shore	Body-shore	chr7:75909	rs5692413	45;2
cg0823879	-0.046758	0.87656	-8.610164	9.07E-09	1.91E-05	9.81029	0.853181	0.899939	0.046758	19	581564	R	II	BSG	Body	island	Body-islanc	chr19:5813	rs2004320	32;18;17
cg0007018	-0.043263	0.911615	-8.60621	9.15E-09	1.92E-05	9.801757	0.889984	0.933247	0.043263	9	36765141	R	II		IGR	opensea	IGR-opensea		rs1862380	24;1
cg0652553	-0.042519	0.833662	-8.60056	9.26E-09	1.93E-05	9.789558	0.812403	0.854921	0.042519	9	1.13E+08	F	II	C9orf152	TSS200	opensea	TSS200-opensea		rs5430351	24;51
cg1651617	-0.050435	0.819517	-8.600413	9.26E-09	1.93E-05	9.789241	0.794299	0.844734	0.050435	3	1.38E+08	R	II		IGR	opensea	IGR-opensea		rs5332273	49;2;1
cg0823419	-0.04208	0.792827	-8.598381	9.30E-09	1.93E-05	9.784853	0.771787	0.813867	0.04208	22	50297645	F	II	ALG12	Body	opensea	Body-opensea		rs1506147	9;51
cg1365981	-0.054727	0.514371	-8.595505	9.36E-09	1.93E-05	9.778641	0.487007	0.541735	0.054727	19	39322968	F	II	ECH1	TSS1500	shore	TSS1500-sh	chr19:3932	rs1441040	1;51
cg2614550	-0.065949	0.542535	-8.591874	9.43E-09	1.93E-05	9.770796	0.50956	0.575509	0.065949	11	1.16E+08	F	II		IGR	opensea	IGR-opensea			
cg1415946	-0.055625	0.558614	-8.591692	9.43E-09	1.93E-05	9.770403	0.530802	0.586427	0.055625	1	31703444	R	II	NKAIN1	Body	opensea	Body-opensea		rs7516723	33
cg2680174	-0.040686	0.888282	-8.58929	9.48E-09	1.93E-05	9.765213	0.867939	0.908625	0.040686	16	12252833	F	II	SNX29	Body	opensea	Body-opensea		rs1413599	0;1;2
cg0166973	-0.045063	0.874136	-8.589104	9.48E-09	1.93E-05	9.764811	0.851604	0.896667	0.045063	19	14074320	R	II	RFX1	Body	island	Body-islanc	chr19:1407	rs1132168	25
cg1118646	-0.064724	0.747038	-8.588562	9.50E-09	1.93E-05	9.76364	0.714676	0.779399	0.064724	19	36110654	F	II	HAUS5	ExonBnd	opensea	ExonBnd-opensea		rs1115999	1
cg0234871	-0.036477	0.901115	-8.588257	9.50E-09	1.93E-05	9.762982	0.882877	0.919354	0.036477	2	46608768	R	II	EPAS1	Body	opensea	Body-opensea		rs2005080	44;5
cg0767436	-0.048093	0.556902	-8.587609	9.51E-09	1.93E-05	9.761582	0.532855	0.580949	0.048093	16	75237833	R	II		IGR	opensea	IGR-opensea		rs5411428	38;35;24;15;11
cg0489173	-0.063372	0.681677	-8.584065	9.59E-09	1.94E-05	9.75392	0.649991	0.713363	0.063372	21	35391280	R	II		IGR	opensea	IGR-opensea		rs1508582	3
cg0007038	-0.044406	0.433795	-8.583748	9.59E-09	1.94E-05	9.753235	0.411592	0.455998	0.044406	2	97354356	F	II	FER1L5	Body	opensea	Body-opensea		rs5622743	2;21;31
cg2437716	-0.057068	0.626984	-8.578728	9.69E-09	1.94E-05	9.742384	0.59845	0.655518	0.057068	1	26202779	R	II		IGR	shore	IGR-shore	chr1:26201	rs5465509	50;1
cg2326696	-0.05565	0.399221	-8.577489	9.72E-09	1.94E-05	9.739705	0.371396	0.427046	0.05565	14	1.05E+08	F	II	ZBTB42	TSS1500	shore	TSS1500-sh	chr14:1052	rs1810271	1
cg0319861	-0.089788	0.749124	-8.576991	9.73E-09	1.94E-05	9.738628	0.70423	0.794018	0.089788	16	14287163	R	II	MKL2	Body	opensea	Body-opensea		rs1886854	37;36;1
cg2478807	-0.044795	0.80572	-8.576188	9.75E-09	1.94E-05	9.736891	0.783323	0.828117	0.044795	8	42364460	R	II	SLC20A2	5'UTR	opensea	5'UTR-opensea		rs7741487	38;35;33
cg0091150	-0.073216	0.150796	-8.575592	9.76E-09	1.94E-05	9.735602	0.114187	0.187404	0.073216	12	62400161	F	II	FAM19A2	5'UTR	opensea	5'UTR-opensea		rs1921421	25;27;28
cg2058565	-0.117879	0.553161	-8.575358	9.76E-09	1.94E-05	9.735096	0.494221	0.612101	0.117879	2	2.34E+08	F	II	INPP5D	Body	opensea	Body-opensea		rs1891894	19;26;46
cg0330070	-0.072723	0.604923	-8.574286	9.79E-09	1.94E-05	9.732778	0.568561	0.641284	0.072723	6	35114523	R	II		IGR	opensea	IGR-opensea			
cg2149162	-0.061628	0.52177	-8.573728	9.80E-09	1.94E-05	9.73157	0.490956	0.552585	0.061628	2	1.22E+08	R	II	GLI2	Body	opensea	Body-opensea		rs5306257	4
cg2710443	-0.080058	0.441621	-8.572085	9.83E-09	1.94E-05	9.728016	0.401592	0.48165	0.080058	10	1.21E+08	R	II	GRK5	Body	opensea	Body-opensea		rs7098836	49;16;14;4;1
cg0639446	-0.137714	0.598393	-8.56997	9.88E-09	1.94E-05	9.723441	0.529536	0.66725	0.137714	13	28130393	R	II	LNX2	Body	opensea	Body-opensea			
cg1115406	-0.053145	0.495799	-8.566663	9.95E-09	1.95E-05	9.716286	0.469227	0.522372	0.053145	15	40379796	F	II		IGR	opensea	IGR-opensea		rs1394725	4;5
cg0659825	-0.046086	0.308174	-8.559077	1.01E-08	1.98E-05	9.699868	0.28513	0.331217	0.046086	1	21521902	R	II		IGR	opensea	IGR-opensea		rs5782594	23
cg0690109	-0.066406	0.673852	-8.558747	1.01E-08	1.98E-05	9.699155	0.640649	0.707055	0.066406	1	34090711	R	II	CSMD2	Body	island	Body-islanc	chr1:34090	rs1997810	48;42;41;34;17
cg2759233	0.100149	0.122806	8.557425	1.01E-08	1.98E-05	9.696291	0.17288	0.072731	-0.100149	18	19756582	R	I	GATA6	Body	shore	Body-shore	chr18:1975	rs7870968	32;28;27
cg2224022	-0.066711	0.755493	-8.555526	1.02E-08	1.98E-05	9.69218	0.722137	0.788849	0.066711	3	1.52E+08	F	II		IGR	opensea	IGR-opensea		rs1162761	32
cg2675000	-0.073531	0.622122	-8.55382	1.02E-08	1.98E-05	9.688486	0.585356	0.658887	0.073531	19	49244674	R	II	IZUMO1	Body	shore	Body-shore	chr19:4924	rs5775024	41;40;16
cg2693120	-0.078192	0.555399	-8.550129	1.03E-08	1.99E-05	9.680493	0.516303	0.594495	0.078192	19	49244592	R	II	IZUMO1	Body	shore	Body-shore	chr19:4924	rs2010618	49;13;1
cg0662524	-0.056198	0.407817	-8.546616	1.04E-08	1.99E-05	9.672883	0.379718	0.435916	0.056198	19	54175187	F	II	MIR1323	TSS200	opensea	TSS200-opensea		rs7944073	1;11
cg0296697	-0.077413	0.306572	-8.546142	1.04E-08	1.99E-05	9.671855	0.267866	0.345279	0.077413	14	51836760	R	II		IGR	opensea	IGR-opensea		rs5349485	11;2
cg1541169	-0.065674	0.678593	-8.544985	1.04E-08	1.99E-05	9.669349	0.645756	0.711431	0.065674	2	2.25E+08	R	II		IGR	opensea	IGR-opensea		rs5773154	18;10
cg1056118	-0.086811	0.394464	-8.544613	1.04E-08	1.99E-05	9.668543	0.351058	0.437869	0.086811	1	30182943	R	II		IGR	opensea	IGR-opensea		rs7287982	48
cg1328447	-0.073452	0.507027	-8.544209	1.04E-08	1.99E-05	9.667668	0.470301	0.543753	0.073452	2	69001115	F	II	ARHGAP25	TSS1500	opensea	TSS1500-opensea		rs5352729	17;24
cg0577706	-0.070324	0.208705	-8.5385	1.06E-08	2.01E-05	9.655294	0.173543	0.243867	0.070324	19	51505001	F	II	KLK8	TSS200	shore	TSS200-sho	chr19:5150	rs7393610	17;45
cg2502740	-0.076296	0.556771	-8.529311	1.08E-08	2.04E-05	9.635373	0.518623	0.594918	0.076296	2	3146960	R	II		IGR	opensea	IGR-opensea		rs5511983	23;12;5;2;1
cg2727220	-0.043475	0.766043	-8.527484	1.08E-08	2.04E-05	9.63141	0.744305	0.787781	0.043475	12	5158794	F	II		IGR	opensea	IGR-opensea		rs5713189	2;10
cg2535546	-0.065701	0.476556	-8.526328	1.08E-08	2.04E-05	9.628902	0.443706	0.509407	0.065701	20	36576591	R	II		IGR	shelf	IGR-shelf	chr20:3657	rs1491698	36;2
cg2016351	-0.087513	0.20163	-8.524679	1.09E-08	2.04E-05	9.625325	0.157874	0.245387	0.087513	4	36401598	R	II		IGR	opensea	IGR-opensea			
cg1663789	-0.042264	0.400853	-8.523954	1.09E-08	2.04E-05	9.623752	0.379721	0.421985	0.042264	19	15159361	F	II	CASP14	TSS1500	opensea	TSS1500-opensea		rs5348261	1;19
cg1329047	-0.05814	0.778624	-8.523135	1.09E-08	2.04E-05	9.621974	0.749554	0.807694	0.05814	15	94010601	F	II		IGR	opensea	IGR-opensea		rs1131542	28
cg1221369	-0.099578	0.736262	-8.521391	1.10E-08	2.04E-05	9.61819	0.686473	0.786051	0.099578	14	64377760	R	II	SYNE2	Body	opensea	Body-opensea		rs2150288	44
cg1412342	-0.056631	0.434349	-8.519942	1.10E-08	2.04E-05	9.615046	0.406033	0.462665	0.056631	14	1.01E+08	F	II	MEG3	Body	island	Body-islanc	chr14:101293427-101294433		
cg2041163	-0.092526	0.649199	-8.518561	1.10E-08	2.04E-05	9.612049	0.602936	0.695462	0.092526	10	1.26E+08	R	II	CHST15	5'UTR	opensea	5'UTR-opensea		rs5272982	33;1
cg2379632	-0.042209	0.880599	-8.518148	1.10E-08	2.04E-05	9.611153	0.859495	0.901704	0.042209	6	1.07E+08	R	II	BEND3	5'UTR	opensea	5'UTR-opensea		rs1411387	32
cg1618249	-0.099255	0.38549	-8.516879	1.11E-08	2.04E-05	9.6084	0.335862	0.435118	0.099255	1	40829600	F	II		IGR	opensea	IGR-opensea		rs7686989	8

cg1523027	-0.02317	0.057533	-8.516216	1.11E-08	2.04E-05	9.606959	0.045948	0.069119	0.02317	5	1.07E+08	F	II	EFNA5	1stExon	island	1stExon-isl	chr5:10700	rs35553	33
cg0600806	-0.061445	0.259099	-8.513531	1.11E-08	2.05E-05	9.601133	0.228377	0.289822	0.061445	11	73358518	F	II	PLEKHB1	TSS200	opensea	TSS200-opensea		rs5364844	36
cg1717209	-0.057637	0.510153	-8.510706	1.12E-08	2.05E-05	9.594999	0.481334	0.538972	0.057637	6	2926650	R	II		IGR	opensea	IGR-opensea		rs5409516	34;1
cg1150355	-0.059658	0.466503	-8.510401	1.12E-08	2.05E-05	9.594335	0.436674	0.496332	0.059658	14	75867017	R	II		IGR	opensea	IGR-opensea		rs5753680	43;1
cg0201112	-0.060424	0.53761	-8.508121	1.13E-08	2.06E-05	9.589385	0.507398	0.567822	0.060424	1	5770367	F	II		IGR	shore	IGR-shore	chr1:57720	rs871339;r	13;37
cg1528976	-0.029954	0.080607	-8.506662	1.13E-08	2.06E-05	9.586217	0.06563	0.095584	0.029954	12	1.09E+08	F	II	CMKLR1	TSS200	opensea	TSS200-opensea		rs5590921	1;36
cg0767138	-0.133628	0.559456	-8.504062	1.14E-08	2.07E-05	9.580571	0.492642	0.62627	0.133628	9	93716207	F	II		IGR	opensea	IGR-opensea		rs1859930	1;22
cg1066634	-0.040455	0.112225	-8.501028	1.14E-08	2.07E-05	9.573979	0.091998	0.132452	0.040455	6	35270553	F	II	DEF6	Body	opensea	Body-opensea		rs9380501	26
cg0148424	-0.043348	0.848828	-8.500169	1.15E-08	2.07E-05	9.572113	0.827154	0.870502	0.043348	21	37795224	F	II		IGR	opensea	IGR-opensea		rs5520254	39
cg1904755	-0.020674	0.926527	-8.499887	1.15E-08	2.07E-05	9.571501	0.91619	0.936864	0.020674	1	36790373	F	II	EVA1B	TSS1500	shore	TSS1500-sl	chr1:36786	rs5468895	1
cg1959952	-0.039261	0.126393	-8.49946	1.15E-08	2.07E-05	9.570573	0.106762	0.146023	0.039261	19	39819001	F	II	GMFG	3'UTR	opensea	3'UTR-opensea		rs5570361	4;12
cg1680354	-0.075313	0.49393	-8.49562	1.16E-08	2.08E-05	9.562229	0.456273	0.531587	0.075313	11	75924614	F	II		IGR	shelf	IGR-shelf	chr11:7591	rs1853305	0
cg0396125	-0.12603	0.444524	-8.491975	1.17E-08	2.09E-05	9.554307	0.381509	0.50754	0.12603	10	1.3E+08	R	II		IGR	opensea	IGR-opensea		rs5670926	31;5;2
cg0945354	-0.043774	0.856587	-8.486381	1.18E-08	2.11E-05	9.542144	0.8347	0.878474	0.043774	17	72447031	F	II		IGR	shelf	IGR-shelf	chr17:72442927-72443194		
cg0489150	-0.047189	0.882123	-8.484677	1.18E-08	2.11E-05	9.538438	0.858529	0.905718	0.047189	8	1.44E+08	F	II		IGR	opensea	IGR-opensea		rs5324115	0;1;48;49
cg1159744	-0.058351	0.781155	-8.481524	1.19E-08	2.12E-05	9.53158	0.751979	0.81033	0.058351	1	1.81E+08	F	II	STX6	Body	opensea	Body-opensea		rs4111520	35
cg2097786	-0.066577	0.568928	-8.475219	1.21E-08	2.15E-05	9.517864	0.53564	0.602217	0.066577	16	71264290	R	II	HYDIN	5'UTR	shore	5'UTR-shor	chr16:7126	rs5409038	42;25;2
cg0156748	-0.048139	0.358016	-8.471694	1.22E-08	2.15E-05	9.510194	0.333946	0.382085	0.048139	5	1.77E+08	F	II	RGS14	TSS1500	opensea	TSS1500-opensea		rs5405169	0;1
cg2143192	-0.078503	0.515301	-8.471011	1.22E-08	2.15E-05	9.508707	0.47605	0.554553	0.078503	4	8536117	F	II		IGR	opensea	IGR-opensea		rs5337095	11;31;34;40;48
cg2015332	-0.058473	0.488863	-8.470449	1.22E-08	2.15E-05	9.507484	0.459626	0.5181	0.058473	12	1.21E+08	R	II	PXN	TSS1500	shore	TSS1500-sl	chr12:120702976-120703541		
cg0795657	-0.049578	0.693508	-8.467927	1.23E-08	2.15E-05	9.501993	0.668719	0.718297	0.049578	3	1.29E+08	R	II	SNORA7B	TSS1500	opensea	TSS1500-opensea		rs5541060	47;43;32;28
cg2010869	-0.033797	0.945096	-8.467801	1.23E-08	2.15E-05	9.50172	0.928198	0.961995	0.033797	12	1.12E+08	R	II	CUX2	Body	opensea	Body-opensea		rs1824174	25
cg1133121	-0.052986	0.758549	-8.466944	1.23E-08	2.15E-05	9.499855	0.732056	0.785042	0.052986	10	80763437	F	II	ZMIZ1-AS1	Body	opensea	Body-opensea		rs5727972	1;4;5
cg1702970	-0.055796	0.450539	-8.466743	1.23E-08	2.15E-05	9.499417	0.422641	0.478437	0.055796	17	17721645	F	II	SREBF1	Body	opensea	Body-opensea		rs1492658	0;1;46
cg0167947	-0.047684	0.77079	-8.464581	1.24E-08	2.15E-05	9.49471	0.746948	0.794633	0.047684	16	1414013	R	II	UNKL	3'UTR	shore	3'UTR-shor	chr16:1414	rs5292067	32;2
cg1135779	-0.063816	0.588697	-8.463639	1.24E-08	2.15E-05	9.492659	0.556789	0.620605	0.063816	12	42959656	F	II	PRICKLE1	5'UTR	opensea	5'UTR-opensea		rs5407376	1
cg1149571	-0.072508	0.186039	-8.462967	1.24E-08	2.15E-05	9.491194	0.149786	0.222293	0.072508	11	1.24E+08	R	II		IGR	opensea	IGR-opensea		rs1512123	28;26
cg0993946	-0.04746	0.392042	-8.462423	1.24E-08	2.15E-05	9.490011	0.368312	0.415772	0.04746	17	25708131	R	II		IGR	opensea	IGR-opensea		rs5503498	38;29;27;19;18;12;9;4
cg0678490	-0.048663	0.768425	-8.460216	1.25E-08	2.15E-05	9.485204	0.744094	0.792757	0.048663	8	840770	R	II		IGR	opensea	IGR-opensea		rs5320076	33;18;5
cg0169208	-0.052882	0.636704	-8.457572	1.25E-08	2.16E-05	9.479444	0.610263	0.663145	0.052882	1	2476749	F	II		IGR	shore	IGR-shore	chr1:24775	rs5499488	0;18;21;30;35
cg2552188	-0.055148	0.498804	-8.454707	1.26E-08	2.17E-05	9.473203	0.47123	0.526378	0.055148	1	2.08E+08	R	II		IGR	shore	IGR-shore	chr1:20804	rs5611386	36
cg2701756	-0.050702	0.641666	-8.449858	1.28E-08	2.19E-05	9.462639	0.616315	0.667017	0.050702	19	35782899	F	II	MAG	TSS200	shelf	TSS200-she	chr19:3578	rs7903278	0;17;25
cg1963163	-0.056487	0.587175	-8.445036	1.29E-08	2.20E-05	9.452127	0.558932	0.615419	0.056487	10	90639349	R	II	STAMBPL1	TSS1500	shore	TSS1500-sl	chr10:9063	rs5288354	26
cg1194655	-0.065813	0.444396	-8.439974	1.30E-08	2.21E-05	9.44109	0.411489	0.477302	0.065813	3	59561166	F	II		IGR	opensea	IGR-opensea			
cg0566030	-0.064674	0.683006	-8.439874	1.30E-08	2.21E-05	9.440873	0.650669	0.715344	0.064674	2	1.59E+08	F	II		IGR	opensea	IGR-opensea		rs5418366	17
cg2274962	-0.039804	0.661458	-8.439439	1.30E-08	2.21E-05	9.439924	0.641556	0.681359	0.039804	11	69062927	R	II	MYEOV	5'UTR	opensea	5'UTR-opensea		rs5435721	50;49;13;1
cg0719933	-0.050104	0.178513	-8.438951	1.31E-08	2.21E-05	9.438861	0.153462	0.203565	0.050104	11	20408536	F	II	PRMT3	TSS1500	shore	TSS1500-sl	chr11:2040	rs5327714	31
cg1392543	-0.051624	0.691045	-8.43747	1.31E-08	2.21E-05	9.435631	0.665233	0.716857	0.051624	14	95702905	F	II	CLMN	Body	opensea	Body-opensea		rs5736465	0;29
cg0959406	-0.037308	0.13378	-8.435517	1.32E-08	2.22E-05	9.431371	0.115126	0.152433	0.037308	7	37956906	F	II	SFRP4	TSS1500	shore	TSS1500-sl	chr7:37955	rs1121177	35;38
cg2253677	-0.043983	0.45031	-8.432877	1.32E-08	2.23E-05	9.425613	0.428318	0.472301	0.043983	1	1851439	R	II	TMEM52	TSS1500	shore	TSS1500-sl	chr1:18500	rs5339050	46;29;24
cg2748329	-0.053225	0.264358	-8.427846	1.34E-08	2.25E-05	9.414635	0.237746	0.290971	0.053225	19	51393769	F	II	KLKP1	Body	opensea	Body-opensea		rs1842558	7;17;23
cg1294608	-0.055736	0.761117	-8.426098	1.34E-08	2.25E-05	9.410819	0.733249	0.788985	0.055736	1	6543581	R	II	PLEKHG5	5'UTR	shore	5'UTR-shor	chr1:65451	rs1919228	29;4
cg0221722	-0.073745	0.31253	-8.425331	1.34E-08	2.25E-05	9.409146	0.275657	0.349403	0.073745	19	2491455	F	II		IGR	shore	IGR-shore	chr19:2489	rs1388495	0;1;46
cg1347388	-0.094161	0.396851	-8.423465	1.35E-08	2.25E-05	9.405074	0.349771	0.443932	0.094161	10	33453307	F	II		IGR	opensea	IGR-opensea			
cg2648603	-0.085072	0.299317	-8.422004	1.35E-08	2.25E-05	9.401884	0.256781	0.341853	0.085072	8	19794619	R	II		IGR	shelf	IGR-shelf	chr8:19796	rs5567559	33
cg0795432	-0.079149	0.631303	-8.419474	1.36E-08	2.26E-05	9.39636	0.591729	0.670878	0.079149	8	1.44E+08	R	II	CYP11B2	TSS200	opensea	TSS200-opensea		rs6175859	42;38
cg1829233	-0.086819	0.152446	-8.418689	1.36E-08	2.26E-05	9.394647	0.109036	0.195855	0.086819	6	1.19E+08	R	II	FAM184A	5'UTR	opensea	5'UTR-opensea		rs5506597	49;40;25;11
cg0173916	-0.060864	0.405586	-8.413124	1.38E-08	2.28E-05	9.382495	0.375154	0.436018	0.060864	17	4806344	F	II	CHRNE	1stExon	shore	1stExon-sh	chr17:4802	rs2011180	8;9;11;22
cg0324251	-0.031575	0.055928	-8.411806	1.38E-08	2.28E-05	9.379616	0.04014	0.071715	0.031575	19	34533035	F	II		IGR	opensea	IGR-opensea		rs5753350	49
cg2607042	-0.06393	0.265565	-8.409447	1.39E-08	2.29E-05	9.374463	0.2336	0.29753	0.06393	10	59388601	R	II		IGR	opensea	IGR-opensea		rs5521164	11;1
cg2429158	-0.061665	0.602301	-8.406825	1.40E-08	2.29E-05	9.368732	0.571468	0.633133	0.061665	8	41699494	R	II	ANK1	Body	opensea	Body-opensea		rs1179496	49
cg2599315	-0.043097	0.888908	-8.406603	1.40E-08	2.29E-05	9.368249	0.867359	0.910456	0.043097	1	2450205	F	II	PANK4	Body	island	Body-islanc	chr1:24499	rs5431763	17;20;39
cg1376691	-0.066718	0.398154	-8.40202	1.41E-08	2.30E-05	9.358234	0.364795	0.431513	0.066718	16	31199666	R	II	FUS	ExonBnd	opensea	ExonBnd-opensea		rs1997457	9
cg1418216	-0.053039	0.514253	-8.401932	1.41E-08	2.30E-05	9.358041	0.487733	0.540772	0.053039	1	40508568	F	II	CAP1	5'UTR	shore	5'UTR-shor	chr1:40505411-40506703		
cg0886629	-0.095172	0.637291	-8.401878	1.41E-08	2.30E-05	9.357923	0.589705	0.684877	0.095172	17	17876835	F	II	TOM1L2	TSS1500	shore	TSS1500-sl	chr17:17875167-17875933		
cg0094051	-0.074148	0.814434	-8.401296	1.42E-08	2.30E-05	9.356651	0.77736	0.851508	0.074148	20	60881152	F	II	ADRM1	Body	shore	Body-shore	chr20:6088	rs5616758	1
cg2513620	-0.058359	0.734541	-8.400116	1.42E-08	2.30E-05	9.354072	0.705361	0.763721	0.058359	3	44928523	F	II	TGM4	Body	opensea	Body-opensea		rs5427996	26
cg1427528	-0.060017	0.616287	-8.399083	1.42E-08	2.30E-05	9.351813	0.586278	0.646296	0.060017	17	43189622	F	II	PLCD3	3'UTR	opensea	3'UTR-opensea		rs5742878	18
cg1687009	-0.027543	0.903941	-8.397975	1.43E-08	2.30E-05	9.34939	0.890169	0.917712	0.027543	20	60587364	R	II	TAF4	Body	island	Body-islanc	chr20:60587264-60587488		
cg0926190	-0.073648	0.311193	-8.397302	1.43E-08	2.30E-05	9.34792	0.274369	0.348017	0.073648	6	1.49E+08	R	II		IGR	opensea	IGR-opensea		rs5505025	21;2
cg0517044	-0.085294	0.572342	-8.395463	1.43E-08	2.30E-05	9.3439	0.529695	0.61499	0.085294	12	1641924	F	II		IGR	shore	IGR-shore	chr12:1639	rs5404998	51
cg2184096	-0.059092	0.717094	-8.394029	1.44E-08	2.30E-05	9.340764	0.687548	0.74664	0.059092	9	1.12E+08	R	II	IKBKAP	5'UTR	shore	5'UTR-shor	chr9:11169	rs1407883	10
cg0655698	-0.075384	0.577151	-8.393022	1.44E-08	2.30E-05	9.338561	0.539459	0.614844	0.075384	3	14452819	R	II	SLC6A6	5'UTR	opensea	5'UTR-opensea		rs5612442	47;42;21
cg0764982	-0.037226	0.877259	-8.391956	1.44E-08	2.30E-05	9.336229	0.858646	0.895872	0.037226	1	11826778	F	II	C1orf167	Body	opensea	Body-opensea		rs5541684	0
cg0946621	-0.061508	0.759602	-8.390066	1.45E-08	2.30E-05	9.332097	0.728848	0.790356	0.061508	6	34068597	R	II	GRM4	Body	opensea	Body-opensea		rs1499031	6
cg1893181	-0.102357	0.522348	-8.389604	1.45E-08	2.30E-05	9.331086	0.471169	0.573526	0.102357	1	36565772	F	II	COL8A2	1stExon	opensea	1stExon-opensea		rs1381824	1;9;41;42;50
cg1414351	-0.083601	0.50822	-8.389106	1.45E-08	2.30E-05	9.329997	0.46642	0.55002	0.083601	9	96080793	R	II	C9orf129	Body	shore	Body-shore	chr9:96080	rs1151157	32;12;2;1
cg0591303	-0.054071	0.640868	-8.384464	1.47E-08	2.32E-05	9.319842	0.613833	0.667904	0.054071	22	32928717	F	II	SYN3	Body	opensea	Body-opensea		rs1480705	10
cg1667740	-0.034577	0.889582	-8.38308	1.47E-08	2.32E-05	9.316812	0.872293	0.90687	0.034577	17	8393322	R	II	MYH10	Body	opensea	Body-opensea			
cg1426980	-0.073869	0.319206	-8.380909	1.48E-08	2.32E-05	9.312061	0.282271	0.35614	0.073869	14	22191888	F	II		IGR	opensea	IGR-opensea		rs5639350	48
cg0188144	-0.06738	0.830645	-8.380109	1.48E-08	2.32E-05	9.310312	0.796955	0.864335	0.06738	4	99407996	R	II	TSPAN5	Body	opensea	Body-opensea			
cg0588194	-0.076117	0.176993	-8.379895	1.48E-08	2.32E-05	9.309843	0.138935	0.215052	0.076117	12	1.24E+08	F	II	C12orf65	TSS1500	shore	TSS1500-sl	chr12:1237	rs5759603	7
cg2561680	-0.057032	0.705617	-8.379816	1.48E-08	2.32E-05	9.309671	0.677101	0.734133	0.057032	17	78177341	R	II	CARD14	Body	opensea	Body-opensea		rs7285365	2
cg1630692	-0.069849	0.429349	-8.375829	1.49E-08	2.34E-05	9.300942	0.394424	0.464273	0.069849	19	54368958	R	II	MYADM	TSS1500	shore	TSS1500-sl	chr19:5436	rs1149461	2
cg1942064	-0.062262	0.308285	-8.372685	1.50E-08	2.35E-05	9.29406	0.277154	0.339416	0.062262	1	1.74E+08	R	II	RABGAP1L	5'UTR	opensea	5'UTR-opensea		rs3761846	37
cg1418582	-0.132036	0.498814	-8.371478	1.51E-08	2.35E-05	9.291418	0.432516	0.564552	0.132036	8	21778583	F	II	XPO7	Body	shore	Body-shore	chr8:21776	rs5426753	25;28;45
cg0086532	-0.063324	0.8551	-8.369413	1.52E-08	2.35E-05	9.286895	0.823438	0.886762	0.063324	10	5769165	R	II	FAM208B	Body	opensea	Body-opensea			
cg0412000	-0.050259	0.591898	-8.367916	1.52E-08	2.35E-05	9.283617	0.566769	0.617028	0.050259	19	46257005	F	II	BHMG1	Body	opensea	Body-opensea		rs1870679	15
cg0672345	-0.048187	0.775442	-8.36743	1.52E-08	2.35E-05	9.282552	0.751349	0.799536	0.048187	20	32253019	R	II	NECAB3	Body	shore	Body-shore	chr20:3225	rs5649234	27
cg1377118	-0.048385	0.733841	-8.364225	1.53E-08	2.36E-05	9.275533	0.709648	0.758033	0.048385	2	1.01E+08	R	II	NPAS2	5'UTR	opensea	5'UTR-opensea		rs5780439	44
cg0845323	-0.06065	0.671303	-8.362965	1.54E-08	2.36E-05	9.272772	0.640978	0.701628	0.06065	8	1.46E+08	R	II	DGAT1	Body	opensea	Body-opensea		rs2016466	46;36;2;1
cg1368337	-0.062662	0.350244	-8.362723	1.54E-08	2.36E-05	9.27224	0.318914	0.381575	0.062662	17	72364767	R	II	GPR142	Body	shelf	Body-shelf	chr17:7236	rs5519640	17;1
cg2156347	-0.069945	0.463893	-8.361817	1.54E-08	2.36E-05	9.270257	0.428921	0.498866	0.069945	2	1.58E+08	R	II	ERMN	TSS1500	opensea	TSS1500-opensea			
cg2034527	-0.058824	0.591064	-8.361384	1.54E-08	2.36E-05	9.269308	0.561652	0.620476	0.058824	4	1.54E+08	F	II	TMEM154	Body	opensea	Body-opensea			
cg2212315	-0.058515	0.48742	-8.360101	1.55E-08	2.36E-05	9.266497	0.458162	0.516677	0.058515	11	72326830	R	II	PDE2A	Body	opensea	Body-opensea		rs1120187	42;37;2
cg1424776	-0.054453	0.611551	-8.360052	1.55E-08	2.36E-05	9.26639	0.584324	0.638777	0.054453	2	74600428	R	II	DCTN1	Body	opensea	Body-opensea		rs1480028	33;14;6;1
cg0253890	-0.084202	0.6491	-8.356288	1.56E-08	2.37E-05	9.258141	0.606999	0.691201	0.084202	1	1.17E+08	R	II	ATP1A1	TSS1500	shore	TSS1500-sl	chr1:11691	rs1919520	51
cg2760093	-0.05208	0.087328	-8.355205	1.56E-08	2.37E-05	9.255766	0.061288	0.113369	0.05208	10	23478530	R	II		IGR	shelf	IGR-shelf	chr10:23480697-23482455		
cg0528966	-0.070937	0.57229	-8.35216	1.57E-08	2.38E-05	9.249091	0.536822	0.607759	0.070937	14	74761114	F	II	ABCD4	Body	opensea	Body-opensea			
cg2701593	-0.025555	0.130039	-8.349233	1.58E-08	2.39E-05	9.242675	0.117262	0.142816	0.025555	16	22012404	F	II	C16orf65	1stExon	opensea	1stExon-opensea		rs7184904	15
cg1274540	0.070333	0.082775	8.349229	1.58E-08	2.39E-05	9.242666	0.117941	0.047608	-0.070333	6	33257713	F	I	PFDN6	Body	shore	Body-shore	chr6:33256539-33256973		
cg0730038	-0.043028	0.793943	-8.346087	1.59E-08	2.40E-05	9.235776	0.772429	0.815457	0.043028	6	1.69E+08	F	II		IGR	shore	IGR-shore	chr6:16852	rs1807085	31;49
cg1611078	-0.062268	0.67161	-8.343234	1.60E-08	2.41E-05	9.229518	0.640476	0.702744	0.062268	7	22602133	F	II		IGR	opensea	IGR-opensea			
cg1082479	-0.054213	0.705815	-8.339729	1.62E-08	2.42E-05	9.221829	0.678708	0.732921	0.054213	1	1.08E+08	R	II	MIR7852	TSS1500	opensea	TSS1500-opensea			
cg2705256	-0.049251	0.737754	-8.335487	1.63E-08	2.44E-05	9.212523	0.713128	0.76238	0.049251	17	27216077	F	I	FLOT2	Body	opensea	Body-opensea		rs5336671	9;16
cg2312094	-0.060447	0.316528	-8.334847	1.63E-08	2.44E-05	9.211117	0.286305	0.346752	0.060447	17	58755752	F	II	BCAS3	5'UTR	shore	5'UTR-shor	chr17:5875	rs1420988	27;37
cg1798987	-0.096089	0.576987	-8.332859	1.64E-08	2.44E-05	9.206755	0.528942	0.625031	0.096089	1	18430354	F	II		IGR	shelf	IGR-shelf	chr1:18434	rs5481442	1;20;25;47;50
cg2192609	-0.065875	0.55302	-8.329902	1.65E-08	2.45E-05	9.200264	0.520082	0.585957	0.065875	8	1.41E+08	F	II	TRAPPC9	Body	island	Body-islanc	chr8:14110	rs1894486	18;21;25;41

Supplemental 3 - Significant DMRs

	CHR	start	end	width	strand	value	area	cluster	indexStart	indexEnd	L	clusterL	p.value	fwer	p.valueArea	fwerArea
DMR_1	chr6	29648161	29649092	931	*	-1.59287	38.22885	358720	112683	112706	24	24	0	0	0	0
DMR_2	chr6	32144978	32146779	1801	*	-0.48156	16.85468	359847	116361	116395	35	35	0	0	0.000397	0.196
DMR_3	chr7	27183133	27185732	2599	*	-0.3358	13.76785	383341	124218	124258	41	41	7.10E-06	0.004	0.000759	0.36
DMR_4	chr20	36148133	36149455	1322	*	-0.32057	12.18149	259421	83970	84007	38	41	7.10E-06	0.004	0.001086	0.452
DMR_5	chr11	14993378	14994989	1611	*	-0.37213	10.41976	69138	21659	21686	28	40	7.10E-06	0.004	0.001711	0.588
DMR_6	chr1	2.06E+08	2.06E+08	1125	*	-1.13996	14.81946	34950	12049	12061	13	13	4.26E-05	0.024	0.000618	0.296
DMR_7	chr1	1.54E+08	1.54E+08	677	*	-0.94611	14.19162	25829	8934	8948	15	15	7.81E-05	0.044	0.00071	0.344
DMR_8	chr9	1.25E+08	1.25E+08	1580	*	-0.91947	11.95308	433080	136083	136095	13	25	0.000177	0.096	0.001157	0.464
DMR_9	chr15	99789622	99790022	400	*	-1.30742	10.45935	152851	49217	49224	8	8	0.000199	0.112	0.001704	0.588
DMR_10	chr15	91473059	91473569	510	*	-0.98276	9.827582	151516	48944	48953	10	10	0.00049	0.256	0.002044	0.644
DMR_11	chr1	2.03E+08	2.03E+08	542	*	-0.83787	9.216555	34051	11834	11844	11	11	0.000809	0.384	0.002342	0.696
DMR_12	chr1	2.37E+08	2.37E+08	1034	*	-0.52568	8.410957	40716	13568	13583	16	21	0.000937	0.404	0.003045	0.772
DMR_13	chr17	33759512	33760971	1459	*	-0.51215	8.194331	178937	58047	58062	16	16	0.000994	0.412	0.003301	0.796
DMR_14	chr2	1.84E+08	1.84E+08	1137	*	-0.49493	7.423951	243562	79615	79629	15	15	0.001143	0.448	0.004273	0.864
DMR_15	chr6	31650735	31651676	941	*	-0.41246	7.836661	359530	114988	115006	19	19	0.000916	0.456	0.003677	0.832
DMR_16	chr13	46425210	46426263	1053	*	-0.57943	7.532557	116258	37950	37962	13	14	0.001498	0.536	0.004124	0.852
DMR_17	chr16	3061835	3062975	1140	*	-0.60741	7.288926	155523	50248	50259	12	12	0.001796	0.628	0.004479	0.88
DMR_18	chr19	41882098	41882741	643	*	-0.58987	7.078436	212458	70022	70033	12	13	0.001995	0.648	0.004898	0.896
DMR_19	chr7	12443478	12444115	637	*	-0.66803	7.348384	381459	123652	123662	11	13	0.00181	0.66	0.004394	0.88

Supplemental 2 - Reactome GSEA

ReactomeID	ReactomePathway	setSize	enrichmentScore	pvalue	p.adjust	rank	leading_edge
R-HSA-2559586	DNA Damage/Telomere Stress Induced Senescence	26	0.805725137	1.83E-17	1.36518E-14	50988	tags=36%, list=42%, signal=21%
R-HSA-5674400	Constitutive Signaling by AKT1 E17K in Cancer	24	0.805960136	1.15801E-16	4.31937E-14	69715	tags=53%, list=57%, signal=23%
R-HSA-5693571	Nonhomologous End-Joining (NHEJ)	27	0.766360595	2.98626E-16	7.42583E-14	27222	tags=21%, list=22%, signal=16%
R-HSA-212676	Dopamine Neurotransmitter Release Cycle	20	0.851900169	7.1866E-16	1.3403E-13	55120	tags=45%, list=45%, signal=25%
R-HSA-114508	Effects of PIP2 hydrolysis	26	0.768915694	1.64689E-15	2.45716E-13	48008	tags=41%, list=39%, signal=25%
R-HSA-418360	Platelet calcium homeostasis	29	0.733026452	3.34521E-15	4.15921E-13	56567	tags=55%, list=46%, signal=30%
R-HSA-3000157	Laminin interactions	28	0.734211769	4.77005E-15	5.08351E-13	61835	tags=57%, list=50%, signal=28%
R-HSA-380972	Energy dependent regulation of mTOR by LKB1-AMPK	27	0.737170038	6.99178E-15	5.94764E-13	69418	tags=48%, list=57%, signal=21%
R-HSA-8941326	RUNX2 regulates bone development	29	0.725220065	7.17544E-15	5.94764E-13	46866	tags=33%, list=38%, signal=20%
R-HSA-9619665	EGR2 and SOX10-mediated initiation of Schwann cell myelination	27	0.728369545	1.38823E-14	1.03562E-12	24267	tags=21%, list=20%, signal=17%
R-HSA-350054	Notch-HLH transcription pathway	24	0.753598117	6.09533E-14	4.13374E-12	52160	tags=47%, list=43%, signal=27%
R-HSA-5218921	VEGFR2 mediated cell proliferation	21	0.786267649	1.79102E-13	1.11342E-11	35477	tags=30%, list=29%, signal=21%
R-HSA-3214815	HDACs deacetylate histones	28	0.69209021	2.41477E-13	1.38571E-11	46374	tags=35%, list=38%, signal=22%
R-HSA-5218920	VEGFR2 mediated vascular permeability	28	0.690887951	2.83171E-13	1.5089E-11	55240	tags=45%, list=45%, signal=25%
R-HSA-210500	Glutamate Neurotransmitter Release Cycle	23	0.742789592	4.37912E-13	2.17788E-11	55469	tags=43%, list=45%, signal=24%
R-HSA-8863795	Downregulation of ERBB2 signaling	28	0.681217096	5.69138E-13	2.65361E-11	41140	tags=38%, list=34%, signal=25%
R-HSA-9013507	NOTCH3 Activation and Transmission of Signal to the Nucleus	25	0.719133739	6.88354E-13	3.02066E-11	73177	tags=55%, list=60%, signal=22%
R-HSA-3371571	HSF1-dependent transactivation	24	0.728172601	7.5272E-13	3.11961E-11	63707	tags=48%, list=52%, signal=23%
R-HSA-2173795	Downregulation of SMAD2/3:SMAD4 transcriptional activity	28	0.671972211	1.29829E-12	4.84261E-11	30504	tags=38%, list=25%, signal=29%
R-HSA-181429	Serotonin Neurotransmitter Release Cycle	16	0.851872386	1.25312E-12	4.84261E-11	59439	tags=49%, list=48%, signal=25%
R-HSA-888590	GABA synthesis, release, reuptake and degradation	19	0.797685813	1.64197E-12	5.83291E-11	49321	tags=48%, list=40%, signal=29%
R-HSA-111931	PKA-mediated phosphorylation of CREB	21	0.756045856	2.28126E-12	7.73555E-11	67152	tags=59%, list=55%, signal=27%
R-HSA-901042	Calnexin/calreticulin cycle	25	0.700257661	3.72727E-12	1.20893E-10	44018	tags=26%, list=36%, signal=17%
R-HSA-9671555	Signaling by PDGFR in disease	20	0.775033924	5.18504E-12	1.61168E-10	40852	tags=37%, list=33%, signal=24%
R-HSA-9006335	Signaling by Erythropoietin	24	0.701852619	5.90883E-12	1.76319E-10	52115	tags=43%, list=42%, signal=25%
R-HSA-9683701	Translation of Structural Proteins	29	0.649801925	6.38159E-12	1.83103E-10	42338	tags=31%, list=35%, signal=20%
R-HSA-381042	PERK regulates gene expression	29	0.647164055	7.71865E-12	2.13264E-10	66930	tags=45%, list=55%, signal=21%
R-HSA-110314	Recognition of DNA damage by PCNA-containing replication comple:	29	0.645651862	8.81262E-12	2.34793E-10	48120	tags=26%, list=39%, signal=16%
R-HSA-389357	CD28 dependent PI3K/Akt signaling	22	0.729421461	1.03321E-11	2.65785E-10	43034	tags=33%, list=35%, signal=22%
R-HSA-1538133	G0 and Early G1	26	0.677218121	1.3101E-11	3.25778E-10	16308	tags=12%, list=13%, signal=11%
R-HSA-400685	Sema4D in semaphorin signaling	24	0.69049058	1.48673E-11	3.57774E-10	49677	tags=40%, list=40%, signal=24%
R-HSA-399956	CRMPs in Sema3A signaling	15	0.855200078	1.56007E-11	3.63691E-10	62821	tags=56%, list=51%, signal=27%
R-HSA-186763	Downstream signal transduction	29	0.636569368	1.9911E-11	4.50109E-10	57596	tags=51%, list=47%, signal=27%
R-HSA-388844	Receptor-type tyrosine-protein phosphatases	16	0.824910605	2.09508E-11	4.59684E-10	42304	tags=35%, list=34%, signal=23%
R-HSA-379724	tRNA Aminoacylation	23	0.694605487	2.27099E-11	4.84046E-10	19995	tags=15%, list=16%, signal=12%
R-HSA-164952	The role of Nef in HIV-1 replication and disease pathogenesis	26	0.667608429	2.70679E-11	5.60906E-10	60700	tags=47%, list=49%, signal=24%
R-HSA-163615	PKA activation	20	0.756039691	2.88196E-11	5.79681E-10	67152	tags=59%, list=55%, signal=27%
R-HSA-427389	ERCC6 (CSB) and EHMT2 (G9a) positively regulate rRNA expression	14	0.849053431	2.9528E-11	5.79681E-10	42401	tags=28%, list=35%, signal=19%
R-HSA-1227990	Signaling by ERBB2 in Cancer	25	0.673344563	3.25037E-11	6.21738E-10	52137	tags=49%, list=43%, signal=28%
R-HSA-9006115	Signaling by NTRK2 (TRKB)	25	0.672378725	3.3791E-11	6.30202E-10	43088	tags=31%, list=35%, signal=20%
R-HSA-180024	DARPP-32 events	26	0.665141942	3.51697E-11	6.39917E-10	34861	tags=30%, list=28%, signal=22%
R-HSA-179409	APC-Cdc20 mediated degradation of Nek2A	25	0.670031474	4.28016E-11	7.60239E-10	23115	tags=19%, list=19%, signal=15%
R-HSA-9674555	Signaling by CSF3 (G-CSF)	28	0.633743073	4.48828E-11	7.78664E-10	64422	tags=55%, list=53%, signal=26%
R-HSA-418885	DCC mediated attractive signaling	14	0.843860321	4.8475E-11	8.21871E-10	43088	tags=41%, list=35%, signal=26%
R-HSA-4090294	SUMOylation of intracellular receptors	28	0.62757361	7.35551E-11	1.21938E-09	43756	tags=35%, list=36%, signal=23%
R-HSA-171319	Telomere Extension By Telomerase	21	0.714342462	7.56958E-11	1.22759E-09	56564	tags=36%, list=46%, signal=20%
R-HSA-202131	Metabolism of nitric oxide: NOS3 activation and regulation	17	0.790224215	8.53515E-11	1.29943E-09	60248	tags=53%, list=49%, signal=27%
R-HSA-198323	AKT phosphorylates targets in the cytosol	14	0.837199253	8.47171E-11	1.29943E-09	69715	tags=52%, list=57%, signal=22%
R-HSA-6804116	TP53 Regulates Transcription of Genes Involved in G1 Cell Cycle Arre	14	0.837199253	8.47171E-11	1.29943E-09	52513	tags=38%, list=43%, signal=22%
R-HSA-9620244	Long-term potentiation	23	0.6745029	9.66979E-11	1.44273E-09	57478	tags=58%, list=47%, signal=31%
R-HSA-389513	CTLA4 inhibitory signaling	21	0.708040331	1.08169E-10	1.58223E-09	43749	tags=39%, list=36%, signal=25%
R-HSA-69273	Cyclin A/B1/B2 associated events during G2/M transition	23	0.671038913	1.22591E-10	1.75871E-09	27799	tags=16%, list=23%, signal=12%
R-HSA-174048	APC/C:Cdc20 mediated degradation of Cyclin B	23	0.670020547	1.32558E-10	1.86582E-09	16850	tags=14%, list=14%, signal=12%
R-HSA-6804758	Regulation of TP53 Activity through Acetylation	26	0.646269166	1.36121E-10	1.88049E-09	43034	tags=34%, list=35%, signal=22%
R-HSA-9664565	Signaling by ERBB2 KD Mutants	24	0.658053065	1.60575E-10	2.13909E-09	52137	tags=49%, list=43%, signal=28%
R-HSA-982772	Growth hormone receptor signaling	21	0.702121172	1.59174E-10	2.13909E-09	33588	tags=33%, list=27%, signal=24%
R-HSA-1250196	SHC1 events in ERBB2 signaling	22	0.698302215	1.66942E-10	2.18489E-09	50604	tags=46%, list=41%, signal=27%
R-HSA-3371568	Attenuation phase	14	0.828018883	1.79376E-10	2.26804E-09	76740	tags=54%, list=63%, signal=20%
R-HSA-399954	Sema3A PAK dependent Axon repulsion	14	0.828018883	1.79376E-10	2.26804E-09	58450	tags=54%, list=48%, signal=28%
R-HSA-2028269	Signaling by Hippo	18	0.764585549	2.77096E-10	3.34764E-09	52296	tags=39%, list=43%, signal=22%
R-HSA-3238698	WNT ligand biogenesis and trafficking	24	0.649796564	2.69319E-10	3.34764E-09	57354	tags=48%, list=47%, signal=25%
R-HSA-8982491	Glycogen metabolism	24	0.649238247	2.78222E-10	3.34764E-09	16471	tags=17%, list=13%, signal=15%
R-HSA-392517	Rap1 signalling	16	0.791489674	3.25505E-10	3.85439E-09	27605	tags=34%, list=23%, signal=27%
R-HSA-525793	Myogenesis	28	0.608032577	3.30771E-10	3.85554E-09	49564	tags=54%, list=40%, signal=32%
R-HSA-9013508	NOTCH3 Intracellular Domain Regulates Transcription	24	0.647935995	3.36091E-10	3.85729E-09	55052	tags=47%, list=45%, signal=26%
R-HSA-9013695	NOTCH4 Intracellular Domain Regulates Transcription	19	0.733008284	3.88927E-10	4.39606E-09	62842	tags=53%, list=51%, signal=26%
R-HSA-399955	SEMA3A-Plexin repulsion signaling by inhibiting Integrin adhesion	13	0.847196178	4.1108E-10	4.5771E-09	62821	tags=59%, list=51%, signal=29%
R-HSA-201556	Signaling by ALK	26	0.627461291	4.77761E-10	5.24132E-09	51755	tags=38%, list=42%, signal=22%
R-HSA-3296482	Defects in vitamin and cofactor metabolism	20	0.714645282	4.85709E-10	5.25129E-09	43198	tags=35%, list=35%, signal=23%
R-HSA-3295583	TRP channels	27	0.601836056	5.97951E-10	6.28269E-09	60875	tags=55%, list=50%, signal=28%
R-HSA-380994	ATF4 activates genes in response to endoplasmic reticulum stress	25	0.632447792	5.93425E-10	6.28269E-09	65675	tags=43%, list=54%, signal=20%
R-HSA-1839117	Signaling by cytosolic FGFR1 fusion mutants	16	0.782406992	6.62454E-10	6.86376E-09	65958	tags=51%, list=54%, signal=24%
R-HSA-9659787	Aberrant regulation of mitotic G1/S transition in cancer due to RB1 c	17	0.762437187	8.6953E-10	8.70342E-09	36934	tags=29%, list=30%, signal=20%
R-HSA-9661069	Defective binding of RB1 mutants to E2F1,(E2F2, E2F3)	17	0.762437187	8.6953E-10	8.70342E-09	36934	tags=29%, list=30%, signal=20%
R-HSA-399719	Trafficking of AMPA receptors	28	0.595604582	8.98343E-10	8.70342E-09	65255	tags=54%, list=53%, signal=25%
R-HSA-399721	Glutamate binding, activation of AMPA receptors and synaptic plasti	28	0.595604582	8.98343E-10	8.70342E-09	65255	tags=54%, list=53%, signal=25%
R-HSA-182971	EGFR downregulation	29	0.588352023	8.93815E-10	8.70342E-09	50604	tags=40%, list=41%, signal=24%
R-HSA-445095	Interaction between L1 and Ankyrins	29	0.586069155	1.02265E-09	9.78073E-09	39255	tags=36%, list=32%, signal=25%
R-HSA-3232118	SUMOylation of transcription factors	19	0.718697493	1.12223E-09	1.04217E-08	85987	tags=63%, list=70%, signal=19%
R-HSA-9669938	Signaling by KIT in disease	20	0.701248541	1.13157E-09	1.04217E-08	57606	tags=50%, list=47%, signal=27%
R-HSA-9670439	Signaling by phosphorylated juxtamembrane, extracellular and kinas	20	0.701248541	1.13157E-09	1.04217E-08	57606	tags=50%, list=47%, signal=27%
R-HSA-901032	ER Quality Control Compartment (ERQC)	20	0.700229113	1.21462E-09	1.10501E-08	44018	tags=28%, list=36%, signal=18%
R-HSA-5635838	Activation of SMO	17	0.758658351	1.23312E-09	1.10832E-08	38660	tags=35%, list=32%, signal=24%
R-HSA-2995383	Initiation of Nuclear Envelope (NE) Reformation	18	0.741477803	1.27243E-09	1.13004E-08	64226	tags=51%, list=52%, signal=25%
R-HSA-110373	Resolution of AP sites via the multiple-nucleotide patch replacemen	25	0.617669568	1.36159E-09	1.19499E-08	47828	tags=30%, list=39%, signal=19%
R-HSA-8941856	RUNX3 regulates NOTCH signaling	13	0.832659294	1.39909E-09	1.21363E-08	52119	tags=43%, list=42%, signal=25%
R-HSA-164378	PKA activation in glucagon signalling	17	0.756021199	1.44603E-09	1.2294E-08	67152	tags=63%, list=55%, signal=28%
R-HSA-5627123	RHO GTPases activate PAKs	22	0.665225327	1.45023E-09	1.2294E-08	55240	tags=42%, list=45%, signal=23%
R-HSA-418990	Adherens junctions interactions	27	0.58476153	1.54155E-09	1.29213E-08	47311	tags=52%, list=39%, signal=32%
R-HSA-6804115	TP53 regulates transcription of additional cell cycle genes whose exp	19	0.712610161	1.70773E-09	1.41552E-08	44584	tags=44%, list=36%, signal=28%
R-HSA-6803204	TP53 Regulates Transcription of Genes Involved in Cytochrome C Rel	19	0.712331045	1.72725E-09	1.41596E-08	55608	tags=41%, list=45%, signal=23%
R-HSA-210744	Regulation of gene expression in late stage (branching morphogenes	15	0.797305368	1.79538E-09	1.45582E-08	52119	tags=40%, list=42%, signal=23%
R-HSA-9617324	Negative regulation of NMDA receptor-mediated neuronal transmiss	21	0.665115922	1.85124E-09	1.48498E-08	57328	tags=57%, list=47%, signal=31%
R-HSA-446353	Cell-extracellular matrix interactions	16	0.764339467	2.07769E-09	1.64889E-08	58269	tags=56%, list=48%, signal=30%
R-HSA-181430	Norepinephrine Neurotransmitter Release Cycle	16	0.764086718	2.11093E-09	1.65764E-08	25149	tags=43%, list=21%, signal=34%
R-HSA-416572	Sema4D induced cell migration and growth-cone collapse	20	0.689792757	2.14914E-09	1.67006E-08	49677	tags=41%, list=40%, signal=24%
R-HSA-162710	Synthesis of glycosylphosphatidylinositol (GPI)	16	0.763079491	2.27714E-09	1.75129E-08	20844	tags=19%, list=17%, signal=16%
R-HSA-445144	Signal transduction by L1	20	0.689358197	2.44124E-09	1.85833E-08	60490	tags=57%, list=49%, signal=29%
R-HSA-1855204	Synthesis of IP3 and IP4 in the cytosol	27	0.577392215	2.61041E-09	1.96704E-08	55240	tags=55%, list=45%, signal=30%
R-HSA-5099900	WNT5A-dependent internalization of FZD4	15	0.790203176	3.00234E-09	2.23974E-08	83864	tags=65%, list=68%, signal=20%
R-HSA-430039	mRNA decay by 5' to 3' exoribonuclease	15	0.789969738	3.03251E-09	2.23985E-08	21791	tags=21%, list=18%, signal=17%
R-HSA-6804759	Regulation of TP53 Activity through Association with Co-factors	14	0.788232144	3.07601E-09	2.24971E-08	56540	tags=47%, list=46%, signal=25%
R-HSA-3296469	Defects in cobalamin (B12) metabolism	12	0.837576943	3.46074E-09	2.49662E-08	24091	tags=26%, list=20%, signal=21%
R-HSA-5654696	Downstream signaling of activated FGFR2	29	0.567848773	3.48055E-09	2.49662E-08	66282	tags=55%, list=54%, signal=25%
R-HSA-5654708	Downstream signaling of activated FGFR3	24	0.613053348	3.59617E-09	2.55499E-08	61955	tags=51%, list=51%, signal=25%
R-HSA-5205685	PINK1-PRKN Mediated Mitophagy	16	0.75544419	3.77502E-09	2.65676E-08	62154	tags=42%, list=51%, signal=21%
R-HSA-9013422	RHOBTB1 GTPase cycle	20	0.68129465	3.90179E-09	2.72031E-08	32271	tags=29%, list=26%, signal=22%
R-HSA-5205647	Mitophagy	22	0.651414201	4.00439E-09	2.76599E-08	76940	tags=56%, list=63%, signal=21%
R-HSA-4641262	Disassembly of the destruction complex and recruitment of AXIN to	29	0.56550448	4.06604E-09	2.78281E-08	78906	tags=60%, list=64%, signal=21%

R-HSA-9034015	Signaling by NTRK3 (TRKC)	16	0.754581867	4.1759E-09	2.83202E-08	43088	tags=36%, list=35%, signal=24%
R-HSA-5357956	TNFR1-induced NFkappaB signaling pathway	25	0.597095612	4.81808E-09	3.2381E-08	67425	tags=54%, list=55%, signal=24%
R-HSA-4839743	Signaling by CTNNB1 phospho-site mutants	14	0.779918628	5.22617E-09	3.33224E-08	50928	tags=33%, list=42%, signal=20%
R-HSA-5339716	Signaling by GSK3beta mutants	14	0.779918628	5.22617E-09	3.33224E-08	50928	tags=33%, list=42%, signal=20%
R-HSA-5358747	CTNNB1 S33 mutants aren't phosphorylated	14	0.779918628	5.22617E-09	3.33224E-08	50928	tags=33%, list=42%, signal=20%
R-HSA-5358749	CTNNB1 S37 mutants aren't phosphorylated	14	0.779918628	5.22617E-09	3.33224E-08	50928	tags=33%, list=42%, signal=20%
R-HSA-5358751	CTNNB1 S45 mutants aren't phosphorylated	14	0.779918628	5.22617E-09	3.33224E-08	50928	tags=33%, list=42%, signal=20%
R-HSA-5358752	CTNNB1 T41 mutants aren't phosphorylated	14	0.779918628	5.22617E-09	3.33224E-08	50928	tags=33%, list=42%, signal=20%
R-HSA-5651801	PCNA-Dependent Long Patch Base Excision Repair	21	0.648774551	5.33596E-09	3.37341E-08	50060	tags=31%, list=41%, signal=18%
R-HSA-264876	Insulin processing	26	0.587258715	5.44273E-09	3.38891E-08	47705	tags=42%, list=39%, signal=26%
R-HSA-373753	Nephrin family interactions	21	0.648571392	5.45133E-09	3.38891E-08	53102	tags=50%, list=43%, signal=28%
R-HSA-2979096	NOTCH2 Activation and Transmission of Signal to the Nucleus	22	0.646748737	5.52985E-09	3.40931E-08	16014	tags=11%, list=13%, signal=10%
R-HSA-8866652	Synthesis of active ubiquitin: roles of E1 and E2 enzymes	26	0.586958501	5.63886E-09	3.44802E-08	42807	tags=33%, list=35%, signal=21%
R-HSA-2691230	Signaling by NOTCH1 HD Domain Mutants in Cancer	15	0.777899206	6.3994E-09	3.84996E-08	62093	tags=46%, list=51%, signal=23%
R-HSA-2691232	Constitutive Signaling by NOTCH1 HD Domain Mutants	15	0.777899206	6.3994E-09	3.84996E-08	62093	tags=46%, list=51%, signal=23%
R-HSA-9705462	Inactivation of CSF3 (G-CSF) signaling	23	0.612830289	6.89861E-09	4.11709E-08	64422	tags=54%, list=53%, signal=26%
R-HSA-3214858	RMTs methylate histone arginines	28	0.566287073	7.41632E-09	4.39093E-08	56389	tags=40%, list=46%, signal=22%
R-HSA-442742	CREB1 phosphorylation through NMDA receptor-mediated activatio	26	0.581025806	7.60016E-09	4.46434E-08	58004	tags=52%, list=47%, signal=28%
R-HSA-8949215	Mitochondrial calcium ion transport	20	0.67091908	7.75824E-09	4.5216E-08	58010	tags=42%, list=47%, signal=22%
R-HSA-141405	Inhibition of the proteolytic activity of APC/C required for the onset	20	0.670004158	7.92598E-09	4.5483E-08	23115	tags=20%, list=19%, signal=16%
R-HSA-141430	Inactivation of APC/C via direct inhibition of the APC/C complex	20	0.670004158	7.92598E-09	4.5483E-08	23115	tags=20%, list=19%, signal=16%
R-HSA-450282	MAPK targets/ Nuclear events mediated by MAP kinases	29	0.552765701	8.303E-09	4.72827E-08	35824	tags=26%, list=29%, signal=19%
R-HSA-5696397	Gap-filling DNA repair synthesis and ligation in GG-NER	25	0.586317958	9.16958E-09	5.1822E-08	47828	tags=29%, list=39%, signal=18%
R-HSA-5654716	Downstream signaling of activated FGFR4	26	0.578067524	9.26727E-09	5.19803E-08	66562	tags=55%, list=54%, signal=25%
R-HSA-9665686	Signaling by ERBB2 TMD/JMD mutants	21	0.639798697	1.0029E-08	5.5833E-08	52137	tags=49%, list=43%, signal=28%
R-HSA-210990	PECAM1 interactions	12	0.817210876	1.02768E-08	5.67886E-08	74607	tags=70%, list=61%, signal=27%
R-HSA-264642	Acetylcholine Neurotransmitter Release Cycle	15	0.769697814	1.04218E-08	5.71665E-08	22184	tags=21%, list=18%, signal=17%
R-HSA-2173788	Downregulation of TGF-beta receptor signaling	25	0.583561732	1.08835E-08	5.92633E-08	64778	tags=54%, list=53%, signal=26%
R-HSA-8940973	RUNX2 regulates osteoblast differentiation	22	0.63555265	1.11143E-08	6.00817E-08	44902	tags=35%, list=37%, signal=22%
R-HSA-983170	Antigen Presentation: Folding, assembly and peptide loading of clas	27	0.547383373	1.27131E-08	6.823E-08	31363	tags=22%, list=26%, signal=17%
R-HSA-438066	Unblocking of NMDA receptors, glutamate binding and activation	20	0.662922174	1.34017E-08	7.14117E-08	57328	tags=55%, list=47%, signal=29%
R-HSA-354192	Integrin signaling	26	0.571641284	1.36986E-08	7.24762E-08	46154	tags=41%, list=38%, signal=26%
R-HSA-877312	Regulation of IFNG signaling	14	0.764523088	1.41147E-08	7.41521E-08	28447	tags=18%, list=23%, signal=14%
R-HSA-1362277	Transcription of E2F targets under negative control by DREAM comp	18	0.706461018	1.42148E-08	7.41553E-08	16308	tags=14%, list=13%, signal=12%
R-HSA-912446	Meiotic recombination	22	0.629997575	1.46356E-08	7.52975E-08	32949	tags=22%, list=27%, signal=16%
R-HSA-5685939	HDR through MMEJ (alt-NHEJ)	11	0.855555374	1.45671E-08	7.52975E-08	20002	tags=24%, list=16%, signal=20%
R-HSA-176407	Conversion from APC/C:Cdc20 to APC/C:Cdh1 in late anaphase	19	0.669998695	1.64021E-08	8.3238E-08	63346	tags=44%, list=52%, signal=21%
R-HSA-176412	Phosphorylation of the APC/C	19	0.669998695	1.64021E-08	8.3238E-08	32391	tags=22%, list=26%, signal=16%
R-HSA-5627117	RHO GTPases Activate ROCKs	19	0.667203104	1.8313E-08	9.23073E-08	44600	tags=37%, list=36%, signal=23%
R-HSA-442982	Ras activation upon Ca2+ influx through NMDA receptor	20	0.658367419	1.84655E-08	9.24513E-08	58004	tags=55%, list=47%, signal=29%
R-HSA-912631	Regulation of signaling by CBL	22	0.625341433	1.88171E-08	9.35837E-08	57606	tags=52%, list=47%, signal=28%
R-HSA-8849932	Synaptic adhesion-like molecules	19	0.666061371	1.89499E-08	9.36202E-08	60982	tags=54%, list=50%, signal=27%
R-HSA-2029485	Role of phospholipids in phagocytosis	20	0.656543252	1.98207E-08	9.72779E-08	61711	tags=57%, list=50%, signal=28%
R-HSA-174414	Processive synthesis on the C-strand of the telomere	18	0.697971693	2.10763E-08	1.02764E-07	44950	tags=28%, list=37%, signal=18%
R-HSA-3928664	Ephrin signaling	17	0.712889259	2.12806E-08	1.03087E-07	60367	tags=49%, list=49%, signal=25%
R-HSA-9665348	Signaling by ERBB2 ECD mutants	15	0.758424704	2.21714E-08	1.06709E-07	50604	tags=46%, list=41%, signal=27%
R-HSA-191273	Cholesterol biosynthesis	23	0.58697524	2.28446E-08	1.09244E-07	61913	tags=42%, list=50%, signal=21%
R-HSA-201451	Signaling by BMP	27	0.53404185	2.71363E-08	1.27319E-07	48012	tags=48%, list=39%, signal=29%
R-HSA-400042	Adrenaline,noradrenaline inhibits insulin secretion	27	0.533604012	2.71363E-08	1.27319E-07	58019	tags=63%, list=47%, signal=33%
R-HSA-170670	Adenylate cyclase inhibitory pathway	14	0.756002707	2.69141E-08	1.27319E-07	67152	tags=66%, list=55%, signal=30%
R-HSA-432142	Platelet sensitization by LDL	17	0.710043538	2.79397E-08	1.30269E-07	72517	tags=54%, list=59%, signal=22%
R-HSA-5625900	RHO GTPases activate CIT	19	0.659710838	3.16892E-08	1.46833E-07	42199	tags=30%, list=34%, signal=20%
R-HSA-5682910	LGI-ADAM interactions	14	0.753238812	3.22483E-08	1.48501E-07	59038	tags=59%, list=48%, signal=31%
R-HSA-203615	eNOS activation	13	0.790198444	3.34994E-08	1.53316E-07	64294	tags=53%, list=52%, signal=25%
R-HSA-5607763	CLEC7A (Dectin-1) induces NFAT activation	13	0.78946426	3.52063E-08	1.60146E-07	56198	tags=51%, list=46%, signal=27%
R-HSA-1606322	ZBP1(DAI) mediated induction of type I IFNs	19	0.657891489	3.68068E-08	1.66412E-07	39552	tags=25%, list=32%, signal=17%
R-HSA-139853	Elevation of cytosolic Ca2+ levels	16	0.718086627	3.77018E-08	1.69431E-07	52987	tags=52%, list=43%, signal=30%
R-HSA-1236382	Constitutive Signaling by Ligand-Responsive EGFR Cancer Variants	19	0.656305018	3.87271E-08	1.71967E-07	50604	tags=46%, list=41%, signal=27%
R-HSA-5637815	Signaling by Ligand-Responsive EGFR Variants in Cancer	19	0.656305018	3.87271E-08	1.71967E-07	50604	tags=46%, list=41%, signal=27%
R-HSA-5689901	Metalloprotease DUBs	15	0.747184214	4.16074E-08	1.83664E-07	40392	tags=29%, list=33%, signal=19%
R-HSA-5210891	Uptake and function of anthrax toxins	13	0.78661927	4.33143E-08	1.88962E-07	27975	tags=30%, list=23%, signal=23%
R-HSA-1358803	Downregulation of ERBB2:ERBB3 signaling	13	0.786403364	4.33143E-08	1.88962E-07	43034	tags=38%, list=35%, signal=25%
R-HSA-164938	Nef-mediates down modulation of cell surface receptors by recruitir	19	0.652501468	4.89688E-08	2.1116E-07	67707	tags=53%, list=55%, signal=24%
R-HSA-69205	G1/S-Specific Transcription	25	0.561097609	4.88113E-08	2.1116E-07	28888	tags=20%, list=24%, signal=16%
R-HSA-9613829	Chaperone Mediated Autophagy	20	0.64052592	4.93706E-08	2.1167E-07	80084	tags=61%, list=65%, signal=21%
R-HSA-9694631	Maturation of nucleoprotein	15	0.742718544	5.09677E-08	2.17268E-07	30320	tags=25%, list=25%, signal=19%
R-HSA-428540	Activation of RAC1	11	0.836608075	5.73832E-08	2.43226E-07	49296	tags=43%, list=40%, signal=26%
R-HSA-167044	Signalling to RAS	20	0.634887115	6.0947E-08	2.56873E-07	39575	tags=35%, list=32%, signal=24%
R-HSA-6807004	Negative regulation of MET activity	20	0.632496823	7.04873E-08	2.95413E-07	50124	tags=45%, list=41%, signal=26%
R-HSA-1839124	FGFR1 mutant receptor activation	29	0.518016874	7.16593E-08	2.98647E-07	65958	tags=54%, list=54%, signal=25%
R-HSA-3229121	Glycogen storage diseases	14	0.738827374	7.45616E-08	3.09016E-07	4949	tags=44%, list=4%, signal=42%
R-HSA-1483191	Synthesis of PC	27	0.518199514	7.52419E-08	3.10113E-07	52016	tags=49%, list=42%, signal=28%
R-HSA-418359	Reduction of cytosolic Ca++ levels	13	0.775327344	7.67642E-08	3.14649E-07	57004	tags=57%, list=46%, signal=31%
R-HSA-1221632	Meiotic synapsis	28	0.526890592	8.60903E-08	3.50947E-07	41020	tags=37%, list=33%, signal=25%
R-HSA-8876384	Listeria monocytogenes entry into host cells	19	0.642391233	8.68425E-08	3.5209E-07	18275	tags=21%, list=15%, signal=18%
R-HSA-9700645	ALK mutants bind TKIs	11	0.829360162	8.74185E-08	3.52509E-07	61878	tags=58%, list=50%, signal=29%
R-HSA-9687136	Aberrant regulation of mitotic exit in cancer due to RB1 defects	18	0.669993233	9.39688E-08	3.76886E-07	38120	tags=28%, list=31%, signal=19%
R-HSA-3322077	Glycogen synthesis	13	0.771470259	9.56756E-08	3.81679E-07	7151	tags=18%, list=6%, signal=17%
R-HSA-3371511	HSF1 activation	11	0.82799863	1.00641E-07	3.99353E-07	76738	tags=54%, list=63%, signal=20%
R-HSA-3000170	Syndecan interactions	26	0.538123391	1.04819E-07	4.13731E-07	60120	tags=61%, list=49%, signal=31%
R-HSA-4839735	Signaling by AXIN mutants	13	0.769717253	1.09466E-07	4.18779E-07	68414	tags=47%, list=56%, signal=21%
R-HSA-4839744	Signaling by APC mutants	13	0.769717253	1.09466E-07	4.18779E-07	68414	tags=47%, list=56%, signal=21%
R-HSA-4839748	Signaling by AMER1 mutants	13	0.769717253	1.09466E-07	4.18779E-07	68414	tags=47%, list=56%, signal=21%
R-HSA-5467337	APC truncation mutants have impaired AXIN binding	13	0.769717253	1.09466E-07	4.18779E-07	68414	tags=47%, list=56%, signal=21%
R-HSA-5467340	AXIN missense mutants destabilize the destruction complex	13	0.769717253	1.09466E-07	4.18779E-07	68414	tags=47%, list=56%, signal=21%
R-HSA-5467348	Truncations of AMER1 destabilize the destruction complex	13	0.769717253	1.09466E-07	4.18779E-07	68414	tags=47%, list=56%, signal=21%
R-HSA-5655332	Signaling by FGFR3 in disease	21	0.595933829	1.10682E-07	4.21269E-07	61955	tags=53%, list=51%, signal=26%
R-HSA-381033	ATF6 (ATF6-alpha) activates chaperones	10	0.834326618	1.12345E-07	4.25426E-07	33854	tags=23%, list=28%, signal=17%
R-HSA-112308	Presynaptic depolarization and calcium channel opening	12	0.774023058	1.25532E-07	4.72964E-07	55758	tags=55%, list=45%, signal=30%
R-HSA-264870	Caspase-mediated cleavage of cytoskeletal proteins	12	0.770722759	1.46579E-07	5.49487E-07	43552	tags=41%, list=36%, signal=26%
R-HSA-6804114	TP53 Regulates Transcription of Genes Involved in G2 Cell Cycle Arre	18	0.659582494	1.53375E-07	5.72088E-07	48124	tags=34%, list=39%, signal=20%
R-HSA-193639	p75NTR signals via NF-kB	15	0.724350194	1.56143E-07	5.79517E-07	36375	tags=26%, list=30%, signal=18%
R-HSA-1963642	PI3K events in ERBB2 signaling	16	0.686482973	1.63257E-07	6.02919E-07	42603	tags=44%, list=35%, signal=29%
R-HSA-379726	Mitochondrial tRNA aminoacylation	17	0.67827878	1.79831E-07	6.60858E-07	48015	tags=33%, list=39%, signal=20%
R-HSA-177504	Retrograde neurotrophin signalling	14	0.724344288	1.81202E-07	6.62632E-07	62950	tags=53%, list=51%, signal=26%
R-HSA-416700	Other semaphorin interactions	18	0.65694861	1.83796E-07	6.68837E-07	30505	tags=33%, list=25%, signal=24%
R-HSA-8866907	Activation of the TFAP2 (AP-2) family of transcription factors	11	0.817293895	1.87512E-07	6.75767E-07	72924	tags=53%, list=59%, signal=22%
R-HSA-5654732	Negative regulation of FGFR3 signaling	28	0.51408861	1.87033E-07	6.75767E-07	61955	tags=47%, list=51%, signal=23%
R-HSA-9027284	Erythropoietin activates RAS	13	0.758879272	1.92296E-07	6.89678E-07	50582	tags=47%, list=41%, signal=28%
R-HSA-210991	Basigin interactions	24	0.54020358	2.03704E-07	7.27096E-07	54892	tags=49%, list=45%, signal=27%
R-HSA-112409	RAF-independent MAPK1/3 activation	22	0.579110441	2.31646E-07	8.22896E-07	71359	tags=51%, list=58%, signal=21%
R-HSA-8983432	Interleukin-15 signaling	13	0.756175909	2.33873E-07	8.22967E-07	66010	tags=56%, list=54%, signal=26%
R-HSA-442720	CREB1 phosphorylation through the activation of Adenylate Cyclase	13	0.755996543	2.33873E-07	8.22967E-07	71405	tags=59%, list=58%, signal=25%
R-HSA-174437	Removal of the Flap Intermediate from the C-strand	16	0.679453676	2.4004E-07	8.40704E-07	44620	tags=26%, list=36%, signal=16%
R-HSA-4641265	Repression of WNT target genes	11	0.814497456	2.4657E-07	8.59537E-07	71804	tags=47%, list=59%, signal=19%
R-HSA-499943	Interconversion of nucleotide di- and triphosphates	27	0.498695633	2.62135E-07	9.09547E-07	51262	tags=37%, list=42%, signal=22%
R-HSA-975577	N-Glycan antennae elongation	15	0.71289502	3.01835E-07	1.04245E-06	35332	tags=43%, list=29%, signal=30%
R-HSA-5654693	FRS-mediated FGFR1 signaling	23	0.546482679	3.08143E-07	1.05693E-06	66282	tags=55%, list=54%, signal=25%
R-HSA-912526	Interleukin receptor SHC signaling	24	0.533088404	3.08862E-07	1.05693E-06	60149	tags=50%, list=49%, signal=26%
R-HSA-416993	Trafficking of GluR2-containing AMPA receptors	15	0.710652904	3.33817E-07	1.13711E-06	34081	tags=26%, list=28%, signal=19%
R-HSA-5654689	PI-3K cascade:FGFR1	21	0.573975873	3.36535E-07	1.14116E-06	64186	tags=52%, list=52%, signal=25%

R-HSA-390918	Peroxisomal lipid metabolism	25	0.524733852	3.40092E-07	1.148E-06	49518	tags=32%, list=40%, signal=19%
R-HSA-9603798	Class I peroxisomal membrane protein import	19	0.608649423	3.45612E-07	1.16138E-06	25220	tags=18%, list=21%, signal=14%
R-HSA-168333	NEP/NS2 Interacts with the Cellular Export Machinery	28	0.505375305	3.87975E-07	1.29789E-06	36525	tags=27%, list=30%, signal=19%
R-HSA-168271	Transport of Ribonucleoproteins into the Host Nucleus	28	0.505229384	4.00036E-07	1.33226E-06	36525	tags=28%, list=30%, signal=20%
R-HSA-210993	Tie2 Signaling	18	0.639939991	4.40739E-07	1.46129E-06	51811	tags=48%, list=42%, signal=28%
R-HSA-111932	CaMK IV-mediated phosphorylation of CREB	12	0.748644572	4.58516E-07	1.51351E-06	57933	tags=50%, list=47%, signal=27%
R-HSA-5654700	FRS-mediated FGFR2 signaling	24	0.527063049	4.62093E-07	1.5186E-06	66282	tags=55%, list=54%, signal=25%
R-HSA-450302	activated TAK1 mediates p38 MAPK activation	20	0.598487134	4.7272E-07	1.54671E-06	61428	tags=49%, list=50%, signal=25%
R-HSA-6804760	Regulation of TP53 Activity through Methylation	18	0.637461374	4.76405E-07	1.55196E-06	21131	tags=11%, list=17%, signal=9%
R-HSA-975576	N-glycan antennae elongation in the medial/trans-Golgi	26	0.510655436	4.94536E-07	1.60402E-06	35332	tags=49%, list=29%, signal=35%
R-HSA-162588	Budding and maturation of HIV virion	28	0.499036996	5.05043E-07	1.63101E-06	45093	tags=41%, list=37%, signal=26%
R-HSA-1433559	Regulation of KIT signaling	16	0.664799633	5.26711E-07	1.69365E-06	50582	tags=46%, list=41%, signal=27%
R-HSA-5250924	B-WICH complex positively regulates rRNA expression	27	0.48854081	5.39788E-07	1.72825E-06	38542	tags=32%, list=31%, signal=22%
R-HSA-418592	ADP signalling through P2Y purinoceptor 1	24	0.523390367	5.63029E-07	1.79495E-06	49609	tags=45%, list=40%, signal=27%
R-HSA-71403	Citric acid cycle (TCA cycle)	20	0.594148984	5.94518E-07	1.87928E-06	37342	tags=32%, list=30%, signal=22%
R-HSA-198753	ERK/MAPK targets	20	0.593902987	5.94518E-07	1.87928E-06	35824	tags=28%, list=29%, signal=20%
R-HSA-1643713	Signaling by EGFR in Cancer	24	0.51872491	6.49647E-07	2.04488E-06	50604	tags=45%, list=41%, signal=26%
R-HSA-73929	Base-Excision Repair, AP Site Formation	16	0.655766632	7.7457E-07	2.42785E-06	56391	tags=40%, list=46%, signal=22%
R-HSA-113510	E2F mediated regulation of DNA replication	19	0.58902503	8.30429E-07	2.59205E-06	30933	tags=26%, list=25%, signal=19%
R-HSA-2559584	Formation of Senescence-Associated Heterochromatin Foci (SAHF)	10	0.799668996	8.6118E-07	2.67683E-06	53631	tags=43%, list=44%, signal=24%
R-HSA-110320	Translesion Synthesis by POLH	18	0.627607685	8.65349E-07	2.67863E-06	48520	tags=30%, list=40%, signal=18%
R-HSA-9614657	FOXO-mediated transcription of cell death genes	15	0.687942449	8.96308E-07	2.763E-06	58888	tags=46%, list=48%, signal=24%
R-HSA-113418	Formation of the Early Elongation Complex	29	0.473173716	9.09511E-07	2.78072E-06	27840	tags=17%, list=23%, signal=13%
R-HSA-167158	Formation of the HIV-1 Early Elongation Complex	29	0.473173716	9.09511E-07	2.78072E-06	27840	tags=17%, list=23%, signal=13%
R-HSA-3134975	Regulation of innate immune responses to cytosolic DNA	12	0.7348987	9.2156E-07	2.79465E-06	23156	tags=17%, list=19%, signal=14%
R-HSA-9013973	TICAM1-dependent activation of IRF3/IRF7	12	0.7348987	9.2156E-07	2.79465E-06	11719	tags=14%, list=10%, signal=13%
R-HSA-111447	Activation of BAD and translocation to mitochondria	15	0.685293307	9.28607E-07	2.80462E-06	43363	tags=44%, list=35%, signal=28%
R-HSA-8934593	Regulation of RUNX1 Expression and Activity	17	0.648392205	9.63846E-07	2.89931E-06	39499	tags=30%, list=32%, signal=20%
R-HSA-1912420	Pre-NOTCH Processing in Golgi	18	0.624032809	9.77997E-07	2.93006E-06	63209	tags=58%, list=52%, signal=28%
R-HSA-9634638	Estrogen-dependent nuclear events downstream of ESR-membrane	22	0.552549918	9.93789E-07	2.96547E-06	39978	tags=38%, list=33%, signal=26%
R-HSA-200425	Carnitine metabolism	13	0.73117061	1.01473E-06	3.0159E-06	43493	tags=63%, list=35%, signal=41%
R-HSA-2173791	TGF-beta receptor signaling in EMT (epithelial to mesenchymal trans	16	0.651898476	1.02304E-06	3.02853E-06	67029	tags=54%, list=55%, signal=24%
R-HSA-9615933	Postmitotic nuclear pore complex (NPC) reformation	25	0.508585706	1.04508E-06	3.08154E-06	36525	tags=25%, list=30%, signal=18%
R-HSA-1660516	Synthesis of PIPs at the early endosome membrane	15	0.682531612	1.0578E-06	3.10169E-06	43148	tags=32%, list=35%, signal=21%
R-HSA-5654706	FRS-mediated FGFR3 signaling	19	0.584559354	1.06023E-06	3.10169E-06	61955	tags=51%, list=51%, signal=25%
R-HSA-5654695	PI-3K cascade:FGFR2	22	0.550570721	1.08084E-06	3.14962E-06	28056	tags=26%, list=23%, signal=20%
R-HSA-4641263	Regulation of FZD by ubiquitination	21	0.549291212	1.20586E-06	3.50027E-06	49844	tags=42%, list=41%, signal=25%
R-HSA-175474	Assembly Of The HIV Virion	16	0.647525393	1.23654E-06	3.57542E-06	42328	tags=41%, list=35%, signal=27%
R-HSA-3772470	Negative regulation of TCF-dependent signaling by WNT ligand antaş	14	0.6875268	1.26444E-06	3.64197E-06	52788	tags=45%, list=43%, signal=26%
R-HSA-1810476	RIP-mediated NFkB activation via ZBP1	16	0.64688822	1.27212E-06	3.65002E-06	48454	tags=34%, list=40%, signal=20%
R-HSA-167160	RNA Pol II CTD phosphorylation and interaction with CE during HIV ii	24	0.505759862	1.32505E-06	3.77286E-06	29256	tags=21%, list=24%, signal=16%
R-HSA-77075	RNA Pol II CTD phosphorylation and interaction with CE	24	0.505759862	1.32505E-06	3.77286E-06	29256	tags=21%, list=24%, signal=16%
R-HSA-9005891	Loss of function of MECP2 in Rett syndrome	12	0.72502527	1.38611E-06	3.77385E-06	58498	tags=44%, list=48%, signal=23%
R-HSA-9005895	Pervasive developmental disorders	12	0.72502527	1.38611E-06	3.77385E-06	58498	tags=44%, list=48%, signal=23%
R-HSA-9675151	Disorders of Developmental Biology	12	0.72502527	1.38611E-06	3.77385E-06	58498	tags=44%, list=48%, signal=23%
R-HSA-9697154	Disorders of Nervous System Development	12	0.72502527	1.38611E-06	3.77385E-06	58498	tags=44%, list=48%, signal=23%
R-HSA-6803211	TP53 Regulates Transcription of Death Receptors and Ligands	12	0.724616615	1.38611E-06	3.77385E-06	76361	tags=61%, list=62%, signal=23%
R-HSA-209543	p75NTR recruits signalling complexes	12	0.724332477	1.38611E-06	3.77385E-06	36375	tags=26%, list=30%, signal=19%
R-HSA-209560	NF-kB is activated and signals survival	12	0.724332477	1.38611E-06	3.77385E-06	35814	tags=32%, list=29%, signal=23%
R-HSA-210745	Regulation of gene expression in beta cells	19	0.577518686	1.35415E-06	3.77385E-06	48433	tags=39%, list=39%, signal=24%
R-HSA-167287	HIV elongation arrest and recovery	27	0.473072096	1.371E-06	3.77385E-06	73984	tags=50%, list=60%, signal=20%
R-HSA-167290	Pausing and recovery of HIV elongation	27	0.473072096	1.371E-06	3.77385E-06	73984	tags=50%, list=60%, signal=20%
R-HSA-202040	G-protein activation	23	0.509110425	1.38405E-06	3.77385E-06	41480	tags=43%, list=34%, signal=28%
R-HSA-428930	Thromboxane signalling through TP receptor	23	0.509018435	1.38405E-06	3.77385E-06	31668	tags=34%, list=26%, signal=25%
R-HSA-4655427	SUMOylation of DNA methylation proteins	17	0.632996331	1.70818E-06	4.63383E-06	47226	tags=35%, list=38%, signal=21%
R-HSA-5655291	Signaling by FGFR4 in disease	11	0.781226643	1.78615E-06	4.82778E-06	43608	tags=38%, list=36%, signal=24%
R-HSA-397795	G-protein beta:gamma signalling	29	0.461086075	1.79319E-06	4.8293E-06	49609	tags=45%, list=40%, signal=27%
R-HSA-9615017	FOXO-mediated transcription of oxidative stress, metabolic and neui	27	0.467602817	1.81493E-06	4.87029E-06	50027	tags=46%, list=41%, signal=27%
R-HSA-9703648	Signaling by FLT3 ITD and TKD mutants	16	0.639775447	1.87739E-06	5.01984E-06	40176	tags=38%, list=33%, signal=26%
R-HSA-1963640	GRB2 events in ERBB2 signaling	16	0.63840724	1.94891E-06	5.19245E-06	41140	tags=44%, list=34%, signal=29%
R-HSA-9725371	Nuclear events stimulated by ALK signaling in cancer	18	0.607392082	1.98624E-06	5.25437E-06	44334	tags=38%, list=36%, signal=24%
R-HSA-2243919	Crosslinking of collagen fibrils	16	0.637663722	1.98467E-06	5.25437E-06	74866	tags=64%, list=61%, signal=25%
R-HSA-8964038	LDL clearance	18	0.606930504	2.00682E-06	5.29006E-06	64761	tags=50%, list=53%, signal=24%
R-HSA-170822	Regulation of Glucokinase by Glucokinase Regulatory Protein	28	0.471528679	2.20338E-06	5.76745E-06	36525	tags=28%, list=30%, signal=20%
R-HSA-5619107	Defective TPR may confer susceptibility towards thyroid papillary ca	28	0.471528679	2.20338E-06	5.76745E-06	36525	tags=28%, list=30%, signal=20%
R-HSA-9609523	Insertion of tail-anchored proteins into the endoplasmic reticulum n	17	0.627551663	2.28245E-06	5.95351E-06	53185	tags=45%, list=43%, signal=26%
R-HSA-1296041	Activation of G protein gated Potassium channels	28	0.470014501	2.33395E-06	6.02467E-06	62919	tags=54%, list=51%, signal=27%
R-HSA-1296059	G protein gated Potassium channels	28	0.470014501	2.33395E-06	6.02467E-06	62919	tags=54%, list=51%, signal=27%
R-HSA-997272	Inhibition of voltage gated Ca2+ channels via Gbeta/gamma subunit	28	0.470014501	2.33395E-06	6.02467E-06	62919	tags=54%, list=51%, signal=27%
R-HSA-167238	Pausing and recovery of Tat-mediated HIV elongation	25	0.490233961	2.44177E-06	6.25965E-06	73984	tags=50%, list=60%, signal=20%
R-HSA-167243	Tat-mediated HIV elongation arrest and recovery	25	0.490233961	2.44177E-06	6.25965E-06	73984	tags=50%, list=60%, signal=20%
R-HSA-9648002	RAS processing	24	0.492207141	2.50768E-06	6.40661E-06	61185	tags=38%, list=50%, signal=19%
R-HSA-199220	Vitamin B5 (pantothenate) metabolism	16	0.631050705	2.52105E-06	6.41879E-06	68001	tags=52%, list=55%, signal=23%
R-HSA-5637810	Constitutive Signaling by EGFRvIII	15	0.664699653	2.58046E-06	6.52551E-06	50604	tags=48%, list=41%, signal=28%
R-HSA-5637812	Signaling by EGFRvIII in Cancer	15	0.664699653	2.58046E-06	6.52551E-06	50604	tags=48%, list=41%, signal=28%
R-HSA-168927	TICAM1, RIP1-mediated IKK complex recruitment	17	0.624185046	2.62768E-06	6.62247E-06	47843	tags=36%, list=39%, signal=22%
R-HSA-1362300	Transcription of E2F targets under negative control by p107 (RBL1) a	15	0.662689393	2.77521E-06	6.97074E-06	4773	tags=14%, list=4%, signal=13%
R-HSA-5654720	PI-3K cascade:FGFR4	19	0.558930905	2.89045E-06	7.23583E-06	66562	tags=54%, list=54%, signal=25%
R-HSA-2025928	Calcineurin activates NFAT	11	0.770162079	2.91533E-06	7.27371E-06	56198	tags=44%, list=46%, signal=24%
R-HSA-5140745	WNT5A-dependent internalization of FZD2, FZD5 and ROR2	13	0.705667576	3.02489E-06	7.52189E-06	62950	tags=48%, list=51%, signal=23%
R-HSA-5654688	SHC-mediated cascade:FGFR1	21	0.530928619	3.07198E-06	7.6136E-06	66282	tags=58%, list=54%, signal=27%
R-HSA-6803205	TP53 regulates transcription of several additional cell death genes w	12	0.706112074	3.13105E-06	7.73432E-06	55608	tags=47%, list=45%, signal=25%
R-HSA-2032785	YAP1- and WWTR1 (TAZ)-stimulated gene expression	14	0.670788462	3.1807E-06	7.83102E-06	53680	tags=45%, list=44%, signal=25%
R-HSA-5654712	FRS-mediated FGFR4 signaling	21	0.53045484	3.19578E-06	7.84226E-06	66562	tags=55%, list=54%, signal=25%
R-HSA-162594	Early Phase of HIV Life Cycle	14	0.669105061	3.24463E-06	7.93604E-06	36597	tags=31%, list=30%, signal=22%
R-HSA-1234158	Regulation of gene expression by Hypoxia-inducible Factor	10	0.75943857	4.01402E-06	9.78582E-06	53026	tags=40%, list=43%, signal=23%
R-HSA-174490	Membrane binding and targetting of GAG proteins	14	0.661060684	4.07985E-06	9.86165E-06	42328	tags=42%, list=35%, signal=28%
R-HSA-174495	Synthesis And Processing Of GAG, GAGPOL Polyproteins	14	0.661060684	4.07985E-06	9.86165E-06	42328	tags=42%, list=35%, signal=28%
R-HSA-5693554	Resolution of D-loop Structures through Synthesis-Dependent Strand	25	0.474818131	4.08479E-06	9.86165E-06	35785	tags=30%, list=29%, signal=21%
R-HSA-196791	Vitamin D (calciferol) metabolism	12	0.700640007	4.1665E-06	1.00159E-05	73824	tags=59%, list=60%, signal=24%
R-HSA-9673767	Signaling by PDGFRA transmembrane, juxtamembrane and kinase do	12	0.699702391	4.21581E-06	1.00159E-05	50582	tags=41%, list=41%, signal=24%
R-HSA-9673770	Signaling by PDGFRA extracellular domain mutants	12	0.699702391	4.21581E-06	1.00159E-05	50582	tags=41%, list=41%, signal=24%
R-HSA-170968	Frs2-mediated activation	12	0.699588276	4.21581E-06	1.00159E-05	69453	tags=61%, list=57%, signal=26%
R-HSA-73863	RNA Polymerase I Transcription Termination	24	0.483553435	4.18059E-06	1.00159E-05	38542	tags=28%, list=31%, signal=19%
R-HSA-170660	Adenylate cyclase activating pathway	10	0.755978053	4.34419E-06	1.02881E-05	65459	tags=63%, list=53%, signal=29%
R-HSA-8956320	Nucleotide biosynthesis	12	0.698749282	4.36373E-06	1.03017E-05	11683	tags=11%, list=10%, signal=10%
R-HSA-5621575	CD209 (DC-SIGN) signaling	20	0.553728782	4.64651E-06	1.09347E-05	58983	tags=51%, list=48%, signal=27%
R-HSA-9706369	Negative regulation of FLT3	15	0.64927612	4.79499E-06	1.12486E-05	34942	tags=36%, list=28%, signal=26%
R-HSA-2299718	Condensation of Prophase Chromosomes	10	0.753713808	4.84065E-06	1.13201E-05	33849	tags=21%, list=28%, signal=15%
R-HSA-73772	RNA Polymerase I Promoter Escape	24	0.480581925	4.96199E-06	1.15676E-05	27307	tags=21%, list=22%, signal=17%
R-HSA-9735871	SARS-CoV-1 targets host intracellular signalling and regulatory pathv	15	0.648210968	4.9907E-06	1.15983E-05	31043	tags=30%, list=25%, signal=23%
R-HSA-9758941	Gastrulation	16	0.615508457	5.35449E-06	1.23603E-05	63268	tags=50%, list=52%, signal=24%
R-HSA-5620916	VxPx cargo-targeting to cilium	20	0.549874659	5.36827E-06	1.23603E-05	55509	tags=44%, list=45%, signal=24%
R-HSA-428542	Regulation of commissural axon pathfinding by SLIT and ROBO	10	0.751135181	5.33712E-06	1.23603E-05	37832	tags=37%, list=31%, signal=26%
R-HSA-389359	CD28 dependent Vav1 pathway	11	0.756546765	5.56891E-06	1.27828E-05	56521	tags=48%, list=46%, signal=26%
R-HSA-8849471	PTK6 Regulates RHO GTPases, RAS GTPase and MAP kinases	14	0.651506657	5.68607E-06	1.30117E-05	40585	tags=36%, list=33%, signal=24%
R-HSA-381771	Synthesis, secretion, and inactivation of Glucagon-like Peptide-1 (GL	19	0.540052399	5.72464E-06	1.30599E-05	61559	tags=41%, list=50%, signal=20%
R-HSA-9022699	MECP2 regulates neuronal receptors and channels	16	0.613280615	5.78572E-06	1.3159E-05	53459	tags=49%, list=44%, signal=27%
R-HSA-9617828	FOXO-mediated transcription of cell cycle genes	15	0.645767524	5.83878E-06	1.31992E-05	49556	tags=41%, list=40%, signal=25%
R-HSA-430116	GP1b-IX-V activation signalling	10	0.749969427	5.83358E-06	1.31992E-05	69501	tags=58%, list=57%, signal=25%
R-HSA-442729	CREB1 phosphorylation through the activation of CaMKII/CaMKK/Ca	10	0.748632365	5.91633E-06	1.32939E-05	63513	tags=50%, list=52%, signal=24%

R-HSA-9768919	NPAS4 regulates expression of target genes	20	0.546280615	5.9096E-06	1.32939E-05	66971	tags=58%, list=55%, signal=27%
R-HSA-392451	G beta:gamma signalling through PI3Kgamma	24	0.474749138	6.21223E-06	1.39169E-05	39684	tags=37%, list=32%, signal=25%
R-HSA-5684264	MAP3K8 (TPL2)-dependent MAPK1/3 activation	15	0.643556341	6.29544E-06	1.40334E-05	15629	tags=27%, list=13%, signal=24%
R-HSA-174411	Polymerase switching on the C-strand of the telomere	22	0.519498823	6.30185E-06	1.40334E-05	44686	tags=36%, list=36%, signal=23%
R-HSA-168799	Neurotoxicity of clostridium toxins	10	0.744854845	6.41279E-06	1.42379E-05	39419	tags=43%, list=32%, signal=29%
R-HSA-9634285	Constitutive Signaling by Overexpressed ERBB2	10	0.74361023	6.82652E-06	1.51115E-05	44603	tags=31%, list=36%, signal=20%
R-HSA-1181150	Signaling by NODAL	21	0.515279066	7.0467E-06	1.55069E-05	54332	tags=44%, list=44%, signal=25%
R-HSA-429947	Deadenylation of mRNA	21	0.515275434	7.0467E-06	1.55069E-05	69765	tags=52%, list=57%, signal=22%
R-HSA-189445	Metabolism of porphyrins	24	0.471455929	7.14991E-06	1.56877E-05	56029	tags=49%, list=46%, signal=27%
R-HSA-1266695	Interleukin-7 signaling	21	0.514431858	7.17141E-06	1.56888E-05	61263	tags=45%, list=50%, signal=22%
R-HSA-5576893	Phase 2 - plateau phase	14	0.644089346	7.42522E-06	1.61965E-05	55378	tags=54%, list=45%, signal=30%
R-HSA-8875360	InlB-mediated entry of Listeria monocytogenes into host cell	14	0.642365046	7.94174E-06	1.72727E-05	18275	tags=21%, list=15%, signal=18%
R-HSA-9013418	RHOBTB2 GTPase cycle	21	0.509601852	8.04444E-06	1.74452E-05	58902	tags=49%, list=48%, signal=25%
R-HSA-9735869	SARS-CoV-1 modulates host translation machinery	26	0.459691979	8.38651E-06	1.81163E-05	58444	tags=36%, list=48%, signal=19%
R-HSA-9734009	Defective Intrinsic Pathway for Apoptosis	24	0.467812228	8.40247E-06	1.81163E-05	50526	tags=34%, list=41%, signal=20%
R-HSA-5654710	PI-3K cascade:FGFR3	17	0.599769622	8.44539E-06	1.81564E-05	61955	tags=51%, list=51%, signal=25%
R-HSA-606279	Deposition of new CENPA-containing nucleosomes at the centromer	20	0.538565588	8.61624E-06	1.84175E-05	33520	tags=26%, list=27%, signal=19%
R-HSA-774815	Nucleosome assembly	20	0.538565588	8.61624E-06	1.84175E-05	33520	tags=26%, list=27%, signal=19%
R-HSA-70268	Pyruvate metabolism	26	0.459149269	8.70297E-06	1.85498E-05	74137	tags=66%, list=60%, signal=26%
R-HSA-193368	Synthesis of bile acids and bile salts via 7alpha-hydroxycholesterol	22	0.512761732	8.8932E-06	1.89012E-05	44734	tags=48%, list=36%, signal=31%
R-HSA-450321	JNK (c-Jun kinases) phosphorylation and activation mediated by acti	19	0.529192794	8.94902E-06	1.89658E-05	22937	tags=19%, list=19%, signal=16%
R-HSA-9682385	FLT3 signaling in disease	28	0.446657455	9.42824E-06	1.99248E-05	57837	tags=52%, list=47%, signal=27%
R-HSA-110328	Recognition and association of DNA glycosylase with site containing	14	0.637904137	9.62045E-06	2.01272E-05	56391	tags=42%, list=46%, signal=23%
R-HSA-110329	Cleavage of the damaged pyrimidine	14	0.637904137	9.62045E-06	2.01272E-05	56391	tags=42%, list=46%, signal=23%
R-HSA-73928	Depyrimidination	14	0.637904137	9.62045E-06	2.01272E-05	56391	tags=42%, list=46%, signal=23%
R-HSA-425410	Metal ion SLC transporters	24	0.465235783	9.65888E-06	2.01272E-05	69970	tags=56%, list=57%, signal=24%
R-HSA-352230	Amino acid transport across the plasma membrane	29	0.429145618	9.63484E-06	2.01272E-05	56467	tags=51%, list=46%, signal=28%
R-HSA-5654699	SHC-mediated cascade:FGFR2	22	0.511409001	9.83551E-06	2.04381E-05	66282	tags=58%, list=54%, signal=27%
R-HSA-2586552	Signaling by Leptin	11	0.741268262	1.00249E-05	2.07738E-05	57596	tags=46%, list=47%, signal=24%
R-HSA-111471	Apoptotic factor-mediated response	16	0.60060168	1.0184E-05	2.10196E-05	52871	tags=41%, list=43%, signal=23%
R-HSA-9014325	TICAM1,TRAF6-dependent induction of TAK1 complex	10	0.734886717	1.0228E-05	2.10196E-05	927	tags=7%, list=1%, signal=6%
R-HSA-9645460	Alpha-protein kinase 1 signaling pathway	10	0.734886717	1.0228E-05	2.10196E-05	77427	tags=89%, list=63%, signal=33%
R-HSA-204174	Regulation of pyruvate dehydrogenase (PDH) complex	13	0.672983746	1.02842E-05	2.10769E-05	51692	tags=40%, list=42%, signal=23%
R-HSA-3270619	IRF3-mediated induction of type I IFN	10	0.734218186	1.05606E-05	2.15842E-05	19819	tags=15%, list=16%, signal=13%
R-HSA-9755779	SARS-CoV-2 targets host intracellular signalling and regulatory pathv	12	0.677355362	1.08556E-05	2.21264E-05	43363	tags=44%, list=35%, signal=28%
R-HSA-3214842	HDMs demethylate histones	17	0.591380111	1.10797E-05	2.25216E-05	29200	tags=26%, list=24%, signal=20%
R-HSA-452723	Transcriptional regulation of pluripotent stem cells	19	0.52284784	1.18467E-05	2.40154E-05	24039	tags=29%, list=20%, signal=23%
R-HSA-2022928	HS-GAG biosynthesis	28	0.439464752	1.28566E-05	2.5992E-05	53467	tags=46%, list=44%, signal=26%
R-HSA-9008059	Interleukin-37 signaling	20	0.528663757	1.29656E-05	2.61414E-05	62842	tags=52%, list=51%, signal=26%
R-HSA-9664873	Pexophagy	11	0.734892708	1.3334E-05	2.68118E-05	41250	tags=24%, list=34%, signal=16%
R-HSA-1834941	STING mediated induction of host immune responses	11	0.734224172	1.35287E-05	2.71301E-05	19819	tags=15%, list=16%, signal=13%
R-HSA-169893	Prolonged ERK activation events	14	0.625300645	1.46642E-05	2.93285E-05	55088	tags=46%, list=45%, signal=26%
R-HSA-442660	Na+/Cl- dependent neurotransmitter transporters	18	0.565131667	1.49567E-05	2.98334E-05	50336	tags=55%, list=41%, signal=32%
R-HSA-9758274	Regulation of NF-kappa B signaling	17	0.586946596	1.51086E-05	3.0056E-05	57411	tags=47%, list=47%, signal=25%
R-HSA-5656121	Translesion synthesis by POLI	17	0.585457421	1.57284E-05	3.12058E-05	44686	tags=25%, list=36%, signal=16%
R-HSA-9701192	Defective HDR through Homologous Recombination (HRR) due to BR	23	0.462150872	1.60514E-05	3.14287E-05	35785	tags=30%, list=29%, signal=22%
R-HSA-9701193	Defective HDR through Homologous Recombination (HRR) due to PA	23	0.462150872	1.60514E-05	3.14287E-05	35785	tags=30%, list=29%, signal=22%
R-HSA-9704331	Defective HDR through Homologous Recombination Repair (HRR) du	23	0.462150872	1.60514E-05	3.14287E-05	35785	tags=30%, list=29%, signal=22%
R-HSA-9704646	Defective HDR through Homologous Recombination Repair (HRR) du	23	0.462150872	1.60514E-05	3.14287E-05	35785	tags=30%, list=29%, signal=22%
R-HSA-9709603	Impaired BRCA2 binding to PALB2	23	0.462150872	1.60514E-05	3.14287E-05	35785	tags=30%, list=29%, signal=22%
R-HSA-450385	Butyrate Response Factor 1 (BRF1) binds and destabilizes mRNA	16	0.591523811	1.64041E-05	3.19515E-05	27596	tags=39%, list=22%, signal=30%
R-HSA-450513	Tristetraprolin (TTP, ZFP36) binds and destabilizes mRNA	16	0.590864641	1.64041E-05	3.19515E-05	16461	tags=18%, list=13%, signal=16%
R-HSA-6785631	ERBB2 Regulates Cell Motility	15	0.616104768	1.6471E-05	3.19984E-05	41751	tags=45%, list=34%, signal=30%
R-HSA-937041	IKK complex recruitment mediated by RIP1	22	0.49671817	1.74007E-05	3.37168E-05	47843	tags=36%, list=39%, signal=22%
R-HSA-70895	Branched-chain amino acid catabolism	20	0.523011531	1.76803E-05	3.41696E-05	33316	tags=23%, list=27%, signal=17%
R-HSA-5654719	SHC-mediated cascade:FGFR4	19	0.513252975	1.8329E-05	3.53319E-05	66562	tags=57%, list=54%, signal=26%
R-HSA-9754560	SARS-CoV-2 modulates autophagy	11	0.723964232	1.8395E-05	3.53677E-05	27414	tags=18%, list=22%, signal=14%
R-HSA-918233	TRAF3-dependent IRF activation pathway	13	0.660925805	1.90712E-05	3.65735E-05	59553	tags=39%, list=49%, signal=20%
R-HSA-5655862	Translesion synthesis by POLK	17	0.58052416	1.94657E-05	3.72345E-05	19658	tags=27%, list=16%, signal=22%
R-HSA-5576886	Phase 4 - resting membrane potential	19	0.512154907	1.96994E-05	3.7585E-05	71132	tags=67%, list=58%, signal=28%
R-HSA-5654704	SHC-mediated cascade:FGFR3	17	0.57707217	2.1023E-05	4.0008E-05	61955	tags=54%, list=51%, signal=27%
R-HSA-193692	Regulated proteolysis of p75NTR	11	0.719051657	2.17137E-05	4.10087E-05	56699	tags=40%, list=46%, signal=21%
R-HSA-9013700	NOTCH4 Activation and Transmission of Signal to the Nucleus	11	0.719051657	2.17137E-05	4.10087E-05	66532	tags=53%, list=54%, signal=24%
R-HSA-392170	ADP signalling through P2Y purinoceptor 12	21	0.489620572	2.1683E-05	4.10087E-05	66284	tags=61%, list=54%, signal=28%
R-HSA-75035	Chk1/Chk2(Cds1) mediated inactivation of Cyclin B:Cdk1 complex	13	0.656964768	2.20403E-05	4.15203E-05	31043	tags=26%, list=25%, signal=19%
R-HSA-8956321	Nucleotide salvage	21	0.488862254	2.29363E-05	4.30995E-05	36142	tags=43%, list=29%, signal=30%
R-HSA-73980	RNA Polymerase III Transcription Termination	22	0.489937086	2.3556E-05	4.41528E-05	58215	tags=42%, list=47%, signal=22%
R-HSA-917977	Transferrin endocytosis and recycling	29	0.413668379	2.44533E-05	4.57197E-05	40536	tags=35%, list=33%, signal=23%
R-HSA-9619483	Activation of AMPK downstream of NMDARs	28	0.42317379	2.50452E-05	4.67093E-05	70103	tags=52%, list=57%, signal=22%
R-HSA-8964539	Glutamate and glutamine metabolism	11	0.711257501	2.5626E-05	4.76734E-05	35276	tags=29%, list=29%, signal=21%
R-HSA-72086	mRNA Capping	26	0.436541065	2.62077E-05	4.86343E-05	29256	tags=21%, list=24%, signal=16%
R-HSA-2424491	DAP12 signaling	28	0.418305715	3.0629E-05	5.66979E-05	57499	tags=52%, list=47%, signal=27%
R-HSA-110312	Translesion synthesis by REV1	16	0.572002005	3.18031E-05	5.87255E-05	44686	tags=27%, list=36%, signal=17%
R-HSA-9637690	Response of Mtb to phagocytosis	22	0.482006836	3.40217E-05	6.26672E-05	31657	tags=23%, list=26%, signal=17%
R-HSA-9683610	Maturation of nucleoprotein	11	0.700634294	3.5798E-05	6.57767E-05	30320	tags=25%, list=25%, signal=19%
R-HSA-74749	Signal attenuation	10	0.699576869	3.59427E-05	6.58802E-05	56256	tags=50%, list=46%, signal=27%
R-HSA-8866427	VLDLR internalisation and degradation	16	0.56853443	3.64713E-05	6.65741E-05	65787	tags=43%, list=54%, signal=20%
R-HSA-389957	Prefoldin mediated transfer of substrate to CCT/TriC	25	0.429828281	3.64998E-05	6.65741E-05	24097	tags=17%, list=20%, signal=14%
R-HSA-4755510	SUMOylation of immune response proteins	10	0.695687975	3.79581E-05	6.90426E-05	86718	tags=65%, list=71%, signal=19%
R-HSA-5620922	BBSome-mediated cargo-targeting to cilium	21	0.476398146	3.80382E-05	6.90426E-05	18520	tags=23%, list=15%, signal=20%
R-HSA-69186	Lagging Strand Synthesis	19	0.490164983	4.09704E-05	7.41843E-05	44950	tags=34%, list=37%, signal=21%
R-HSA-9614399	Regulation of localization of FOXO transcription factors	10	0.692850795	4.13172E-05	7.4631E-05	46827	tags=50%, list=38%, signal=31%
R-HSA-451306	Ionotropic activity of kainate receptors	11	0.688373551	4.99325E-05	8.97582E-05	57222	tags=61%, list=47%, signal=33%
R-HSA-451308	Activation of Ca-permeable Kainate Receptor	11	0.688373551	4.99325E-05	8.97582E-05	57222	tags=61%, list=47%, signal=33%
R-HSA-1250347	SHC1 events in ERBB4 signaling	14	0.591557448	5.03389E-05	9.02712E-05	41751	tags=44%, list=34%, signal=29%
R-HSA-193807	Synthesis of bile acids and bile salts via 27-hydroxycholesterol	13	0.638438753	5.12065E-05	9.16069E-05	57973	tags=63%, list=47%, signal=33%
R-HSA-1483115	Hydrolysis of LPC	10	0.68600243	5.34097E-05	9.53198E-05	66856	tags=63%, list=55%, signal=29%
R-HSA-8949664	Processing of SMDT1	13	0.635308982	5.58196E-05	9.93829E-05	41526	tags=32%, list=34%, signal=21%
R-HSA-140837	Intrinsic Pathway of Fibrin Clot Formation	18	0.529685281	5.70437E-05	0.00010132	75019	tags=63%, list=61%, signal=24%
R-HSA-171007	p38MAPK events	13	0.634850881	5.85875E-05	0.000103815	67864	tags=61%, list=55%, signal=27%
R-HSA-74217	Purine salvage	12	0.632260425	5.91398E-05	0.000104546	25818	tags=30%, list=21%, signal=24%
R-HSA-5213460	RIPK1-mediated regulated necrosis	26	0.416559957	6.03826E-05	0.000106239	58663	tags=40%, list=48%, signal=21%
R-HSA-5675482	Regulation of necroptotic cell death	26	0.416559957	6.03826E-05	0.000106239	58663	tags=40%, list=48%, signal=21%
R-HSA-5358606	Mismatch repair (MMR) directed by MSH2:MSH3 (MutSbeta)	13	0.633696581	6.22779E-05	0.000109316	44950	tags=35%, list=37%, signal=22%
R-HSA-1295596	Spry regulation of FGF signaling	16	0.553277185	6.39202E-05	0.000111935	39003	tags=24%, list=32%, signal=17%
R-HSA-9748787	Azathioprine ADME	20	0.496148079	6.48285E-05	0.00011326	52519	tags=43%, list=43%, signal=25%
R-HSA-156711	Polo-like kinase mediated events	15	0.569247617	6.54718E-05	0.000114117	3695	tags=7%, list=3%, signal=7%
R-HSA-8984722	Interleukin-35 Signalling	12	0.627850475	6.71859E-05	0.000116831	59059	tags=43%, list=48%, signal=22%
R-HSA-180292	GAB1 signalosome	16	0.549536726	6.86115E-05	0.000119033	43273	tags=42%, list=35%, signal=27%
R-HSA-450604	KSRP (KHSRP) binds and destabilizes mRNA	15	0.56597523	7.07946E-05	0.000122535	60593	tags=46%, list=49%, signal=23%
R-HSA-5358508	Mismatch Repair	14	0.578622469	7.39157E-05	0.000127641	44950	tags=35%, list=37%, signal=22%
R-HSA-69183	Processive synthesis on the lagging strand	14	0.578126911	7.49641E-05	0.000129153	44950	tags=33%, list=37%, signal=21%
R-HSA-1502540	Signaling by Activin	15	0.563423344	7.61174E-05	0.000130838	31616	tags=27%, list=26%, signal=20%
R-HSA-9028731	Activated NTRK2 signals through FRS2 and FRS3	11	0.673713475	7.98129E-05	0.000136874	39345	tags=26%, list=32%, signal=18%
R-HSA-9754189	Germ layer formation at gastrulation	14	0.575733596	8.44E-05	0.000144409	62842	tags=53%, list=51%, signal=26%
R-HSA-9634600	Regulation of glycolysis by fructose 2,6-bisphosphate metabolism	11	0.670706807	9.24561E-05	0.000157831	60921	tags=42%, list=50%, signal=21%
R-HSA-9768759	Regulation of NPAS4 gene expression	13	0.620663644	9.36471E-05	0.000159471	32936	tags=26%, list=27%, signal=19%
R-HSA-196780	Biotin transport and metabolism	10	0.66880814	9.38444E-05	0.000159471	59406	tags=48%, list=48%, signal=25%
R-HSA-9733458	Induction of Cell-Cell Fusion	12	0.617137499	9.46062E-05	0.000160401	51420	tags=49%, list=42%, signal=29%
R-HSA-163560	Triglyceride catabolism	20	0.483670152	9.49629E-05	0.00016064	71415	tags=67%, list=58%, signal=28%
R-HSA-8862803	Deregulated CDK5 triggers multiple neurodegenerative pathways in	21	0.450848822	9.87293E-05	0.000166257	50526	tags=36%, list=41%, signal=21%

R-HSA-8863678	Neurodegenerative Diseases	21	0.450848822	9.87293E-05	0.000166257	50526	tags=36%, list=41%, signal=21%
R-HSA-446107	Type I hemidesmosome assembly	11	0.666229129	0.000100358	0.00016862	62564	tags=62%, list=51%, signal=30%
R-HSA-1482788	Acyl chain remodelling of PC	26	0.405103943	0.000102426	0.000171708	60481	tags=67%, list=49%, signal=34%
R-HSA-1660514	Synthesis of PIPs at the Golgi membrane	17	0.539202707	0.000106312	0.000177425	42150	tags=35%, list=34%, signal=23%
R-HSA-75876	Synthesis of very long-chain fatty acyl-CoAs	23	0.42345384	0.000106155	0.000177425	50658	tags=44%, list=41%, signal=26%
R-HSA-354194	GRB2:SOS provides linkage to MAPK signaling for Integrins	14	0.568623408	0.000108524	0.000180712	22339	tags=24%, list=18%, signal=20%
R-HSA-9702518	STAT5 activation downstream of FLT3 ITD mutants	10	0.659739889	0.0001222	0.000203031	65614	tags=61%, list=53%, signal=29%
R-HSA-425561	Sodium/Calcium exchangers	13	0.613720882	0.000123317	0.000204432	55942	tags=53%, list=46%, signal=29%
R-HSA-8866904	Negative regulation of activity of TFAP2 (AP-2) family transcription fa	10	0.657410502	0.000127601	0.000211065	82854	tags=74%, list=68%, signal=24%
R-HSA-9027276	Erythropoietin activates Phosphoinositide-3-kinase (PI3K)	11	0.655418406	0.000143029	0.000235541	45534	tags=40%, list=37%, signal=25%
R-HSA-9635486	Infection with Mycobacterium tuberculosis	25	0.403025378	0.000142954	0.000235541	31657	tags=25%, list=26%, signal=18%
R-HSA-5625970	RHO GTPases activate KTN1	11	0.65327339	0.000147771	0.000242813	48972	tags=42%, list=40%, signal=25%
R-HSA-2214320	Anchoring fibril formation	13	0.609642574	0.000149278	0.00024475	65370	tags=58%, list=53%, signal=27%
R-HSA-400508	Incretin synthesis, secretion, and inactivation	22	0.440390061	0.000154733	0.000253138	61559	tags=42%, list=50%, signal=21%
R-HSA-9755088	Ribavirin ADME	12	0.600220048	0.000164145	0.000267947	44429	tags=48%, list=36%, signal=30%
R-HSA-162658	Golgi Cisternae Pericentriolar Stack Reorganization	13	0.602809529	0.000165967	0.00027033	33403	tags=17%, list=27%, signal=13%
R-HSA-3781860	Diseases associated with N-glycosylation of proteins	19	0.454235453	0.000173849	0.000282552	54760	tags=37%, list=45%, signal=20%
R-HSA-1482801	Acyl chain remodelling of PS	21	0.436125842	0.000176046	0.000285501	57101	tags=56%, list=47%, signal=30%
R-HSA-197264	Nicotinamide salvaging	17	0.526872692	0.00018586	0.000300762	39147	tags=38%, list=32%, signal=26%
R-HSA-76071	RNA Polymerase III Transcription Initiation From Type 3 Promoter	27	0.380280684	0.000188565	0.000304479	71767	tags=47%, list=59%, signal=20%
R-HSA-77387	Insulin receptor recycling	25	0.396485913	0.000191046	0.000307819	73719	tags=64%, list=60%, signal=25%
R-HSA-5357786	TNFR1-induced proapoptotic signaling	13	0.597037194	0.0001994	0.000320587	67425	tags=56%, list=55%, signal=25%
R-HSA-9651496	Defects of contact activation system (CAS) and kallikrein/kinin systen	11	0.642063984	0.000201707	0.000322905	71665	tags=65%, list=58%, signal=27%
R-HSA-9671793	Diseases of hemostasis	11	0.642063984	0.000201707	0.000322905	71665	tags=65%, list=58%, signal=27%
R-HSA-936964	Activation of IRF3/IRF7 mediated by TBK1/IKK epsilon	18	0.496701971	0.000203164	0.00032454	23086	tags=64%, list=19%, signal=52%
R-HSA-8847993	ERBB2 Activates PTK6 Signaling	13	0.596276586	0.000206854	0.000329729	52194	tags=54%, list=43%, signal=31%
R-HSA-6803529	FGFR2 alternative splicing	25	0.394952674	0.000215114	0.000342165	66826	tags=49%, list=54%, signal=22%
R-HSA-5357769	Caspase activation via extrinsic apoptotic signalling pathway	26	0.385040405	0.000219703	0.00034872	57411	tags=50%, list=47%, signal=27%
R-HSA-392154	Nitric oxide stimulates guanylate cyclase	18	0.494262828	0.00022498	0.000356337	50299	tags=55%, list=41%, signal=32%
R-HSA-933543	NF-kB activation through FADD/RIP-1 pathway mediated by caspase-	11	0.634462638	0.000249354	0.000394106	59410	tags=52%, list=48%, signal=27%
R-HSA-1482839	Acyl chain remodelling of PE	24	0.392080239	0.000257568	0.000406228	57101	tags=51%, list=47%, signal=27%
R-HSA-5423646	Aflatoxin activation and detoxification	17	0.519877701	0.000261745	0.000411077	48127	tags=43%, list=39%, signal=26%
R-HSA-73621	Pyrimidine catabolism	12	0.58896906	0.000261654	0.000411077	43059	tags=52%, list=35%, signal=34%
R-HSA-76061	RNA Polymerase III Transcription Initiation From Type 1 Promoter	27	0.373221993	0.000264681	0.000414815	67097	tags=42%, list=55%, signal=19%
R-HSA-212300	PRC2 methylates histones and DNA	11	0.631800031	0.000277942	0.000434685	51840	tags=46%, list=42%, signal=27%
R-HSA-190241	FGFR2 ligand binding and activation	19	0.442422197	0.000279547	0.000436281	28056	tags=28%, list=23%, signal=21%
R-HSA-2022923	Dermatan sulfate biosynthesis	10	0.637861899	0.000286368	0.000445993	38820	tags=45%, list=32%, signal=31%
R-HSA-209968	Thyroxine biosynthesis	10	0.636090888	0.000291822	0.000453541	80312	tags=70%, list=65%, signal=24%
R-HSA-8978934	Metabolism of cofactors	18	0.485255024	0.000293235	0.000454789	67180	tags=54%, list=55%, signal=24%
R-HSA-9020558	Interleukin-2 signaling	11	0.628212236	0.000300177	0.000464589	60117	tags=55%, list=49%, signal=28%
R-HSA-209776	Metabolism of amine-derived hormones	17	0.516407857	0.000302403	0.000466602	42355	tags=38%, list=35%, signal=25%
R-HSA-1059683	Interleukin-6 signaling	11	0.627902426	0.000303354	0.000466602	59059	tags=48%, list=48%, signal=25%
R-HSA-9020956	Interleukin-27 signaling	11	0.627845356	0.000303354	0.000466602	60782	tags=49%, list=50%, signal=25%
R-HSA-176974	Unwinding of DNA	12	0.579511638	0.000310408	0.00047647	47425	tags=37%, list=39%, signal=23%
R-HSA-5654219	Phospholipase C-mediated cascade: FGFR1	16	0.509596616	0.000315065	0.000482625	68505	tags=63%, list=56%, signal=28%
R-HSA-2024096	HS-GAG degradation	19	0.436720706	0.000315872	0.000482869	51525	tags=48%, list=42%, signal=28%
R-HSA-211976	Endogenous sterols	24	0.387298262	0.000347697	0.000530434	43582	tags=48%, list=36%, signal=31%
R-HSA-202670	ERKs are inactivated	13	0.579437169	0.000359662	0.000547567	108188	tags=100%, list=88%, signal=12%
R-HSA-5358565	Mismatch repair (MMR) directed by MSH2:MSH6 (MutSalpha)	13	0.578122197	0.000370843	0.000562294	44950	tags=32%, list=37%, signal=20%
R-HSA-69166	Removal of the Flap Intermediate	13	0.578122197	0.000370843	0.000562294	44620	tags=33%, list=36%, signal=21%
R-HSA-2033519	Activated point mutants of FGFR2	16	0.505116143	0.000371918	0.00056278	66282	tags=59%, list=54%, signal=27%
R-HSA-8964616	G beta:gamma signalling through CDC42	18	0.479046622	0.000386411	0.000582276	65891	tags=58%, list=54%, signal=27%
R-HSA-1170546	Prolactin receptor signaling	12	0.572807947	0.000388704	0.000582276	27100	tags=31%, list=22%, signal=24%
R-HSA-937039	IRAK1 recruits IKK complex	12	0.572644731	0.000388704	0.000582276	48913	tags=39%, list=40%, signal=24%
R-HSA-975144	IRAK1 recruits IKK complex upon TLR7/8 or 9 stimulation	12	0.572644731	0.000388704	0.000582276	48913	tags=39%, list=40%, signal=24%
R-HSA-111461	Cytochrome c-mediated apoptotic response	12	0.572540867	0.000388704	0.000582276	37039	tags=34%, list=30%, signal=24%
R-HSA-549132	Organic cation/anion/zwitterion transport	14	0.535421066	0.000391766	0.000585686	47214	tags=51%, list=38%, signal=31%
R-HSA-389958	Cooperation of Prefoldin and TriC/CCT in actin and tubulin folding	29	0.358069477	0.000394426	0.000588483	60898	tags=42%, list=50%, signal=21%
R-HSA-110056	MAPK3 (ERK1) activation	10	0.627897307	0.000406367	0.000604837	60782	tags=45%, list=50%, signal=23%
R-HSA-167242	Abortive elongation of HIV-1 transcript in the absence of Tat	21	0.414569584	0.000407008	0.000604837	37844	tags=23%, list=31%, signal=16%
R-HSA-196299	Beta-catenin phosphorylation cascade	16	0.500858912	0.000433509	0.000642937	50928	tags=35%, list=42%, signal=20%
R-HSA-70221	Glycogen breakdown (glycogenolysis)	13	0.571231081	0.000438223	0.00064864	13429	tags=19%, list=11%, signal=17%
R-HSA-5654221	Phospholipase C-mediated cascade; FGFR2	17	0.505120261	0.000454872	0.000671949	66282	tags=60%, list=54%, signal=28%
R-HSA-937072	TRAF6-mediated induction of TAK1 complex within TLR4 complex	16	0.496693871	0.000480886	0.000707576	69134	tags=55%, list=56%, signal=24%
R-HSA-435354	Zinc transporters	16	0.494975869	0.000480886	0.000707576	69970	tags=51%, list=57%, signal=22%
R-HSA-425381	Bicarbonate transporters	10	0.620470091	0.000493639	0.000724911	64135	tags=60%, list=52%, signal=28%
R-HSA-71064	Lysine catabolism	11	0.609052961	0.000501171	0.000734525	44275	tags=41%, list=36%, signal=26%
R-HSA-9703465	Signaling by FLT3 fusion proteins	19	0.428692771	0.000528312	0.000772785	57837	tags=53%, list=47%, signal=28%
R-HSA-418457	cGMP effects	14	0.526151661	0.00053203	0.000776702	58725	tags=64%, list=48%, signal=33%
R-HSA-8876725	Protein methylation	15	0.510754066	0.000540215	0.000787111	32046	tags=26%, list=26%, signal=19%
R-HSA-9708530	Regulation of BACH1 activity	10	0.61726699	0.000553639	0.000805096	14705	tags=12%, list=12%, signal=11%
R-HSA-1368108	BMAL1:CLOCK,NPAS2 activates circadian gene expression	24	0.375290686	0.000584947	0.00084897	62847	tags=52%, list=51%, signal=26%
R-HSA-9018678	Biosynthesis of specialized proresolving mediators (SPMs)	16	0.488478152	0.000595153	0.000862105	38561	tags=38%, list=31%, signal=26%
R-HSA-5689877	Josephin domain DUBs	10	0.613892107	0.00059749	0.000863813	3620	tags=13%, list=3%, signal=13%
R-HSA-190242	FGFR1 ligand binding and activation	15	0.505112024	0.000609362	0.000879272	68505	tags=62%, list=56%, signal=27%
R-HSA-418217	G beta:gamma signalling through PLC beta	19	0.423137162	0.000646099	0.000930482	50106	tags=46%, list=41%, signal=27%
R-HSA-2162123	Synthesis of Prostaglandins (PG) and Thromboxanes (TX)	13	0.557552139	0.000692912	0.000995978	53810	tags=49%, list=44%, signal=28%
R-HSA-8866910	TFAP2 (AP-2) family regulates transcription of growth factors and the	12	0.550772306	0.000702468	0.001007772	34977	tags=31%, list=29%, signal=22%
R-HSA-8963898	Plasma lipoprotein assembly	18	0.462091008	0.000732574	0.001048944	58973	tags=56%, list=48%, signal=29%
R-HSA-1475029	Reversible hydration of carbon dioxide	11	0.59816071	0.00075066	0.001072783	50487	tags=45%, list=41%, signal=27%
R-HSA-389661	Glyoxylate metabolism and glycine degradation	29	0.343046306	0.000764931	0.001091087	34318	tags=37%, list=28%, signal=26%
R-HSA-422085	Synthesis, secretion, and deacylation of Ghrelin	15	0.49857291	0.000773584	0.001101324	54328	tags=52%, list=44%, signal=29%
R-HSA-975163	IRAK2 mediated activation of TAK1 complex upon TLR7/8 or 9 stimul	15	0.496689822	0.000799514	0.001136072	65122	tags=51%, list=53%, signal=24%
R-HSA-156590	Glutathione conjugation	28	0.351320458	0.000804046	0.001140339	54337	tags=40%, list=44%, signal=22%
R-HSA-75067	Processing of Capped Intronless Pre-mRNA	25	0.363035224	0.00084467	0.001193416	31541	tags=23%, list=26%, signal=17%
R-HSA-1614635	Sulfur amino acid metabolism	25	0.362630674	0.00084467	0.001193416	17569	tags=59%, list=14%, signal=51%
R-HSA-8934903	Receptor Mediated Mitophagy	10	0.599486803	0.000871561	0.001229082	76940	tags=61%, list=63%, signal=23%
R-HSA-190377	FGFR2b ligand binding and activation	10	0.599206162	0.000882524	0.001242194	32902	tags=32%, list=27%, signal=23%
R-HSA-5658623	FGFRL1 modulation of FGFR1 signaling	13	0.533404924	0.000971109	0.00136211	28056	tags=24%, list=23%, signal=19%
R-HSA-76066	RNA Polymerase III Transcription Initiation From Type 2 Promoter	26	0.351507281	0.000971371	0.00136211	67097	tags=43%, list=55%, signal=19%
R-HSA-5654228	Phospholipase C-mediated cascade; FGFR4	14	0.505107906	0.000983772	0.001376912	67350	tags=61%, list=55%, signal=28%
R-HSA-392851	Prostacyclin signalling through prostacyclin receptor	18	0.452162439	0.001018989	0.00142087	57183	tags=56%, list=47%, signal=30%
R-HSA-75892	Platelet Adhesion to exposed collagen	14	0.503877535	0.001017989	0.00142087	57999	tags=58%, list=47%, signal=31%
R-HSA-1855183	Synthesis of IP2, IP, and Ins in the cytosol	13	0.528814737	0.001076498	0.001498261	57311	tags=45%, list=47%, signal=24%
R-HSA-5654227	Phospholipase C-mediated cascade; FGFR3	12	0.535208593	0.001148886	0.001596032	65234	tags=60%, list=53%, signal=28%
R-HSA-9670095	Inhibition of DNA recombination at telomere	17	0.464494051	0.001159996	0.00160847	38177	tags=28%, list=31%, signal=19%
R-HSA-189451	Heme biosynthesis	13	0.525945695	0.001181888	0.001635786	56029	tags=45%, list=46%, signal=24%
R-HSA-196741	Cobalamin (Cbl, vitamin B12) transport and metabolism	20	0.41556214	0.001201339	0.001659628	22792	tags=26%, list=19%, signal=21%
R-HSA-9660826	Purinergic signaling in leishmaniasis infection	25	0.35613807	0.001209271	0.00166442	30107	tags=30%, list=25%, signal=23%
R-HSA-9664424	Cell recruitment (pro-inflammatory response)	25	0.35613807	0.001209271	0.00166442	30107	tags=30%, list=25%, signal=23%
R-HSA-1250342	PI3K events in ERBB4 signaling	10	0.585820925	0.001289016	0.001767658	42603	tags=50%, list=35%, signal=32%
R-HSA-2206281	Mucopolysaccharidoses	10	0.585437439	0.001289016	0.001767658	73845	tags=58%, list=60%, signal=23%
R-HSA-2046104	alpha-linolenic (omega3) and linoleic (omega6) acid metabolism	12	0.530620002	0.001332706	0.001817548	49665	tags=34%, list=40%, signal=20%
R-HSA-2046106	alpha-linolenic acid (ALA) metabolism	12	0.530620002	0.001332706	0.001817548	49665	tags=34%, list=40%, signal=20%
R-HSA-189483	Heme degradation	13	0.52177755	0.001332445	0.001817548	42686	tags=42%, list=35%, signal=28%
R-HSA-2465910	MASTL Facilitates Mitotic Progression	10	0.580410273	0.00142122	0.001934726	64049	tags=40%, list=52%, signal=19%
R-HSA-6783589	Interleukin-6 family signaling	24	0.354654564	0.001466982	0.001993385	59059	tags=53%, list=48%, signal=27%
R-HSA-8964058	HDL remodeling	10	0.57779825	0.001487322	0.002015727	54028	tags=56%, list=44%, signal=31%
R-HSA-933542	TRAF6 mediated NF-kB activation	23	0.362985552	0.001488828	0.002015727	50206	tags=40%, list=41%, signal=24%
R-HSA-8964315	G beta:gamma signalling through BTK	16	0.456323095	0.001521539	0.002056282	65891	tags=59%, list=54%, signal=27%
R-HSA-2151209	Activation of PPARGC1A (PGC-1alpha) by phosphorylation	10	0.576307119	0.00153139	0.002065853	49331	tags=45%, list=40%, signal=27%

R-HSA-442380	Zinc influx into cells by the SLC39 gene family	10	0.575895147	0.001575458	0.002121465	24417	tags=50%, list=20%, signal=40%
R-HSA-391903	Eicosanoid ligand-binding receptors	14	0.489548411	0.001616795	0.002173205	171	tags=11%, list=0%, signal=11%
R-HSA-190239	FGFR3 ligand binding and activation	12	0.526659809	0.00162299	0.002173699	61955	tags=55%, list=51%, signal=27%
R-HSA-190372	FGFR3c ligand binding and activation	12	0.526659809	0.00162299	0.002173699	61955	tags=55%, list=51%, signal=27%
R-HSA-9037629	Lewis blood group biosynthesis	18	0.4353579	0.00171593	0.002294057	65390	tags=58%, list=53%, signal=27%
R-HSA-2142845	Hyaluronan metabolism	17	0.451020574	0.00177591	0.002369998	47622	tags=47%, list=39%, signal=29%
R-HSA-8875555	MET activates RAP1 and RAC1	11	0.570895763	0.001792422	0.002387762	45812	tags=46%, list=37%, signal=29%
R-HSA-111458	Formation of apoptosome	10	0.572531531	0.001817832	0.002412994	37039	tags=34%, list=30%, signal=24%
R-HSA-9627069	Regulation of the apoptosome activity	10	0.572531531	0.001817832	0.002412994	37039	tags=34%, list=30%, signal=24%
R-HSA-196807	Nicotinate metabolism	29	0.327504532	0.001854215	0.002456917	42646	tags=33%, list=35%, signal=21%
R-HSA-8851708	Signaling by FGFR2 IIIa TM	18	0.432219081	0.001959478	0.002591792	24342	tags=54%, list=20%, signal=43%
R-HSA-9018677	Biosynthesis of DHA-derived SPMs	14	0.483061972	0.002011646	0.002656085	38561	tags=40%, list=31%, signal=27%
R-HSA-500657	Presynaptic function of Kainate receptors	20	0.394367244	0.002054167	0.002702661	65891	tags=64%, list=54%, signal=30%
R-HSA-203927	MicroRNA (miRNA) biogenesis	23	0.354203381	0.002051381	0.002702661	57616	tags=40%, list=47%, signal=21%
R-HSA-2197563	NOTCH2 intracellular domain regulates transcription	11	0.564420304	0.0021019	0.002760594	59520	tags=53%, list=49%, signal=27%
R-HSA-174403	Glutathione synthesis and recycling	12	0.519856508	0.002150781	0.002819829	81568	tags=67%, list=66%, signal=22%
R-HSA-190322	FGFR4 ligand binding and activation	13	0.505103788	0.002193889	0.002866271	67350	tags=59%, list=55%, signal=27%
R-HSA-9668328	Sealing of the nuclear envelope (NE) by ESCRT-III	29	0.323048321	0.002191117	0.002866271	26191	tags=21%, list=21%, signal=16%
R-HSA-5578768	Physiological factors	14	0.480111888	0.00225235	0.002937506	79554	tags=68%, list=65%, signal=24%
R-HSA-1187000	Fertilization	19	0.37997927	0.002564593	0.003338894	67252	tags=67%, list=55%, signal=30%
R-HSA-5668599	RHO GTPases Activate NADPH Oxidases	21	0.362289882	0.002573895	0.003345167	33950	tags=30%, list=28%, signal=22%
R-HSA-9033658	Blood group systems biosynthesis	21	0.361504075	0.002640317	0.003425525	56180	tags=51%, list=46%, signal=28%
R-HSA-1368082	RORA activates gene expression	17	0.434746537	0.00270282	0.003500528	64774	tags=51%, list=53%, signal=24%
R-HSA-429958	mRNA decay by 3' to 5' exoribonuclease	11	0.553931795	0.0027732	0.003585454	24264	tags=33%, list=20%, signal=27%
R-HSA-113501	Inhibition of replication initiation of damaged DNA by RB1/E2F1	11	0.553254631	0.002876868	0.003713051	26680	tags=37%, list=22%, signal=29%
R-HSA-9694614	Attachment and Entry	15	0.448600263	0.00291548	0.003756387	43583	tags=49%, list=36%, signal=32%
R-HSA-3656237	Defective EXT2 causes exostoses 2	12	0.506819629	0.003049851	0.003915987	52565	tags=49%, list=43%, signal=28%
R-HSA-3656253	Defective EXT1 causes exostoses 1, TRPS2 and CHDS	12	0.506819629	0.003049851	0.003915987	52565	tags=49%, list=43%, signal=28%
R-HSA-190375	FGFR2c ligand binding and activation	12	0.50509967	0.00315593	0.004043357	66282	tags=62%, list=54%, signal=29%
R-HSA-2730905	Role of LAT2/NTAL/LAB on calcium mobilization	15	0.444930292	0.003159889	0.004043357	58985	tags=58%, list=48%, signal=30%
R-HSA-449836	Other interleukin signaling	22	0.356090328	0.003183687	0.004066833	64422	tags=64%, list=53%, signal=30%
R-HSA-1971475	A tetrasaccharide linker sequence is required for GAG synthesis	23	0.341991249	0.003331113	0.004247881	51525	tags=54%, list=42%, signal=31%
R-HSA-6803207	TP53 Regulates Transcription of Caspase Activators and Caspases	11	0.541895378	0.003498878	0.004446615	54952	tags=48%, list=45%, signal=27%
R-HSA-9648895	Response of EIF2AK1 (HRI) to heme deficiency	13	0.488259657	0.003497162	0.004446615	66930	tags=50%, list=55%, signal=23%
R-HSA-8875656	MET receptor recycling	10	0.545369757	0.00352076	0.004466815	44643	tags=41%, list=36%, signal=26%
R-HSA-420029	Tight junction interactions	27	0.318759022	0.003585199	0.004540846	35408	tags=35%, list=29%, signal=25%
R-HSA-389977	Post-chaperonin tubulin folding pathway	22	0.343837814	0.004003226	0.005061706	70103	tags=51%, list=57%, signal=22%
R-HSA-389960	Formation of tubulin folding intermediates by CCT/TriC	23	0.336260139	0.004274322	0.005395337	18133	tags=16%, list=15%, signal=13%
R-HSA-1483213	Synthesis of PE	12	0.489678347	0.004641044	0.005848342	52284	tags=47%, list=43%, signal=27%
R-HSA-180336	SHC1 events in EGFR signaling	13	0.477730209	0.004835178	0.006082702	41300	tags=41%, list=34%, signal=27%
R-HSA-1660517	Synthesis of PIPs at the late endosome membrane	10	0.530148129	0.004940088	0.006204218	69212	tags=59%, list=56%, signal=26%
R-HSA-5621480	Dectin-2 family	25	0.317444269	0.004983162	0.006247796	64935	tags=68%, list=53%, signal=32%
R-HSA-166786	Creation of C4 and C2 activators	14	0.448891185	0.005148957	0.006444836	66289	tags=69%, list=54%, signal=32%
R-HSA-1296346	Tandem pore domain potassium channels	12	0.485728262	0.005224483	0.006528416	65364	tags=67%, list=53%, signal=31%
R-HSA-68884	Mitotic Telophase/Cytokinesis	10	0.525638344	0.005474137	0.006828941	23805	tags=15%, list=19%, signal=12%
R-HSA-156581	Methylation	13	0.470166346	0.005504188	0.006854965	24857	tags=28%, list=20%, signal=22%
R-HSA-3560801	Defective B3GAT3 causes JDSSDHD	17	0.406890744	0.005681139	0.00706355	51525	tags=51%, list=42%, signal=30%
R-HSA-977068	Termination of O-glycan biosynthesis	24	0.31744168	0.005757912	0.007147092	53035	tags=49%, list=43%, signal=28%
R-HSA-1839130	Signaling by activated point mutants of FGFR3	11	0.519672764	0.005990508	0.007411143	61955	tags=56%, list=51%, signal=28%
R-HSA-2033514	FGFR3 mutant receptor activation	11	0.519672764	0.005990508	0.007411143	61955	tags=56%, list=51%, signal=28%
R-HSA-3000480	Scavenging by Class A Receptors	18	0.402967512	0.006037279	0.00745664	65808	tags=65%, list=54%, signal=30%
R-HSA-372708	p130Cas linkage to MAPK signaling for integrins	14	0.443209414	0.006185646	0.007627259	68659	tags=63%, list=56%, signal=28%
R-HSA-3560783	Defective B4GALT7 causes EDS, progeroid type	17	0.401998094	0.006261684	0.007708277	51525	tags=50%, list=42%, signal=29%
R-HSA-179812	GRB2 events in EGFR signaling	12	0.471938663	0.007195854	0.008843668	41140	tags=43%, list=34%, signal=28%
R-HSA-1839122	Signaling by activated point mutants of FGFR1	11	0.505095552	0.008074118	0.009890463	68342	tags=66%, list=56%, signal=29%
R-HSA-190373	FGFR1c ligand binding and activation	11	0.505095552	0.008074118	0.009890463	68342	tags=66%, list=56%, signal=29%
R-HSA-5601884	PIWI-interacting RNA (piRNA) biogenesis	27	0.298246902	0.008113607	0.009922542	64655	tags=49%, list=53%, signal=23%
R-HSA-8963901	Chylomicron remodeling	10	0.50924937	0.008233408	0.010052574	70873	tags=73%, list=58%, signal=31%
R-HSA-166663	Initial triggering of complement	19	0.338758499	0.008256281	0.010064029	66731	tags=67%, list=54%, signal=31%
R-HSA-196108	Pregnenolone biosynthesis	10	0.507959476	0.008411426	0.010236417	77699	tags=56%, list=63%, signal=20%
R-HSA-2024101	CS/DS degradation	12	0.461017819	0.009008113	0.01094471	66228	tags=58%, list=54%, signal=27%
R-HSA-73780	RNA Polymerase III Chain Elongation	17	0.386793363	0.009418125	0.011424262	66826	tags=40%, list=54%, signal=18%
R-HSA-351906	Apoptotic cleavage of cell adhesion proteins	11	0.49169845	0.010261916	0.012387361	61879	tags=58%, list=50%, signal=29%
R-HSA-190873	Gap junction degradation	11	0.491643992	0.010261916	0.012387361	72638	tags=54%, list=59%, signal=22%
R-HSA-196025	Formation of annular gap junctions	11	0.491643992	0.010261916	0.012387361	72638	tags=54%, list=59%, signal=22%
R-HSA-8851805	MET activates RAS signaling	11	0.49129147	0.010366097	0.012492905	63375	tags=56%, list=52%, signal=27%
R-HSA-450341	Activation of the AP-1 family of transcription factors	10	0.498979305	0.010644547	0.01278717	60	tags=36%, list=0%, signal=36%
R-HSA-2562578	TRIF-mediated programmed cell death	10	0.496669575	0.010644547	0.01278717	35793	tags=34%, list=29%, signal=24%
R-HSA-1482925	Acyl chain remodelling of PG	18	0.381578965	0.010696087	0.012828426	54084	tags=52%, list=44%, signal=29%
R-HSA-5223345	Miscellaneous transport and binding events	21	0.324456673	0.010799993	0.012932254	66754	tags=58%, list=54%, signal=27%
R-HSA-4419969	Depolymerisation of the Nuclear Lamina	14	0.418997878	0.011182213	0.013368479	69998	tags=65%, list=57%, signal=28%
R-HSA-73614	Pyrimidine salvage	10	0.488818412	0.011539027	0.013772983	35629	tags=51%, list=29%, signal=36%
R-HSA-69091	Polymerase switching	13	0.442455175	0.011673931	0.013889557	44686	tags=41%, list=36%, signal=26%
R-HSA-69109	Leading Strand Synthesis	13	0.442455175	0.011673931	0.013889557	44686	tags=41%, list=36%, signal=26%
R-HSA-1362409	Mitochondrial iron-sulfur cluster biogenesis	11	0.477286928	0.01224957	0.01455124	56178	tags=40%, list=46%, signal=21%
R-HSA-5365859	RA biosynthesis pathway	20	0.333221263	0.012386636	0.014690669	44127	tags=34%, list=36%, signal=22%
R-HSA-432047	Passive transport by Aquaporins	11	0.471163253	0.013296517	0.015744764	52714	tags=62%, list=43%, signal=35%
R-HSA-205043	NRIF signals cell death from the nucleus	16	0.376534212	0.013441246	0.015890919	46036	tags=31%, list=38%, signal=19%
R-HSA-399997	Acetylcholine regulates insulin secretion	10	0.474227286	0.01440137	0.016999085	29806	tags=37%, list=24%, signal=28%
R-HSA-622312	Inflammasomes	20	0.327246253	0.014532312	0.017126547	50206	tags=42%, list=41%, signal=25%
R-HSA-1237044	Erythrocytes take up carbon dioxide and release oxygen	12	0.434193994	0.015748333	0.018501191	80673	tags=76%, list=66%, signal=26%
R-HSA-1480926	O2/CO2 exchange in erythrocytes	12	0.434193994	0.015748333	0.018501191	80673	tags=76%, list=66%, signal=26%
R-HSA-9690406	Transcriptional regulation of testis differentiation	10	0.464286588	0.016190338	0.018990554	60400	tags=56%, list=49%, signal=29%
R-HSA-5683826	Surfactant metabolism	25	0.292779312	0.016361852	0.019161604	69011	tags=68%, list=56%, signal=30%
R-HSA-8963888	Chylomicron assembly	10	0.462060869	0.016548132	0.019349383	70873	tags=61%, list=58%, signal=26%
R-HSA-844456	The NLRP3 inflammasome	15	0.386502284	0.017434476	0.020353864	76738	tags=65%, list=63%, signal=24%
R-HSA-193775	Synthesis of bile acids and bile salts via 24-hydroxycholesterol	13	0.423960383	0.018549457	0.021621711	40620	tags=42%, list=33%, signal=28%
R-HSA-2514853	Condensation of Prometaphase Chromosomes	10	0.453867288	0.019596434	0.022806458	69498	tags=50%, list=57%, signal=22%
R-HSA-2022870	Chondroitin sulfate biosynthesis	18	0.354926165	0.019691317	0.022881188	33806	tags=43%, list=28%, signal=31%
R-HSA-2168880	Scavenging of heme from plasma	10	0.452198634	0.020315549	0.023569828	71324	tags=63%, list=58%, signal=26%
R-HSA-1482922	Acyl chain remodelling of PI	15	0.373624598	0.022537149	0.026106698	52049	tags=51%, list=42%, signal=29%
R-HSA-6788467	IL-6-type cytokine receptor ligand interactions	17	0.360359385	0.023619593	0.027275877	60782	tags=57%, list=50%, signal=29%
R-HSA-881907	Gastrin-CREB signalling pathway via PKC and MAPK	17	0.359749936	0.023619593	0.027275877	35721	tags=34%, list=29%, signal=24%
R-HSA-193048	Androgen biosynthesis	11	0.440777459	0.023778427	0.027416857	74616	tags=70%, list=61%, signal=28%
R-HSA-189200	Cellular hexose transport	20	0.309326769	0.024895606	0.028660682	46745	tags=48%, list=38%, signal=30%
R-HSA-975110	TRAF6 mediated IRF7 activation in TLR7/8 or 9 signaling	11	0.436855922	0.025040966	0.028783607	58956	tags=48%, list=48%, signal=25%
R-HSA-2160916	Hyaluronan uptake and degradation	12	0.407331263	0.026484582	0.030396151	47622	tags=50%, list=39%, signal=31%
R-HSA-71240	Tryptophan catabolism	12	0.400005168	0.029068389	0.03331032	37176	tags=32%, list=30%, signal=22%
R-HSA-8854691	Interleukin-20 family signaling	21	0.289429939	0.030620872	0.035035537	27100	tags=48%, list=22%, signal=37%
R-HSA-1474151	Tetrahydrobiopterin (BH4) synthesis, recycling, salvage and regulatic	11	0.426335489	0.03177453	0.036299845	66937	tags=56%, list=55%, signal=25%
R-HSA-5260271	Diseases of Immune System	28	0.254388897	0.034666768	0.039483068	40820	tags=38%, list=33%, signal=25%
R-HSA-5602358	Diseases associated with the TLR signaling cascade	28	0.254388897	0.034666768	0.039483068	40820	tags=38%, list=33%, signal=25%
R-HSA-166208	mTORC1-mediated signalling	22	-0.465848465	0.036231884	0.041202722	28126	tags=18%, list=23%, signal=14%
R-HSA-1592389	Activation of Matrix Metalloproteinases	26	0.254531169	0.037818094	0.042941093	76478	tags=76%, list=62%, signal=29%
R-HSA-400511	Synthesis, secretion, and inactivation of Glucose-dependent Insulinc	11	0.417558564	0.041033213	0.046520937	60941	tags=78%, list=50%, signal=39%
R-HSA-77595	Processing of Intronless Pre-mRNAs	16	0.32821244	0.041115463	0.046543452	52406	tags=37%, list=43%, signal=21%

Supplemental 4 - Overlapping DMPs

cgID	gene
cg1981581	A2LD1
cg0325657	AARS
cg2061816	ABCA12
cg1782380	ABLIM2
cg0449792	ADAD2
cg0368609	ADCY6
cg2665507	AGAP1
cg2223439	AGBL4
cg2163961	AGPAT3
cg0325661	AK6
cg2755116	ALPK3
cg1378395	AMBP
cg0709047	ANO10
cg2665332	ANXA13
cg2740284	AP2A2
cg2738077	AP2A2
cg2368149	APBB2
cg2649557	APC2
cg1487063	ARHGAP12
cg0837605	ATAD3A
cg2700113	ATP11A
cg1400187	ATP11A
cg1001764	ATP2B4
cg1006630	AVPI1
cg1822171	AZIN1-AS1
cg0137393	BAT1
cg2638132	BATF3
cg0691100	BAZ2B
cg2223439	BEND5
cg2621476	BRP44L
cg1374727	BTBD2
cg1511968	BTBD9
cg1592412	C14orf159
cg2129220	C15orf24
cg0489704	C16orf38
cg0326966	C17orf53
cg0410966	C1orf21
cg1908648	C1orf210
cg1036565	C2orf89
cg2160770	C6orf176
cg2323776	C7orf20
cg2345106	C7orf50

cg0063636 C7orf60
cg0173239 C9orf116
cg1453131 C9orf25
cg0257707 CABP1
cg0988862 CACNA1I
cg1490458 CALHM3
cg0384978 CAMTA1
cg0331002 CATSPER2
cg1181568 CBS
cg0133573 CC2D1A
cg1122406 CCDC163P
cg1193547 CCDC64
cg2699321 CCDC79
cg2610710 CCDC88B
cg1433199 CCHCR1
cg1266935 CD151
cg1620930 CDC42BPB
cg1532393 CDC42BPG
cg2349569 CEBPD
cg0393399 CFAP97
cg2543775 CHRNA6
cg0040231 CLVS1
cg1715864 CLYBL
cg2740044 CLYBL
cg1715864 CLYBL-AS2
cg0010272 COL18A1
cg2582996 COL18A1
cg1754985 COL18A1
cg1347597 CRYL1
cg0677566 CUX1
cg0130184 CYFIP2
cg2677443 CYTH3
cg1491824 DAG1
cg0225469 DBF4
cg1364744 DEPTOR
cg2573728 DIP2C
cg0145718 DNAJC19
cg2066016 DNMT3L
cg0920891 DOCK8
cg0894187 DTWD2
cg1344413 DYRK1B
cg1266935 EFCAB4A
cg1286490 EFCAB5
cg1204551 EHD4

cg0364906 ELOF1
cg2519530 ENAH
cg2333303 EPB41
cg0099186 ESRP1
cg2478298 EZH1
cg1395040 FAM114A2
cg2007952 FAM13A
cg1553537 FAM180A
cg1776098 FAR1
cg2202200 FARP1
cg2603662 FBLIM1
cg0497116 FBXO34
cg2591652 FCHO2
cg2526853 FDXR
cg1889452 FH
cg1611778 FLJ40434
cg2579609 FLOT1
cg2705256 FLOT2
cg0424669 FN3KRP
cg2628647 FRS2
cg0165131 FXYD6-FXYD2
cg2328740 FZR1
cg2424445 GAB1
cg0347083 GABBR2
cg0739795 GALK2
cg2433464 GATA2
cg1262769 GBX1
cg0167481 GEN1
cg1819549 GIGYF1
cg0653125 GJA8
cg1974296 GLI3
cg2262247 GLTPD1
cg0898533 GMDS-AS1
cg2125192 GML
cg1436447 GNAL
cg2650679 GNAS
cg0644418 GNB3
cg2325938 GRIP2
cg1491473 GRK7
cg0840698 GSTCD
cg0130941 GTF3C6
cg1620280 HDAC4
cg0401189 HDAC4
cg0835610 HDAC4

cg1614570 HIPK1
cg2154524 HMGXB3
cg0889489 HOMER3
cg0114104 HSPA2
cg0545062 IDH2
cg0575161 IFFO1
cg1071283 IFT140
cg0212914 IMPG1
cg2163616 INCENP
cg1327267 IPO7
cg0207827 IQCA1
cg1382827 ITCH
cg2553147 ITIH1
cg2303505 JAKMIP3
cg1422826 KCNJ12
cg2695329 KIAA0182
cg2275143 KIAA1958
cg1412387 KIF2A
cg1046081 KLHDC4
cg0380884 KPNA1
cg1058162 LAMC2
cg2447092 LAPTM4B
cg0547998 LARGE
cg0265979 LDHB
cg0237306 LINC01192
cg1651667 LOC101926892
cg2061816 LOC101928103
cg0039592 LOC145783
cg1852925 LOC90110
cg2656355 LOXL2
cg1348809 LPIN1
cg1560915 MAML1
cg1934303 MAMSTR
cg1650982 MAP4
cg2394538 MBD2
cg1180912 MBD3
cg1460220 MED17
cg0202249 METT10D
cg1395040 MFAP3
cg1912772 MIR103-2
cg1315136 MIR548N
cg1629939 MIR6508
cg2213670 MLST8
cg0173239 MRPS2

cg2093276 MSH4
cg1784672 MTHFD1L
cg0831489 MYBPC1
cg1473269 MYC
cg1921433 MYL9
cg1163540 MYO9B
cg0182165 MYT1L
cg0614660 NADSYN1
cg0740670 NDUFAB1
cg2698769 NFXL1
cg0885528 NKAIN3
cg1052907 NPAS2
cg2595043 NPAS3
cg1524302 NRBP1
cg1898753 NUDCD3
cg1969283 NUP107
cg1665459 OCA2
cg1322332 OPCML
cg0218118 OSBP2
cg1315386 OSGIN2
cg0493566 PACS2
cg1912772 PANK2
cg1207584 PARP4
cg1015124 PC
cg1249228 PCBP3
cg1726249 PCIF1
cg1378119 PDE8A
cg1610006 PEX14
cg1987720 PFDN4
cg1258847 PIGY
cg2121781 PIP4K2C
cg0212973 PLCL2
cg0717267 PLEC1
cg1642774 PLEKHG3
cg0224067 POC1A
cg1912094 POLR1B
cg1906020 PPFIA1
cg0139511 PPIC
cg2713465 PPP4R4
cg2578349 PRDM16
cg1452632 PRKAR1B
cg0922548 PRKCZ
cg1052428 PRKDC
cg1315136 PRKRA

cg1769713 PRR14
cg0153442 PSD2
cg0059369 PTDSS2
cg0875701 PTP4A2
cg1480335 PWWP2A
cg1135977 RAB10
cg1452264 RBM14
cg1452264 RBM14-RBM4
cg1884458 RBM44
cg0379074 RCOR2
cg1030072 REEP3
cg2138113 RERE
cg0612394 RNF125
cg0368012 RNF24
cg2533820 RNF44
cg0029131 RP1L1
cg2650877 RTFDC1
cg0922776 RTN1
cg1650775 RUNX1
cg0996904 SAPS1
cg1964901 SCMH1
cg0324104 SCN1A
cg1244927 SCYL2
cg0399153 SEC24A
cg2374383 SETD4
cg1629939 SH3BGR
cg0294495 SIPA1L3
cg1227175 SLC8A1
cg1134784 SMARCE1
cg1058870 SMYD3
cg2199250 SNAP25
cg2394538 SNORA37
cg1476376 SNRPD3
cg2452233 SNTG1
cg0296326 SPECC1
cg1241816 SPPL3
cg0897185 SPTBN2
cg1774828 SRCIN1
cg0387705 STRIP2
cg1709626 SVIL
cg2702876 SYNGR3
cg1483807 SYNPO
cg0256031 TAF4
cg1400630 TAPBP

cg1027410 TBC1D1
cg2302281 TBXAS1
cg1926397 TEAD4
cg2168543 TH1L
cg2028555 THAP3
cg0807168 TMEM161B
cg1347135 TMEM178
cg1071283 TMEM204
cg2534576 TMEM63A
cg0004784 TMTC2
cg0256382 TNFRSF9
cg1129311 TOM1L2
cg2368111 TRAPPC9
cg1560404 TRHDE
cg1533979 TRIB3
cg1331809 TRIM35
cg0249541 TRIM47
cg1613190 TRIM56
cg1601247 TRRAP
cg0451015 TSEN54
cg2043458 TTBK1
cg2539594 TYK2
cg2718464 UBE2E3
cg0475421 UROS
cg1051126 USF1
cg0904812 USP2
cg1867564 USP34
cg1193484 WASF2
cg1977865 WDYHV1
cg1477448 WTAPP1
cg2159554 XRCC5
cg0331989 ZBTB10
cg2268505 ZBTB40
cg2157160 ZC3H6
cg1221798 ZCCHC2
cg2155121 ZFP41
cg0276106 ZNF138
cg1511851 ZNF683
cg1346046 ZNF709
cg2653339 ZNF766
cg2235569 ZNF791
cg2538156 ZNF831
cg1642172 ZRANB1
